# Chromosome-scale genome assembly provides insights into the molecular mechanisms of tissue development of *Populus wilsonii*

**DOI:** 10.1038/s42003-022-04106-0

**Published:** 2022-10-25

**Authors:** Chaofeng Li, Haitao Xing, Can Li, Yun Ren, Honglei Li, Xue-Qin Wan, Chunlan Lian, Jia-Xuan Mi, Shengkui Zhang

**Affiliations:** 1 Maize Research Institute, Southwest University, Chongqing, 400715 PR China; 2 College of Landscape Architecture and life Science/Institute of special Plants, Chongqing University of Arts and Sciences, Chongqing, 402168 PR China; 3 School of Bioengineering, Qilu University of Technology, Jinan, 250353 Shandong PR China; 4 College of Forestry, Sichuan Agricultural University, Chengdu, 611130 PR China; 5 Asian Research Center for Bioresource and Environmental Sciences, Graduate School of Agricultural and Life Sciences, The University of Tokyo, 1-1-1 Midori-cho, Nishitokyo, Tokyo, 188-0002 Japan

**Keywords:** Genome evolution, Plant evolution

## Abstract

*Populus wilsonii* is an important species of section *Leucoides*, and the natural populations mainly grow in southwest China. In this study, a single genotype of wild *P. wilsonii* was sequenced and assembled at genome size of 477.35 Mb in 19 chromosomes with contig N50 of 16.3 Mb. A total of 38,054 genes were annotated, and 49.95% of the genome was annotated as repetitive elements. Phylogenetic analysis identified that the divergence between *P. wilsonii* and the ancestor of *P. deltoides* and *P. trichocarpa* was 12 (3–23) Mya. 4DTv and Ks distributions supported the occurrence of the salicoid WGD event (~65 Mya). The highly conserved collinearity supports the close evolutionary relationship among these species. Some key enzyme-encoding gene families related to the biosynthesis of lignin and flavonoids were expanded and highly expressed in the stems or leaves, which probably resist the damage of the natural environment. In addition, some key gene families related to cellulose biosynthesis were highly expressed in stems, accounting for the high cellulose content of *P. wilsonii* variety. Our findings provided deep insights into the genetic evolution of *P. wilsonii* and will contribute to further biological research and breeding as well as for other poplars in *Salicaceae*.

## Abstract (web summary)

A genome assembly for the Chinese poplar tree, *Populus wilsonii*, provides a unique resource to guide research into poplar development and breeding efforts.

## Introduction

Members of the genus *Populus* have emerged as one of the most widely planted fast-growing tree species in the world. They are mainly distributed in the temperate and cold temperate regions of the northern hemisphere, and have the ecological and economic importance^1^. *Populus* has >100 wild and cultivated species in the world. According to the morphological characteristics, wild *Populus* can be divided into 6 groups and 29 species, including section *Populus* (10), *Leucoides* (3), *Tacamahaaca* (9), *Aieiros* (3), *Turanga* (3) and *Abaso* (1)^2^. China has the largest number of poplar species, among which 62 poplar species are distributed in a wide geographical region of southwest, northwest, northeast, and north China. Except for section *Abaso*, other sections are distributed in different latitudes, elevations, and climatic ranges. Among these species, the poplars in the section *Leucoides* are tall and have straight white trunks, large heart-shaped leaves, and long-life spans. They are good trees for landscaping and building timber forests. *P. wilsonii* is an important species of section *Leucoides*. This species is endemic to China and mainly distributed in Shaanxi, Gansu, Hubei, Sichuan, Yunnan, and Xizang provinces. It grows in hillside forest with sea height of 1300–3300 meters, and has strong environmental adaptability, especially to drought, cold, and salinity.

In comparison with other woody plants, *Populus* has a modest genome size (450–550 Mb), easy vegetative propagation, rapid growth rate, high levels of genetic diversity, and relatively mature genetic transformation system, making it a model plant for forest genetic and breeding research^3,4^. Several high-quality poplar genomes have become available, including *P. trichocarpa* (Torr. and Gray)^3^, *P. euphratica* Oliv^5^, *P. alba*^6^ and *P. deltoides*^7^, which belong to section *Tacamahaca*, *Turanga*, *Populus* and *Aigeiros*, respectively. These four species and *P. wilsonii* grow in different environments and occur across diverse habitats. Unfortunately, these four species are not easily transformed, thus limiting their use in numerous molecular studies. In general, the genome of these *Populus* is used to obtain the gene sequences and spatio-temporal expression analysis, while genetic transformation experiments and phenotypic identification were carried out in hybrid poplar, such as poplar 84K^8^ and *P. tomentosa*^9^. However, considering the difference in genetic background, incomplete reproductive isolation is observed among different poplars, and heterologous transformation cannot fully analyze the function of some key genes^9–11^. *P. wilsonii* is only distributed in China and has a large number of special characters due to its special environmental factors. The whole gene sequencing of *P. wilsonii* will help us discover the key genes of its unique excellent traits, deepen our understanding of *Populus*, and play key roles in its genetic improvement. These genomic resources can not only facilitate the analysis of gene function of poplar, the molecular mechanism of wood formation, high biomass accumulation, and stress resistance traits but also contribute to the comparative genomics of different poplar species. Furthermore, the availability of multiple well-assembled *Populus* genomes is instrumental for functional genomics and molecular breeding and will greatly facilitate accurate inferences of phylogenomic and pan-genome analyses.

In this study, we used PacBio circular consensus sequencing (CCS) sequencing and Hi-C technology to construct a high-quality, chromosomal level genome of *P. wilsonii*. Furthermore, we constructed phylogenetic trees of main species of *Salicaceae* to reveal the evolutionary relationships among different species, and revealed the molecular mechanisms of the unique environmental adaptability of *P. wilsonii* through gene family analysis. In addition, at the genome-wide and gene family levels, combined with the gene expression patterns, we studied the metabolic pathways of *P. wilsonii* leaf shape regulation, cellulose, and lignin synthesis in depth and revealed the genetic mechanism of *P. wilsonii* leaf and wood development. This work provides a solid foundation for understanding the genetics of the extraordinary characteristics and evolutionary tracks of *Populus* species and contribute this resource to the scientific community.

## Results

### Genome sequencing and assembly

**Fig. 1:**
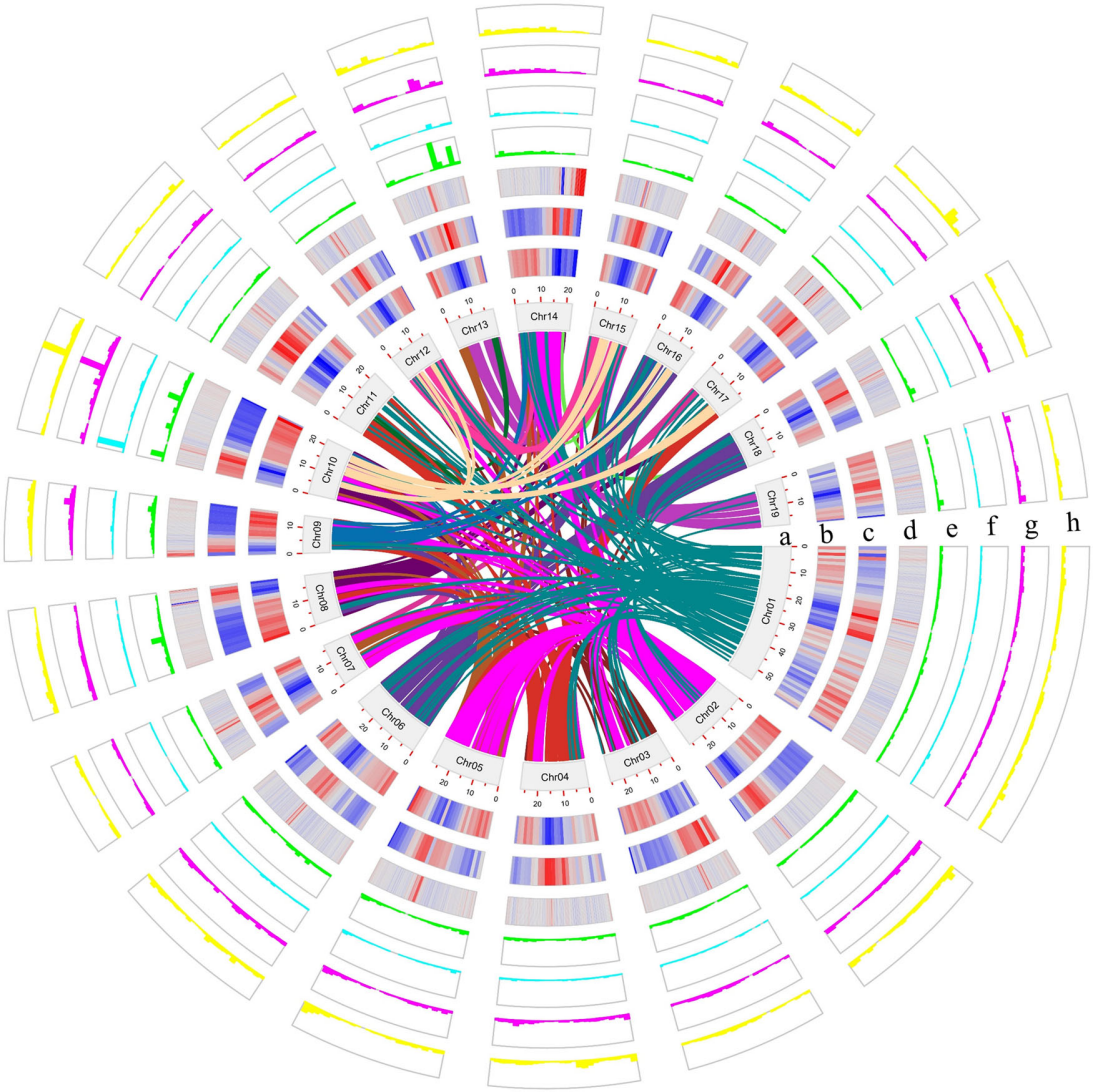
Overview of *Populus wilsonii* Genome. Elements are arranged in the following scheme (from inner to outer). **a** The 19 chromosomal pseudomolecules, units on the circumference are mega base values of pseudomolecules, (**b**) gene density, (**c**) repeat density, (**d**) GC content and gene expression levels in root (**e**), phloem (**f**), xylem (**g**) and leaf (**h**) are shown in 500 kb windows sliding 50 kb. Central colored lines represent syntenic links between the chromosomes.

To estimate the genome size and heterozygosity of *P. wilsonii* (Supplementary Fig. 1), we used 27.37 Gb of Illumina clean reads for K-mer analysis (Supplementary Tables 1 and 2). The results show that the number of modified 19-mers and the peak depth were 11,424 and 26, respectively (Supplementary Fig. 2, Supplementary Table 2). The estimated genome size and heterozygosity rate were calculated to be 439.39 Mb and 0.42%, respectively (Table 1 and Supplementary Table 2). By using the PacBio Sequel II platform, 33,348,682 PacBio clean subreads (~475.88 Gb) were generated from one cell, among which 2,019,886 CCS reads (~30.4 Gb) were collected with reads N50 of 15,075 bp and longest read of 36,256 bp (Supplementary Table 1). After the assembly of PacBio CCS reads, we obtained an initial genome with of 477.34 Mb in size, with a high contig N50 statistic value of 17.39 Mb and longest contig of 5445 Mb. To improve the quality of the genome assembly and anchor the contigs to chromosomes, we constructed high-throughput chromosome conformation capture (Hi-C) libraries of *P. wilsonii*, generating 181,253,818 (54.31 Gb) Hi-C pair-end reads (Supplementary Table 1). After duplicate removal, sorting. and quality assessment, the uniquely mapped valid reads were used for Hi-C scaffolding (Supplementary Table 3). As a result, 445.97 Mb (93.43%) of the assembly was placed on 19 chromosomes (Fig. 1a, Supplementary Fig. 3; Table 1). The sizes of contig N50 reached 16.3 Mb, in which the longest contig was 54.5 Mb, and the GC content was 35.58% (Fig. 1d; Table 1).

**Table 1:** Statistics for the *P. wilsonii* Genome and Gene Prediction.

Assembly feature	Number	Length	Percentage (%)
Estimated genome size		439.39 Mb	
Assembled scaffold sequences(>1 kb)	1161	477.35 Mb	
N50 scaffold		22.2 Mb	
N90 scaffold		14.67 Mb	
Max. scaffold		54.54 Mb	
Assembled contig sequences(>1 kb)	1287	477.34 Mb	
N50 contig		16.3 MB	
N90 contig		0.18 Mb	
Max. contig		54.45 Mb	
GC content			35.58
Gaps	126	12.6 kb	
Chromosome/Anchored scaffold	19/602	445.97 Mb	93.43
Anchored and oriented scaffold	145	429.87 Mb	90

Genome annotation	Number	Length	Percentage
Total repetitive sequence	708,246	238.47 Mb	49.95
Total genes	38,054	133.69 Mb	28
Genes in a chromosome	35,069	128.1 Mb	28.84
Noncoding RNAs	13,953	11.89 Mb	2.49
Pseudogenes	158	0.45 Mb	0.09

The completeness and accuracy of the *P. wilsonii* assembly were validated using BUSCO and CEGMA. BUSCO showed that 98.57% of the 1614 single-copy plant orthologues were complete, and CEGMA showed that the assembled genome completely covered 452 (98.69%) of the 458 core eukaryotic genes and 233 (93.95%) of the 248 highly conserved core eukaryotic genes (Supplementary Table 4). The draft assembly was further evaluated by mapping short and long high-quality reads to the genome assembly, resulting in a mapping rate of 99.63% and 99.94%, respectively (Supplementary Table 5). In addition, an average of 93% of the RNA sequencing (RNA-seq) data from different stages of stem and leaf and other tissues of *P. wilsonii* matched the genome assembly (Supplementary Table 6). These results indicate the high completeness, continuity, and base accuracy of the present genome assembly.

### Genome annotation

By using a combination of homology-based searches and *de novo* annotation, a total of 708,246 repeat elements were identified. These repeats were 238.47 Mb in size and comprised ~49.96% of the *P. wilsonii* genome assembly (Table 1 and Supplementary Table 7), and it consists of 25.65% retrotransposons, 12.56% DNA transposons, and 11.74% were TRs (Supplementary Table 7). Consistent with the pattern in other plants, LTR retrotransposons are the most abundant class of repetitive DNA, in which *Gypsy* repeats (12.90%) are the most abundant, followed by *Copia* (7.67%, Supplementary Table 7). These repeat elements were unevenly distributed along the chromosomes with a distinct preference for the centromeres (Fig. 1c).

Protein-coding gene models were predicted by combining homology, *de novo*, and RNA-Seq based methods. A total of 38,054 protein-coding genes and 158 pseudogenes were identified (Supplementary Fig. 4), among which 91.86% of genes supported by expressed sequence tags and homology-based searching with only 8.14% derived solely from *de novo* gene predictions (Supplementary Fig. 4). Of the all genes, 35,069 (92.16%) were assigned to a chromosomal location (Table 1). These genes were unevenly distributed along the chromosomes with a distinct preference for the ends (Fig. 1b), and these genes had different expression patterns in various tissues (Fig. 1e–h). The average gene length (3513 bp) and coding sequence length (1321 bp with 5.01 exons) were similar to those of other *Populus* species (Supplementary Table 8). For the completeness of protein-coding genes, 95.3% of the BUSCOs were found in the genome assembly (Supplementary Table 9), and 91.72% of transcriptome data were mapped to our predicted exons (Supplementary Fig. 5). Functional annotation confirmed that 98.67% (37,547 genes) of these predicted genes had known homologs in protein databases, and 98.26% and 98.37% of these genes exhibited homology and conserved protein domains in the TrEMBL and NCBI nr databases, respectively (Supplementary Table 10). We also annotated 8186 ribosomal RNAs (rRNAs), 5066 transfer RNAs (tRNAs), 126 micro RNAs (miRNAs), and 101 small nuclear RNAs (snRNAs) in *P. wilsonii* genome (Table 1, Supplementary Table 11).

### Phylogenetic evolution, whole-genome duplication and genome synteny

**Fig. 2:**
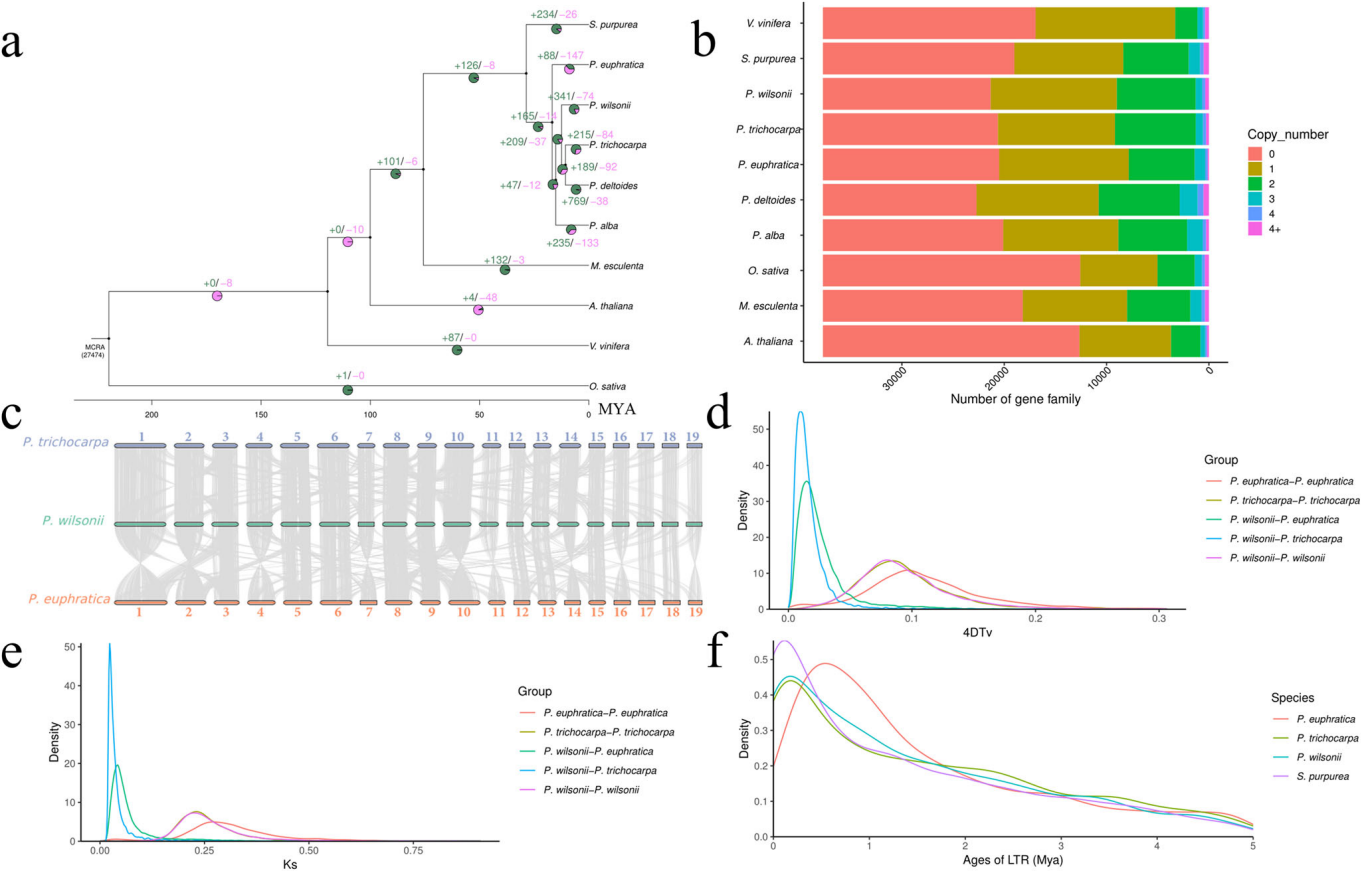
Evolution of *Populus wilsonii* and Relative Species in *Salicaceae*. **a** Phylogenetic tree of 9 species and *Populus wilsonii*. Gene family expansion and contraction compared with the most recent common ancestor. Gene family expansions/contractions are indicated in Cyan/purple. Inferred divergence times (MYA, million years ago) are denoted at each node. Venn diagram shows the ratio of gene family expansions and contractions. **b** Gene categories used from all the species (the resource of this figure is from Supplementary Data 5). **c** Syntenic blocks between *Populus wilsonii*, *P. trichocarpa* and *P. euphratica*. **d** Distribution of 4DTV values of syntenic orthologous genes in the genomes of *Populus wilsonii, P. trichocarpa* and *P. euphratica*. **e** Distribution of Ks values of syntenic orthologous genes in the genomes of *Populus wilsonii, P. trichocarpa* and *P. euphratica*. **f** Insertion time of LTRs in *Populus wilsonii, P. trichocarpa*, *P. euphratica* and *S. purourea*.

By using the available genome resources, a unique set of gene families among *P. wilsonii* and five Salicaceae species and among four outer species were identified (Supplementary Table 12). All species included in the analysis contained 37,723 gene families and shared 1049 single-copy and 1238 multiple-copy putative orthologous genes (Fig. 2b; Supplementary Table 13; Supplementary Data 5). A phylogenetic tree was constructed based on a concatenated sequence alignment of 1049 single-copy gene families shared in *P. wilsonii* and nine plant species and all the relationships were well supported with >99% bootstrap values. The results indicated that that *P. wilsonii* and four other *Populus* were grouped together (Fig. 2a). Moreover, five *Populus* originated in Neogene. The splice time of *P. wilsonii* and the ancestor of *P. deltoides* and *P. trichocarpa* was 12 (3–23) Mya, and the splice time of *P. euphratica* and *P. wilsonii*, *P. alba*, and *P. wilsonii* were 14 (4–28) Mya and 16 (5–31) Mya, respectively (Fig. 2a). This phylogenetic tree is consistent with the species relationships observed in previous studies^12^.

Intergenomic analysis revealed a different degree of linear relationships between the chromosomes of *P. wilsonii* genome, and 9592 collinearity gene pairs were identified (Fig. 1). The comparison of *P. wilsonii* genome with the genome of *P. trichocarpa*, *P. euphratica*, and *S. purourea* independently showed that all 19 chromosomes in *P. wilsonii* corresponded strongly to the 19 chromosomes of *P. trichocarpa*, *P. euphratica*, and *S. purourea*, except for slight gene flow among the 19 chromosomes (Fig. 2c and Supplementary Fig. 6). We identified 27,152, 26,258, and 24,361 collinear genes between *P. wilsonii* and *P. trichocarpa*, *P. euphratica*, and *S. purourea*, indicating that 74.64%, 70.34% and 66.58% of the *P. wilsonii* genome is colinear with these plants, respectively. The highly conserved collinearity supports the close evolutionary relationship among these species.

Collinearity analysis of inter- and intra-*Populus* genomes confirms the salicoid WGD event. Synonymous substitutions (ks) and four-fold synonymous third-codon transversion were characterized in *P. wilsonii*, *P. trichocarpa* and *P. euphratica*, and the sharp peak was ks 0.25 and 4DTV was 0.08, suggesting that the whole-genome duplication event was generated in *P. wilsonii*, corresponding to the salicoid WGD event (~65 Mya), indicating that the WGD event occurred after the divergence of Salicaceae and Euphorbiaceae (Fig. 2d, e). The distribution of ks and 4DTV values suggests that the divergence between *P. wilsonii* and *P. trichocarpa* occurred ~6.2 (5.2–7.3) Mya (ks, ~0.02; 4DTV, ~0.009), divergence between *P. wilsonii* and *P. euphratica* occurred at ~11.3 (10.4–12.2) Mya (ks, ~0.04; 4DTV, ~0.015), which was similar to the result of phylogenetic analysis (Fig. 2a). In addition, we modeled the age of LTRs in four Salicaceae species and found that the expansion of LTRs occurred earlier in *P. wilsonii* (~0.2 Mya) than in *P. euphratica* (~0.56 Mya), similar to *P. trichocarpa* (~0.2 Mya), but later than in *S. purourea* (~0.13 Mya, Fig. 2f). In general, LTR amplification and WGD event are involved in poplar speciation.

### Gene family analysis

**Fig. 3:**
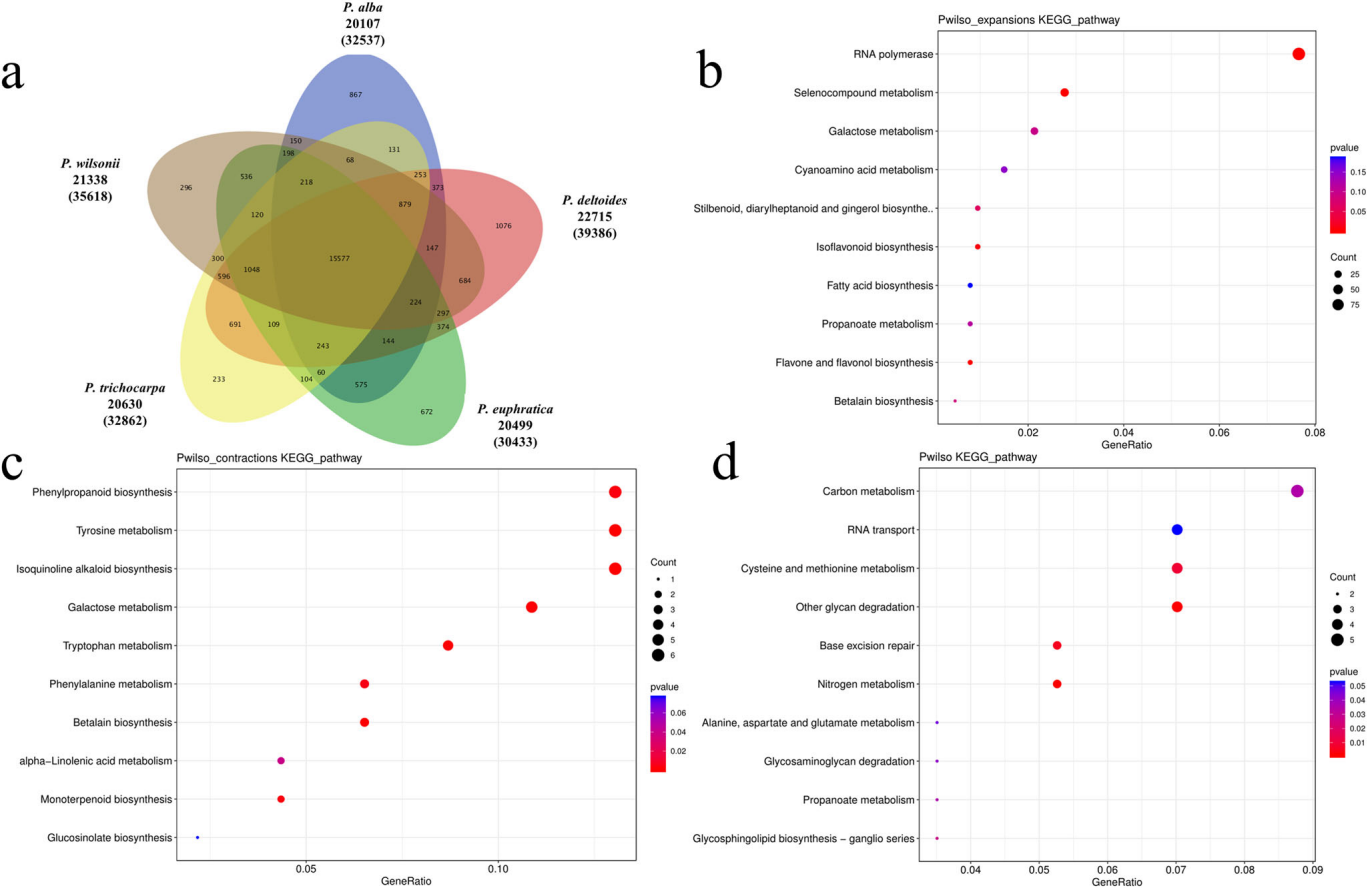
Gene family and functional enrichment analysis of *Populus wilsonii*. **a** The shared and unique gene families among five poplar species. KEGG enrichment analysis of expanded gene families (**b**), contracted gene families (**c**) and positively selected gene families (**d**) in *Populus wilsonii*.

Based on sequence homology, we assigned 35,618 genes from *P. wilsonii* genome to 21,338 families and 140 gene families, including 1072 genes, which were unique to this genome (Supplementary Fig. 7; Supplementary Table 13). Furthermore, four other *Populus* species were selected to identify unique and shared gene families. The results showed that 15,577 gene clusters were shared by the five species, and 296 specific gene families were identified in *P. wilsonii* genome (Fig. 3a). KEGG enrichment analysis revealed the unique genes of *P. wilsonii* genome were significantly enrichment (FDR < 0.05) in oxidative phosphorylation, ribosome, glucosinolate biosynthesis, and photosynthesis (Supplementary Fig. 8). And these genes were enriched mainly in ATP synthesis coupled proton transport, translation and cytokinin biosynthetic process by GO enrichment (Supplementary Fig. 9a).

The expansion and contraction of gene families is essential in adaptive phenotypic diversification^13^. We identified 341 and 74 gene families that show expansion or contraction, respectively, after divergence from *P. wilsonii* (Fig. 2a). KEGG analysis showed that the expanded gene families were involved in RNA polymerase, selenocompound metabolism, and flavone, flavanol, and isoflavonoid biosynthesis (Fig. 3b). Isoquinoline alkaloid biosynthesis, tyrosine metabolism, betalain biosynthesis, and tryptophan metabolism were uniquely enriched in the contracted gene families of *P. wilsonii* genome (Fig. 3c). GO analysis revealed the expanded gene families were mainly ascribed into signal transduction, photosystem II and ADP binding (Supplementary Fig. 9b); and the contraction gene families were mainly enriched in terms of lipid catabolic process, extracellular region and nucleoside metabolic process (Supplementary Fig. 9c).

In addition to the expansion and contraction of gene families, genes with positive selection commonly contributed to adaptive phenotypic evolution and adaptation. A total of 184 positively selected genes were identified in the *P. wilsonii* genome compared with four other *Populus* species. We performed a KEGG enrichment analysis of these positively selected genes and identified some significantly enriched KEGG pathways, which were related to nitrogen metabolism, other glycan degradation, base excision repair, and cysteine and methionine metabolism (Fig. 3d). And the terms of transport, cell wall and O − acetyltransferase activity were enriched by GO analysis with the positive selected genes (Supplementary Fig. 9d). These positively selected, unique, and expanded genes might contribute to the environmental adaptability of *P. wilsonii*.

### Transcriptome analysis of leaf and stem development

Transcriptomic sequencing was performed on the five development stages of leaf and stem in *P. wilsonii*. A total of 201.56 Gb clean data (5.72–8.1 Gb for each sample) were obtained, and an average of 93% of reads were mapped to the genome (Supplementary Table 6). Hence, we identified 2183 DEGs between L1 and L2, 6049 DEGs between L1 and L3, 7538 DEGs between L1 and L4, and 8245 DEGs between L1 and L5 in leaf development. We also identified 38 DEGs between S1 and S2, 172 DEGs between S1 and S3, 471 DEGs between S1 and S4, and 947 DEGs between S1 and S5 (Supplementary Fig. 10) in stem development. In general, the further apart the stages of leaf or stem development are, the more DEGs they have (Supplementary Fig. 10). A remarkable amount of 9698 and 1058 DEGs changed throughout the whole leaf and stem development stages, and 1791 and 16 common DEGs remarkably changed in leaves and stems, respectively (Supplementary Fig. 11). In addition, 806 and 124 TFs were identified in DEGs during leaf and stem development, respectively. Moreover, 59 TFs were shared during their development, including five GRF, seven WRKY, five bHLH, and four MYB (Supplementary Data 1).

KEGG enrichment analysis was carried out for all DEGs during the five development stages of leaves and stems, respectively. The results showed that 21 pathways were remarkably enriched during leaf development, such as plant hormone signal transduction, plant–pathogen interaction, starch and sucrose metabolism, carbon metabolism, and glycolysis/gluconeogenesis (Supplementary Fig. 12a), and six pathways were substantially enriched in stem development, including plant–pathogen interaction, flavonoid biosynthesis, pentose and glucuronate interconversions, and glutathione metabolism (Supplementary Fig. 12b). GO analysis showed the DEGs during leaf development were mainly involved in carbohydrate metabolic process and integral component of membrane (Supplementary Fig. 13a), and the DEGs during stem development mainly involved in cell wall organization and protein kinase activity (Supplementary Fig. 13b). For 1791 common DEGs in leaf development stages, carbon fixation in photosynthetic organisms, betalain biosynthesis, glyoxylate and dicarboxylate metabolism, carotenoid biosynthesis, and carbon metabolism were significantly enriched by KEGG analysis (Supplementary Fig. 12c), and microtubule-based movement and ATP/DNA binding were significantly enriched by GO analysis (Supplementary Fig. 13c). These pathways provided insights into the metabolic processes underlying different leaf and stem development stages in *P. wilsonii*.

### WGCNA

**Fig. 4:**
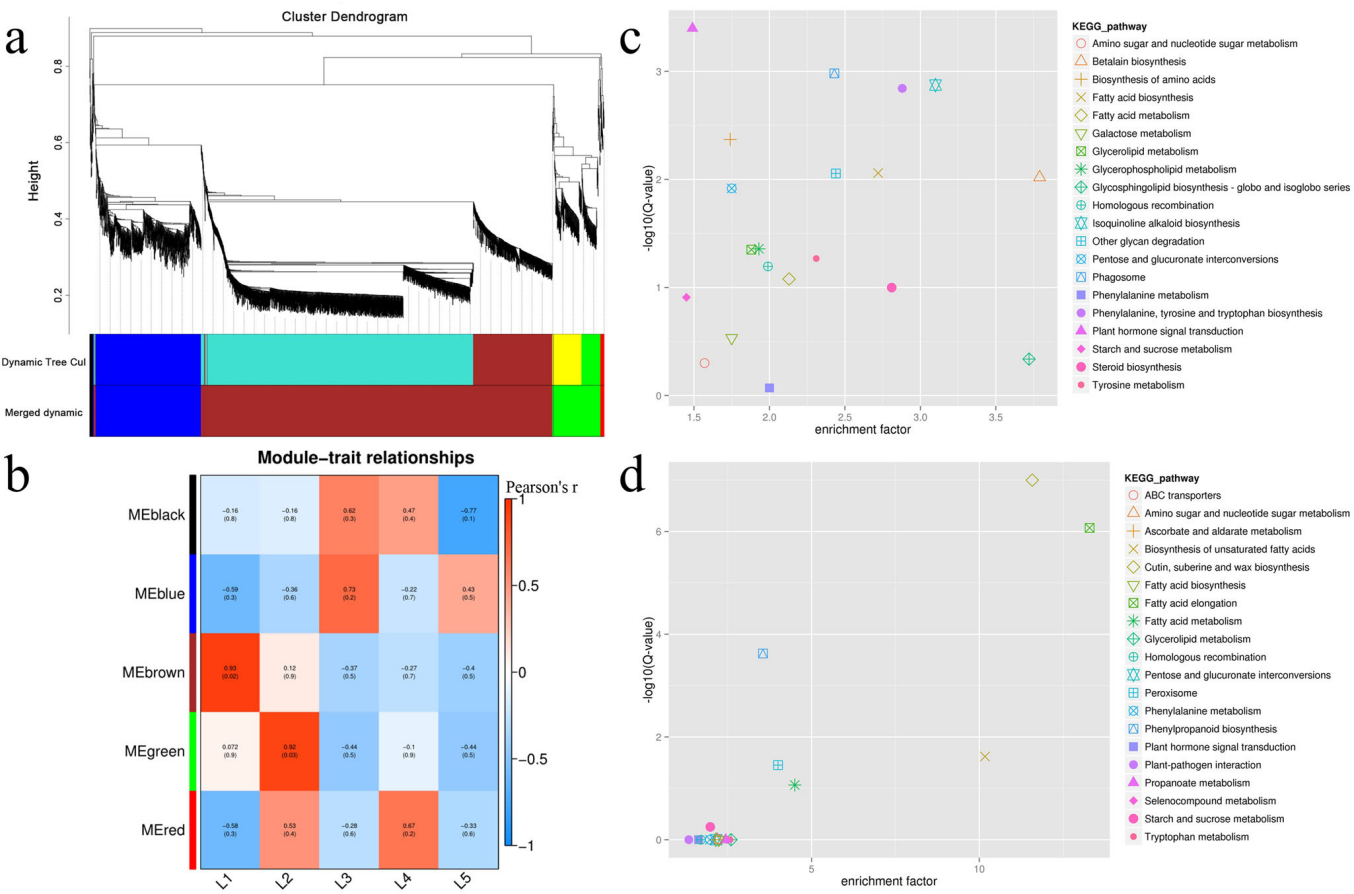
Weighted gene co-expression network analysis (WGCNA) of genes during the leaf development. **a** Hierarchical cluster tree showing co-expression modules identified by WGCNA. **b** Module-stage association (each row corresponds to a module, and each column represents a specific stage. The color of each cell at the row column intersection indicates the Pearson correlation coefficient (Pearson’s r) between a module and the stage), positive and negative correlations are shown in red and blue, respectively. **c** KEGG enrichment analysis of genes in brown module. **d** KEGG enrichment analysis of genes in green module. Each sample for WGCNA had three biological replicates.

WGCNA was performed to understand the biological process of leaf and stem development from the perspective of overall network (Fig. 4 and Supplementary Fig. 14). All identified genes were classified into five and three modules in leaf and stem development, respectively (Fig. 4a and Supplementary Fig. 14a), and some modules were highly correlated with the leaf and stem development stages (Fig. 4a, d). For example, during leaf development, brown module is related to stage L1 (*r* = 0.93), and green module is related to stage L2 (*r* = 0.93, Fig. 4b); during stem development, turquoise module is related to stage S1 (*r* = 0.98), and brown module is related to stage S5 with correlation coefficient of 1 (Supplementary Fig. 14b). Therefore, the genes in these modules can reflect the metabolic changes during the different development stages.

During leaf development, brown and green module were specific related to early leaf development stage (stages L1 and L2). KEGG enrichment analysis showed that genes involved in plant hormone signal transduction, biosynthesis of amino acids, fatty acid biosynthesis, glycan degradation and betalain biosynthesis pathways were the most enriched in the brown module (Fig. 4c). The terms of cutin, suberine and wax biosynthesis, phenylpropanoid biosynthesis, and unsaturated fatty acids biosynthesis were enriched in the green module (Fig. 4d). GO analysis showed that these genes in brown and green modules had same functions, which were mainly involved in extracellular region, catalytic activity and immune system process (Supplementary Fig. 15a, b). The results suggest that hormonal signals are essential to the early leaf development, and various metabolic pathways also medicate the process. During stem development, turquoise and brown module were specific related to early (stage S1) and late (stage S5) stem development stage, respectively. To compare the difference between the start and end stages of stem development, we performed KEGG analysis on turquoise and brown modules, and the result showed fatty acid elongation, pentose and glucuronate interconversions and flavonoid biosynthesis were enriched in the turquoise module (Supplementary Fig. 14c); and plant–pathogen interaction, cutin, suberine, and wax biosynthesis, and linoleic acid metabolism were enriched in the brown module (Supplementary Fig. 14d). GO analysis showed that these genes in turquoise and brown modules also had same functions, which were mainly involved in cell, binding and metabolic process (Supplementary Fig. 15c, d). This indicated that flavonoids highly accumulated at the early stage of stem development, which may protect stems from UV damage and insects, and function as herbivore deterrents and antimicrobial compounds. In addition, several secondary metabolites supply tolerance to biotic and abiotic stress in the late stage of stem development.

To further search for hub genes related to stage L1, L2, and S1, S5, we identified the top 50 hub genes with the highest internal connectivity, which measures the importance of genes among the modules. All the annotated genes are shown in Supplementary Data 2 and 3. In the green and brown module associated with leaf development, most genes encode enzymes such as *SERK1*, *GDSL*, *CEPR1*, *SAUR21*, and *ABC* transporters, which are involved in morphogenesis, leaf size, shape, and differentiation. Two bHLH TFs were identified with top rank, suggesting their critical regulation at the beginning of leaf development (Supplementary Data 2). In the turquoise and brown modules, which are associated with stem development, most genes encode enzymes and proteins such as *WRKY76*, *CML42*, *CCR4*, *ERF109*, and *LBD15*, which are necessary for secondary wall formation, stress responses, and plant–pathogen interaction. Notably, >20 TFs were identified in the top 50 rank, including seven WRKY, four C2H2 AP2/ERF, and one bHLH TFs, representing their essential roles in stem development (Supplementary Data 3). The expression of some genes in different modules were further confirmed by RT-qPCR (Supplementary Fig. 16).

### Analysis of transcription factors in stem and leaf development

In the present study, we identified 2,964 TFs among 94 families from the *P. wilsonii* genome, representing 7.8% of the protein-coding genes (Supplementary Data 4). Transcriptome analysis showed that many TFs, such as GRF, WRKY, and bHLH, were involved in leaf and stem development. The three TF families were identified with 18, 100, and 170 members, respectively. The number of members was similar to that of other species in the *Populous* genus (Supplementary Data 4). Evolutionary analysis divided them into different subgroups with similar motifs, conserved domains, and gene structures (Supplementary Figs. 17, 18, and 19). These members were evenly distributed on each chromosome and identified seven TRs. Five TRs were observed in the bHLH gene family distributed on chromosome 6, 12, 15, 18, and 19, while the WRKY gene family had two TRs located on chromosomes 13 and 18 (Supplementary Fig. 20). Tissue expression analysis found that *PwibHLH14/19/108/121/135* and *PwiWRKY45* are highly expressed both in leaves and stems, *PwibHLH1/28/41/48/60/63/72/111/131* is highly expressed in the leaves, and *PwibHLH17/29/32/50/62/74/84/102/107/144/136/142* and *PwiWRKY10/13/16/40/66/67/71/81/83* are highly expressed in the stems (Supplementary Figs. 21 and 22). *PwiGRF2/3/7/10/11/14/16* was highly expressed in the early leaf development, especially the L1 stage (Supplementary Fig. 22). RT-qPCR analysis showed the similar expression patterns of some TFs (Supplementary Fig. 23). These TRs and highly expressed genes may play an important role in regulating leaf and stem development in *P. wilsonii*.

### Analysis of cellulose and hemicellulose synthesis in *P. wilsonii*

The emergence of secondary walls is one of the most important evolutionary events in the adaptations of plants to terrestrial environments. Secondary walls, which are mainly composed of cellulose, lignin, and hemicellulose, play an important role in plant growth and development, and stress response. Cellulose is synthesized by plasma membrane-localized cellulose synthase (CesA) complexes. Except for xylan, all hemicellulose main chains are synthesized by cellulose synthase like (CSL) complexes. In addition, glycoside hydrolases (GH) and glycosyltransferase (GT) are also required for xylan synthesis. A total of 17 CesAs, 35 Csls, 30 GHs and 38 GTs were identified in the genome of *P. wilsonii*, and the number of genes is slightly smaller than in other poplars (Supplementary Table 14). These genes are mainly located on chromosome 1, 2, 6, and 14. CSL has four TR regions, and GH has one TR region (Supplementary Fig. 24). Tissue expression analysis found that most of the enzyme genes related to cellulose and hemicellulose synthesis, such as *PwiCesA10/1*4, *PwiCsL8/12/24*, *PwiGH2/9/16/17/23*, and *PwiGT8/9/21/31* (Supplementary Fig. 25), have higher expression levels in stems than in leaves, and some selected genes were further validated by RT-qPCR (Supplementary Fig. 26). Phylogenetic tree showed a 1:1 correspondence between CesAs in *P. wilsonii* and *P. trichocarpa*, and they were also divided into two groups with 12 and 5 members, respectively (Supplementary Fig. 27). Furthermore, *PwiCesA3/4/9/13/17* had higher expression in the late stem development stage, while the other CesAs had higher expression in the early stem development stage, which were consistent with the studies in *P. trichocarpa*^14^. Although these genes did not expand in the genome of *P. wilsonii*, these highly expressed genes and TRs may be key genes in the synthesis of cellulose and hemicellulose.

### Genetic basis of Flavonoid and Lignin biosynthesis in *P. wilsonii*

**Fig. 5:**
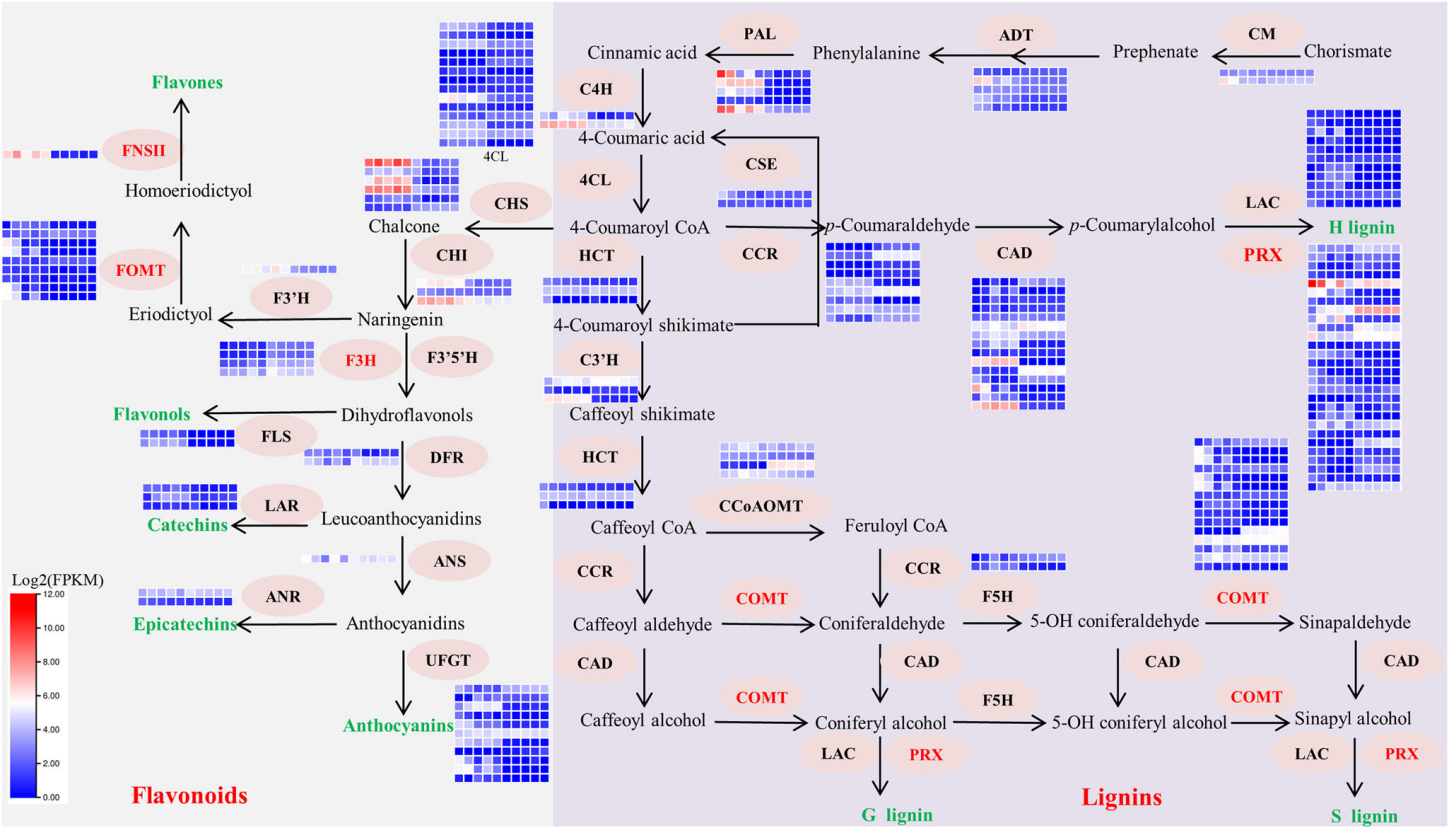
The Phenylpropane Metabolic Pathway Illustrates in flavonoids accumulation and lignin synthesis in *Populus wilsonii*. The lignin and flavonoid biosynthesis pathways have a common precursor. Expanded genes are shown in red. Enzyme abbreviations: CM chorismate mutase, ADT arogenate dehydratase, PAL phenylalanine ammonia-lyase, C4H cinnamate-4-hydroxylase, 4CL 4-coumarate CoA ligase, HCT quinate shikimate *p*-hydroxycinnamoyl transferase; C3H, 4-coumarate 3-hydroxylase; CCoAOMT, caffeoyl-CoA O-methyltransferase, COMT caffeic acid/5-hydroxyconiferaldehyde O-methyltransferase, CSE caffeoyl shikimate esterase, CCR cinnamoyl-CoA reductase, F5H ferulate 5‐hydroxylase, CAD cinnamyl alcohol dehydrogenase, LAC laccase, PRX peroxidase, CHS chalcone synthase, CHI chalcone isomerase, F3H flavanone 3-hydroxylase, F3′H flavonoid 3′-hydroxylase, F3′5′H flavonoid 3′5′-hydroxylase, DFR dihydroflavonol 4-reductase, ANR anthocyanidin reductase, ANS anthocyanidin synthase, FLS Flavonol synthase, FNSII Flavone synthase II, FOMT Flavonoid O-methyltransferase, ICS Isochorismate synthase, LAR Leucoanthocyanidin reductase, UFGT flavonoid 3-O-glucosyltransferase. Heatmap showing the expression level of candidate genes involved in flavonoids accumulation and lignin synthesis in different tissues of *Populus wilsonii*. The FPKM values are log2-based. Red and blue indicate high and low expression levels, respectively. Each tissue for heatmap for had three biological replicates.

Phenylpropanoids represent nearly 10,000 secondary metabolites derived from the phenylpropanoid pathway, and play key roles in plant response to biological and abiotic stress^15^. Among them, lignin and flavonoids biosynthesis pathways are the key branches of phenylpropane metabolism, and they share many intermediates and enzymes^16^. In general, five gene families including chorismate mutase (CM), arogenate dehydratase (ADT), phenylalanine ammonia-lyase (PAL), cinnamate-4-hydroxylase (C4H), and 4-coumarate: CoA ligase (4CL) are involved in the synthesis of precursors of flavonoids and lignin, and form the intermediate *p*-coumaroyl-CoA. These gene families have 38 members (3 CM, 5 ADT, 5 PAL, 3 C4H, and 22 4CL) in *P. wilsonii*, and each gene had high expression levels in stems and leaves (Fig. 5; Supplementary Table 15).

For lignin biosynthesis, eight lignin-specific enzyme gene families, including quinate shikimate *p*-hydroxycinnamoyl transferase (HCT), 4-coumarate 3-hydroxylase (C3H), caffeoyl CoA O-methyltransferase (CCoAOMT), caffeic acid/5-hydroxyconiferaldehyde O-methyltransferase (COMT), caffeoyl shikimate esterase (CSE), cinnamoyl-CoA reductase (CCR), ferulate 5‐hydroxylase (F5H), and cinnamyl alcohol dehydrogenase (CAD), catalyze the final conversion of *p*-coumaroyl-CoA to *p*-coumaryl alcohol (H monolignol), coniferyl alcohol (G monolignol), and sinapyl alcohol (S monolignol)^17^. Finally, these monolignols are polymerized by laccase (LAC) and peroxidase (PRX) to form lignin (Fig. 5). A total of 216 members of the ten enzymes gene families were identified in *P. wilsonii* genome, and they were slightly smaller than *P. trichocarpa* (229) but larger than *P. euphratica* (192, Supplementary Table 15). Interestingly, the number of lignin-specific genes slightly differed among different species of *Populus* (Supplementary Table 15), and many of them are mainly located on chromosome 1 and 2. Furthermore, a large number of TRs were observed in many gene families, such as CAD, PRX, LAC, and COMT (Supplementary Fig. 28). Notably, COMT and PRX gene families remarkably expanded in *P. wilsonii* genome, which may be helpful to improve the synthesis efficiency of lignin monomer^18^. Tissue expression and RT-qPCR analysis show that the expression of most genes in stems was higher than that in leaves, such as *PwiCOMT3/13/28*, *PwiPRX1/25/30/35/36/75/89*, *PwiCCR6/10*, *and PwiCAD14/19* (Fig. 5 and Supplementary Fig. 26), and this result is consistent with the accumulation of lignin in plant tissues. These genes, including TR genes and highly expressed genes, in stems are important in the synthesis of lignin in *P. wilsonii*.

Flavones, flavonols, catechins, epicatechins, and anthocyanins are the main constituent compounds of flavonoids. Chalcone synthase (CHS) and chalcone isomerase (CHI) are the common catalytic enzymes of all five compounds, and flavonoid 3′,5′-hydroxylase (F3′5′H) and flavanone 3-hydroxylase (F3H) are common catalytic enzymes, except for flavones. Dihydroflavonol 4-reductase (DFR) is the common catalytic enzyme of catechins, epicatechins, and anthocyaninsm, while anthocyanidin synthase (ANS) is the common catalytic enzyme of epicatechins and anthocyanins. Flavonoid 3′-hydroxylase (F3′H), flavonoid O-methyltransferase (FOMT), and flavone synthase II (FNSII) are the specific enzymes for flavones, flavonol synthase (FLS) is the specific enzyme for flavonols, leucoanthocyanidin reductase (LAR) is the specific enzyme for catechins, anthocyanidin reductase (ANR) is the specific enzyme for epicatechins, and flavonoid 3-O-glucosyltransferase (UFGT) is the specific enzyme for anthocyanins. In *P. wilsonii* genome, we identified 80 members from the 14 gene families, and their number was comparable to other *Populus* species (Supplementary Table 15). Location analysis showed that these genes were distributed throughout the genome but concentrated on chromosomes 1, 9, and 13, while 11 TRs were identified in FOMT, UFGT, CHS, and F3H gene families (Supplementary Fig. 29). Notably, FOMT, F3H, and FNSII gene families were significantly expanded compared with other *Populus* species. For example, three copies of F3H were observed in *P. wilsonii*, but only two copies were observed in *P. trichocarpa*. Tissue expression and RT-qPCR analysis showed that the expression level of most genes in leaves was higher than that in stems, such as *PwiCHS3/5/12*, *PwiFNSII3*, *PwiUFGT15/18/21*, and *PwiCHI1/3* (Fig. 5). The results correspond to the accumulation of flavonoids in stems and leaves, suggesting that the flavonoid pathway is active in leaves.

## Discussion

*P. wilsonii* is a unique poplar species in China, which plays an important role in soil and water conservation and garden ornamental. We assembled the 477.35 Mb chromosome-scale genome by integrating PacBio CCS and Hi-C technologies, with contig N50 of 16.3 Mb, scaffold N50 of 22.2 Mb, and 93.43% coverage of the full genome. The quality of this assembly was remarkably improved compared with the recently published poplar genome assemblies of *P. alba* (Contig N50 of 1.83 Mb)^6^, *P. euphratica* (Contig N50 of 40.44 Kb)^5^ and *P. simonii* (Contig N50 of 1.94 Mb)^19^ in terms of both continuity and gene annotation for *Populus* genus species. We also annotated 38,054 proteins in *P. wilsonii*, and the number of genes in *P. wilsonii* is greater than that in *P. trichocarpa*^3^ (34,699) and *P. euphratica*^5^ (36,426), but less than *P. deltoide*^7^ (44,853) and *P. alba*^6^ (40,213). Among these genes, 91.86% of which are supported by expressed sequence tags and homology-based searching. In conclusion, our sequencing and assembly strategies can obtain high-quality reference genomes. The divergence between *P. wilsonii* and *P. trichocarpa* was ~6.2 Mya, while that between *P. wilsonii* and *P. euphratica* was ~11.3 Mya, which was similar to the result of phylogenetic analysis^6,12^. Three poplar species including *P. wilsonii*, *P. euphratica* and *P. trichocarpa* had undergone two whole-genome duplications and they exhibited extensive collinearity across the gene space. The high-quality *P. wilsonii* genome sequence and transcriptome profiling data that we have completed and collected constituted important genetic, genomic and transcriptomic resources, which can be exploited in future research to understand the diversity, speciation, evolution and genetic basis of complex economic traits and adaption. It also provides important resources for exploring other species in the genus *Populus*, which have great economic, ecological and research value.

Wood is mainly composed of secondary walls, which are the mixture of cellulose, lignin, and hemicellulose^20^. Compared with *P. trichocarpa* and other *Populus* species, *P. wilsonii* expanded its COMT, CAD, PRX and LAC families. Moreover, most of these genes exhibited higher expression in stems than in leaves. COMT and CAD are the catalytic enzymes of the last two steps in the synthesis of lignin monomers. LAC and PRX are necessary for lignin polymerization during vascular development in plants, and responsible for the catalysis of monolignols^21^. The expansion of these cell wall-related genes in *P. wilsonii* genomes might contribute to the evolutionary changes of wood composition, thus further inducing the formation of harder and stronger wood. Furthermore, a relatively complete biosynthetic pathway for flavonoids was also constructed based on the synergistic analysis of genome sequencing, transcriptomics, and RT-qPCR analysis. FOMT and FNSII families are the specific enzymes for flavones and remarkably expanded in *P. wilsonii* genomes, which may promote the accumulation of flavones, further increasing the adaptations of *P. wilsonii* to terrestrial environments with expanded cell wall-related genes. The results were further confirmed by WCGNA.

TFs are important components in regulatory cascades during the leaf and stem development. In this study, we identified 2,964 TFs among 94 families based on comprehensive genome, transcriptome, and RT-qPCR. GRF, WRKY and bHLH TFs were highly expressed both in stems and leaves. GRFs play diverse roles in leaf size control and regulate secondary xylem development in numerous species^22,23^. 7 of GRFs were expressed preferentially in the L1 stage, indicating their key roles in leaf size. Moreover, bHLHs were also demonstrated to control leaf size and secondary wall formation^24,25^. Among 170 bHLH members, some tend to be expressed in stems, leaves, or both of them, suggesting their functional differentiation in controlling leaf size and secondary wall development. GRF and bHLH TFs should made outstanding contributions to the big-leaf characteristic of *P. wilsonii*. WRKYs are involved in plant growth, development, secondary wall biotic and abiotic stress combing with different TFs and genes^26,27^. The high expression of some WRKYs in stems may contribute to the wood quality of *P. wilsonii* through changing secondary wall composition, while the WRKYs expressed in leaves improve its resistance for various environmental factors. MYB, AP2/ERF, and LBD TFs were also identified in our studies and showed a association with stem and leaf development, which are in agreement with the studies in other plants^28–30^.

Additionally, unique physiologies of *P. wilsonii* may also necessitate complicated regulation by the unique genes, expanded genes, positively selected genes, tandem duplication, and DEGs in different development stages of leaf and stem. Meanwhile, some unique genes were enriched in the photosynthesis and supported the high photosynthetic rates of *P. wilsonii*. And most of the expanded gene families were enriched in the flavonoid biosynthesis, and high expressed in leaves or stems. The results further support the high accumulation of flavonoids and lignin in *P. wilsonii*. The positively selected genes of *P. wilsonii* which are enriched in some pathways responsible for biotic and abiotic stress, may supply strong evidence for its adaptations to alpine environments. The TRs of genes and TFs likely contributes to the biosynthesis of flavonoids and secondary wall components, such as WRKY, bHLH, PRX, COM, FOMT, and F3H, which are also found in the expanded genes and positively selected genes. We also used transcriptomic analysis to evaluate the expression profiles of DEGs and found most of the aforementioned genes are well coordinated with secondary wall and flavonoid biosynthesis and accumulation in *P. wilsonii*. Notably, some key genes and TFs were further validated by RT-qPCR analysis and selected as candidate genes for the utilization in molecular breeding.

In summary, the high-quality reference genome combined with transcriptome provide insights into the genome evolution and leaf and stem development of *P. wilsonii*. The contracted synthetic genes contribute to the accumulation of cellulose and hemicellulose in *P. wilsonii* compared with other poplars, while the expanded COMT, CAD, PRX and LAC families and their TRs play key roles in the lignin composition. Additionally, the expanded FOMT, F3H, and FOMT families are essential to the accumulation of *P. wilsonii* flavones. Meanwhile, the expanded GRF and bHLH TFs and their TRs play diverse roles in regulating leaf size and secondary wall formation of *P. wilsonii*, whereas the WRKY TFs provide supports for changing secondary wall composition and improving resistance for various environmental factors of *P. wilsonii*. Hence, the data from this study also offers economic, ecological and research resources for genetic studies and improvement of *P. wilsonii*, including genome-assisted breeding of novel cultivars with desired traits and exceptional economical fitness.

## Methods

### Plant materials, library construction and sequencing

For *de novo* genome sequencing, a single genotype of wild *P. wilsonii* (Supplementary Fig. 1) was collected from Donggouling, Nanzheng District, Hanzhong City, Shaanxi Province (32.87347 N, 106.61275 E) in April 2021. Total genomic DNA was isolated from fresh leaves of *P. wilsonii* by using the DNeasy Plant Mini Kit (Qiagen) and purified from the gel using a QIAquick Gel Extraction kit (Qiagen). A total of 10 ug high molecular weight DNA was sent to Biomarker Technologies Corporation (Beijing, China), and part of it was used to construct circular consensus sequencing (CCS) libraries and sequence them using a PacBio Sequal II platform, and other portions were used for library preparation with insert sizes of 350 bp and sequenced them using the Illumina NovaSeq 6000 platform.

For Hi-C sequencing, fresh tissues of *P. wilsonii* were crosslinked with formaldehyde at room temperature for 30 min. Nuclear extraction and DNA samples were digested with restriction endonuclease HindIII. Then, sticky end repair and free blunt ends were ligated. After ligation, DNA was purified and sheared to 300–700-bp fragments. The DNA fragments were used for the construction of Hi-C library and paired- end sequencing with a length of 150 bp was conducted on the Illumina NovaSeq 6000 platform. After removing the adapter and primer sequences and filtering low-quality data, 54.30 Gb clean data were obtained.

In addition, total RNA was extracted from seven tissues (e.g., root, xylem, phloem, leaves, leaf buds, flower buds, and flowers) of *P. wilsonii* by using a RNeasy plant mini kit (Qiagen), and 3 µg of RNA per tissue was used for library preparation. Each sample had three biological replicates.

### Genome assembly and quality assessment

The genome size of *P. wilsonii* was estimated based on K-mer (*k* = 19) analysis^31^ by using the quality-filtered reads, which were sequenced on the Illumina platform. The genome size and heterozygosity rate were estimated using a homemade script.

The *P. wilsonii* genome was assembled as follows: First, we assembled contigs from CCS clean reads by using Hifiasm (V 0.12)^32^ with default parameters. Second, the clean Hi-C reads, were directly truncated at the putative Hi-C junctions and then the resulting trimmed reads were aligned to the assembly results with bwa aligner. Only uniquely alignable pairs reads whose mapping quality >20 were retained for further analysis. Invalid read pairs, including Dangling-End and Self-cycle, Re-ligation and Dumped products, were filtered by HiC-Prov2.8.1. Before chromosomes assembly, we first performed a preassembly for error correction of contigs which required the splitting of contigs into segments of 50 kb on average. The Hi-C data were mapped to these segments using BWA (v 0.7.10-r789) software^33^. The uniquely mapped data were retained to perform assembly by using LACHESIS^34^. Any two segments which showed inconsistent connection with information from the raw scaffold were checked manually. These corrected scaffolds were divided into subgroups and sorted and oriented into pseudomolecules by using LACHESIS^34^ with the following parameters: CLUSTER MIN RE SITES = 28, CLUSTER MAX LINK DENSITY = 2, ORDER MIN N RES IN TRUNK = 6, and ORDER MIN N RES IN SHREDS = 7. After this step, placement and orientation errors exhibiting obvious discrete chromatin interaction patterns were manually adjusted.

The accuracy of Hi-C assembly was evaluated using several methods. We first inspected the Hi-C contact heatmap, and an elevated link frequency was observed with a diagonal pattern within individual pseudochromosomes, indicating increased interaction contacts between adjacent regions (Supplementary Fig. 3). Additionally, core eukaryotic gene dataset, including BUSCO (v4) and CEGMA (v2.5), were selected to assess the completeness of the genome. Finally, BWA^33^ and Minimap2^35^ were used to compare the second-generation and third-generation data with the draft genomes to assess their coverage.

### Annotation of repetitive sequences

To identify transposable elements, we used the RepeatModeler2 (v2.0.1)^36^ to identify *de novo* repeat types in *P. wilsonii*. LTR_retriever (v2.8)^37^ was performed to ab initio long terminal repeat (LTR) transposon element prediction. Then, based on the combined results from RepeatModeler with the databases Repbase(v19.06), REXdb(V3.0) and Dfam (v3.2) were used as the final repeat sequence libraries for subsequent analysis with RepeatMasker (v4.1.0)^38^. MISA (v2.1)^39^ and TRF (v. 4.07b)^40^ were also used to identify tandem repeats (TRs).

The flanking sequences on both sides of LTR were extracted and compared by MAFFT (v7.205), and the distance was calculated using Kimura model in EMBOSS^41^. The insert time was estimated using the formula *t* = *K*/2*r*, where the molecular clock *r* was set to 7 × 10^−9^ which come from the Arabidopsis study^42^.

### Gene prediction

All repetitive regions except TRs were soft-masked for protein-coding gene annotation. Protein-coding genes were predicted using *de novo* gene, homology-based, and RNA-seq-based prediction. The *de novo* gene models were predicted using Augustus (v3.0.3)^43^ and SNAP^44^. For homology-based prediction, protein sequences from *Arabidopsis thaliana* (Araport11, phytozome) and three closely related species including *P. trichocarpa*^3^, *P. deltoides* (V2.1, phytozome), and *P. euphratica*^5^ were aligned to the genome assembly of *P. wilsonii* using GeMoMa (v1.7)^45^ with the default parameters. For RNA-seq-based prediction, clean RNA-seq reads from seven tissues were aligned to the reference genome by using HISAT2^46^ and assembled using StringTie^47^. GeneMarkS-T(v5.1)^48^ was used to predict genes based on the assembled transcripts. Gene models from these different approaches were combined using EVM software (v1.1.1)^49^ to generate a consensus gene set. The rRNAs, tRNAs, and ncRNA were also predicted in the *P. wilsonii* genome.

Gene function prediction was inferred according to the best match of the alignments to the NCBI nr, EggNOG, KOG, TrEMBL, and Swiss-Prot protein databases by using diamond BlastP and the KEGG database with an *E* value threshold of 1E-5. The protein domains were annotated using InterProScan (v5.34)^50^ based on the InterPro protein database. The motifs, domains, and gene models were identified using the PFAM database. Gene ontology (GO) IDs for each gene were obtained from TrEMBL, InterPro, and EggNOG.

### Phylogenetic tree construction and evolution rate estimation

To cluster families from protein-coding genes, we used proteins from the longest transcripts of each gene from *P. wilsonii* and nine other related representative species (Supplementary Table 12). Orthologous gene clusters in 10 plants were identified using the OrthoFinder (v2.4.0)^51^ software. To construct the phylogenetic tree, single-copy orthologous genes were used; each gene family nucleotide sequence was aligned using MAFFT (v7.205) program, and the alignments were curated with GBLOCKS (v0.91b). Then, the alignment (four-fold degenerate positions) was used to compute the tree and infer the divergence dates with the optimal model and 2000 bootstrap replicates using IQ-TREE (v1.6.11)^52^. Finally, MCMCTREE in PAML (version 4.9e) was used to estimate the divergence times and 95% confidence intervals (CIs) of *P. wilsonii* and other plants^53^. Three fossil calibration times were obtained from the TimeTree database (http://www.timetree.org), including the divergence times of *Oryza sativa* versus *P. euphratica* (115–308 million years ago, MYA), *Vitis vinifera* versus *Manihot esculent*a (107–135 MYA), and *P. euphratica* versus *S. purpurea* (11–47 MYA).

### Gene family analysis

Based on the identified gene families and constructed phylogenetic tree with predicted divergence time of those species, we used CAFE^54^ to analyze gene family expansion and contraction. In CAFE, a random birth and death model was proposed to study gene gain or loss in gene families across a specified phylogenetic tree. Then, conditional *p*-value was calculated for each gene family, and the family with conditional *p*-value of <0.05 was considered to have an accelerated rate for gene gain or loss.

Based on the phylogenetic tree, we selected five closest species, including five *Populus*, to estimate the rate ratio (*ω*) of non-synonymous (Ka) to synonymous (Ks) nucleotide substitutions by using PAML (v4.9e) package^53^ to examine the selective constraints. The likelihood ratio test (LRT) of *p*-values was used to further verify the significant genes under positive selection.

### Whole-genome duplication and synteny analysis

The Ks (synonymous mutation rate) and 4DTv (four-fold synonymous third-codon transversion rate) methods are commonly used to identify whole-genome duplication events (WGDs). Here, the wgd (v1.1.0) software and custom script (https://github.com/JinfengChen/Scripts) were used to identify WGD events in *P. wilsonii*. The syntenic blocks between different *Populus* and *Salix purpurea* were identified using MCScanX^55^ with default parameters. Protein was used as queries in searches against the genomes of other plant species to determine the best matching pairs. Each aligned block represents an orthologous pair derived from the common ancestor.

### Transcriptome sequencing and RT-qPCR

Samples from five development stages of leaf (2nd, 4th, 6th, 8th, and 10th internode leaf corresponding to L1—L5) and stem (the annual, biennial, triennial, 4-year-old, and 5-year-old stems corresponding to S1, S2, S3, S4, and S5) immediately frozen in liquid nitrogen following harvesting. Each sample had three biological replicates. RNA isolation, library construction, sequencing, and read filtering were same as the RNA-seq for gene prediction analysis.

HISAT2^46^ was used to map the clean reads to the genome with the default parameters. Transcripts were assembled using StringTie^47^. Gene expression was measured as fragments per kilobase of transcript per million fragments (FPKM) mapped by using StringTie^47^, while the differentially expressed genes (DEGs) were determined using DEseq2^56^. Genes with significant differences in expression, fold change ≥ 2, and adjusted *P*-value < 0.05, were considered DEGs, and then annotated to GO terms and KEGG pathways.

Some of the genes or transcription factors (TFs) were selected for RT-qPCR analysis. The first-strand cDNA was synthesized using the NovoScript® Plus all-in-one first Strand cDNA synthesis SuperMix (gDNA Purge, Novoprotein, Shanghai, China), and the gene-specific primers are listed in Supplementary Table 16.

### Weighted gene co-expression network analysis (WGCNA)

Co-expression networks in leaves and stems were constructed using the WGCNA^57^ package in R. Soft thresholds were set based on the scale-free topology criterion. An adjacency matrix was developed using squared Euclidean distance values. The topological overlap matrix was calculated for unsigned network detection by using Pearson method. Co-expression coefficients >0.7 between the target genes were then selected.

### Functional genes analysis

To detect known TFs in the *P. wilsonii* genome, we used the iTAK programme^58^. The predicted gene sets were then used as queries in searches against the database. To investigate the genes involved in the cellulose and phenylpropane biosynthesis pathways in *P. wilsonii* genome, the genes were identified by BLASTP based on *Populus trichocarpa* related proteins with the criteria of similarity >80% and coverage >80%. We then confirmed the presence of the conserved domains within all protein sequences and removed members without a complete domain.

### Statistics and reproducibility

All data comprised at least three biological replicates. Statistical analysis among different groups for pairwise comparisons was performed using one-way ANOVA followed by Dunnett’s test (*p* < 0.05).

### Reporting summary

Further information on research design is available in the Nature Research Reporting Summary linked to this article.

## Supplementary information

supplementary information

Description of Additional Supplementary Files

Supplementary Data 1–5

reporting summary

## Data Availability

The final assembly genome, whole-genome sequence data (including Illumina reads, PacBio reads and Hi-C interaction reads), transcriptomes data of different tissues used to assist genome annotation and transcriptomes data of different tissues at different developmental stages in this study have been deposited in the Genome Sequence Archive in the BIG Data Center, Beijing Institute of Genomics (BIG), Chinese Academy of Sciences, under project number PRJCA008004. The final assembly genome and other data were also deposited in figshare (https://figshare.com/articles/dataset/Populus_wilsonii_genome_data/19143284) which is publicly accessible at this website. Source data underlying Fig. 2b are presented in Supplementary Data 5.
