# Supplementary material for: Chromosome-scale genome assembly provides insights into the molecular mechanisms of tissue development of Populus wilsonii
Source: Commun Biol. 2022 Oct 25;5:1125. doi: 10.1038/s42003-022-04106-0 (PMC9596445; doi:10.1038/s42003-022-04106-0)
Supplement: Supplementary file 1 — supplementary information [file 42003_2022_4106_MOESM1_ESM.pdf]

Chromosome-Scale Genome Assembly Provides Insights into the Molecular Mechanisms of Tissue Development of *Populus wilsonii*

Chaofeng Li[1*], Haitao Xing[2*], Can Li[3], Yun Ren[2], Honglei Li[2], Xue-Qin Wan[4], Chunlan Lian[5], Jia-Xuan Mi[4], Shengkui Zhang[3]

[1]Maize Research Institute, Southwest University, Chongqing, 400715, PR China

[2]College of Landscape Architecture and life Science/Institute of special Plants, Chongqing University of Arts and Sciences, Chongqing 402168, PR China

[3]School of Bioengineering, Qilu University of Technology, Jinan 250353, Shandong, PR China

[4]College of Forestry, Sichuan Agricultural University, Chengdu 611130, PR China

[5]Asian Research Center for Bioresource and Environmental Sciences, Graduate School of Agricultural and Life Sciences, The University of Tokyo, 1-1-1 Midori-cho, Nishitokyo, Tokyo 188-0002, Japan

Chaofeng Li and Haitao Xing contributed equally to this work.

Corresponding author: Shengkui Zhang Email: zsk8920@gmail.com

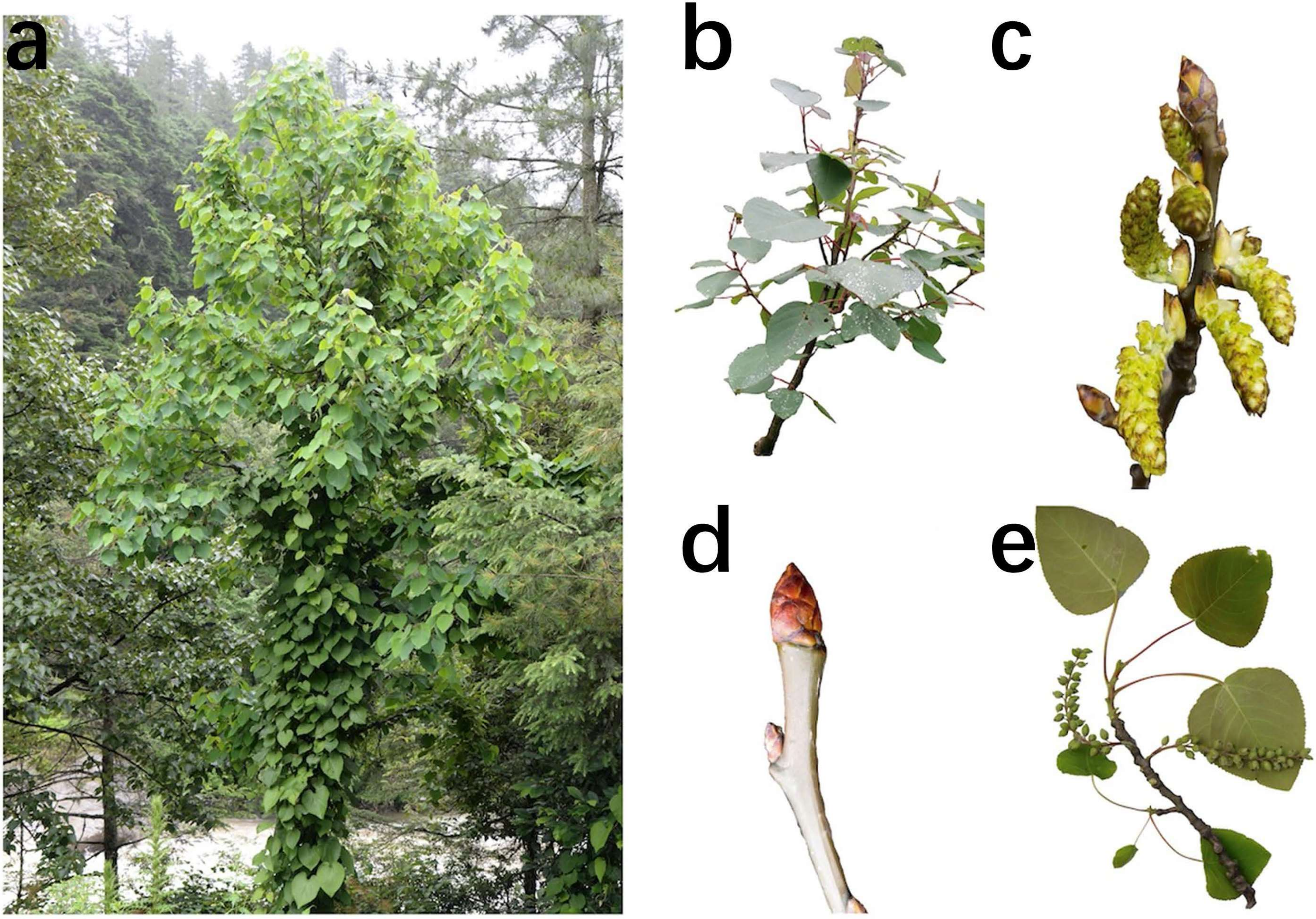
a
b
c
d
e

Supplementary Figure 1. Phenotype of the reference genome accession, "*Populus wilsonii*". (a) *Populus wilsonii* in their natural habitat. (b) Branches. (c) Male flowers. (d) Leaf bud. (e) Female flowers.

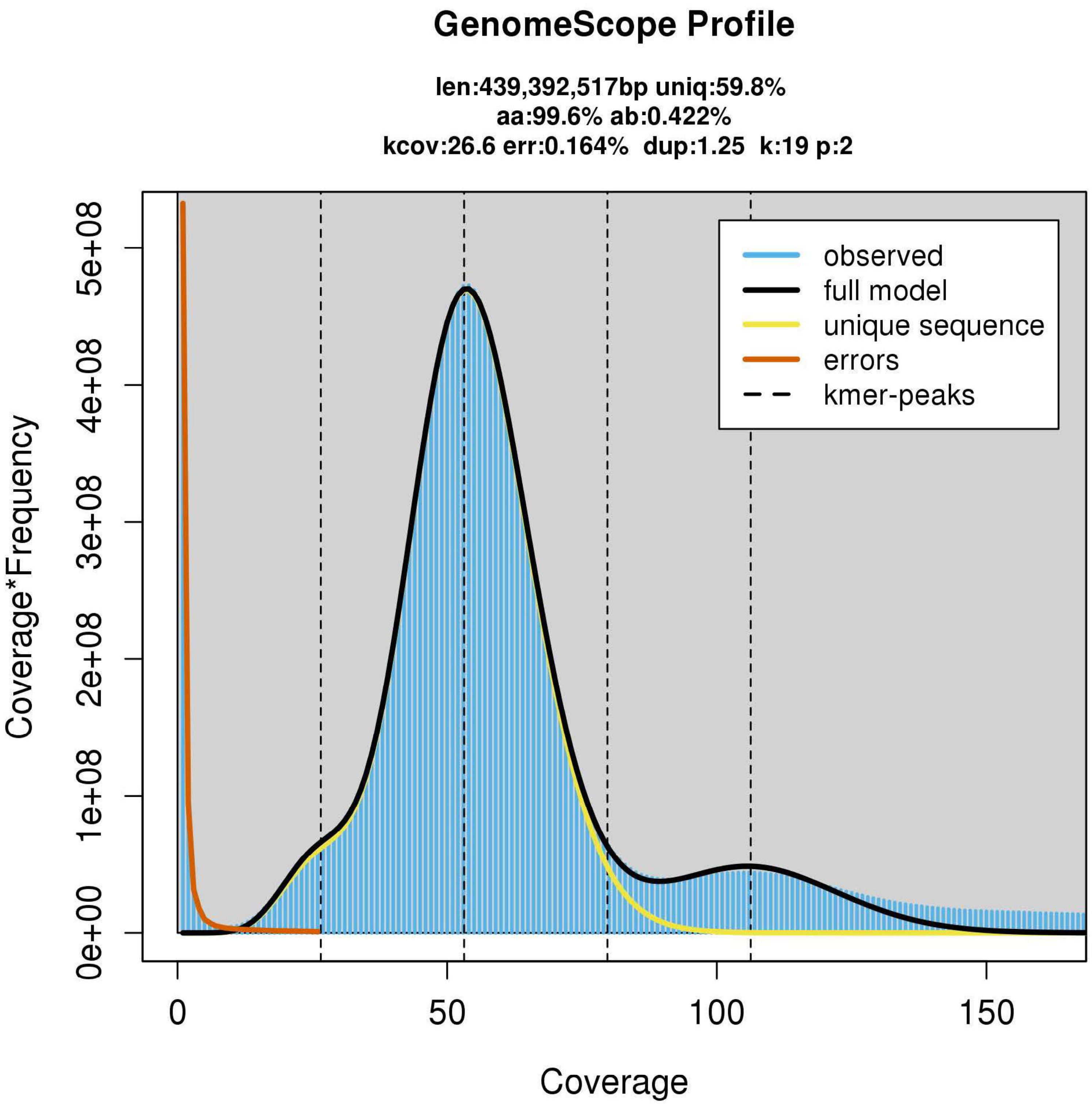
GenomeScope Profile
len:439,392,517bp uniq:59.8%
aa:99.6% ab:0.422%
kcov:26.6 err:0.164% dup:1.25 k:19 p:2
observed
full model
unique sequence
errors
kmer-peaks
Coverage*Frequency
0e+00
1e+08
2e+08
3e+08
4e+08
5e+08
0
50
100
150
Coverage

Supplementary Figure 2. The size estimation of the *Populus wilsonii* genome. The genome size was estimated by calculating the distribution of 19-mer frequency in the sequencing reads. The x-axis is the depth (X), and the y-axis is the proportion of sequences that represent the frequency at that depth divided by the total frequency of all depths.

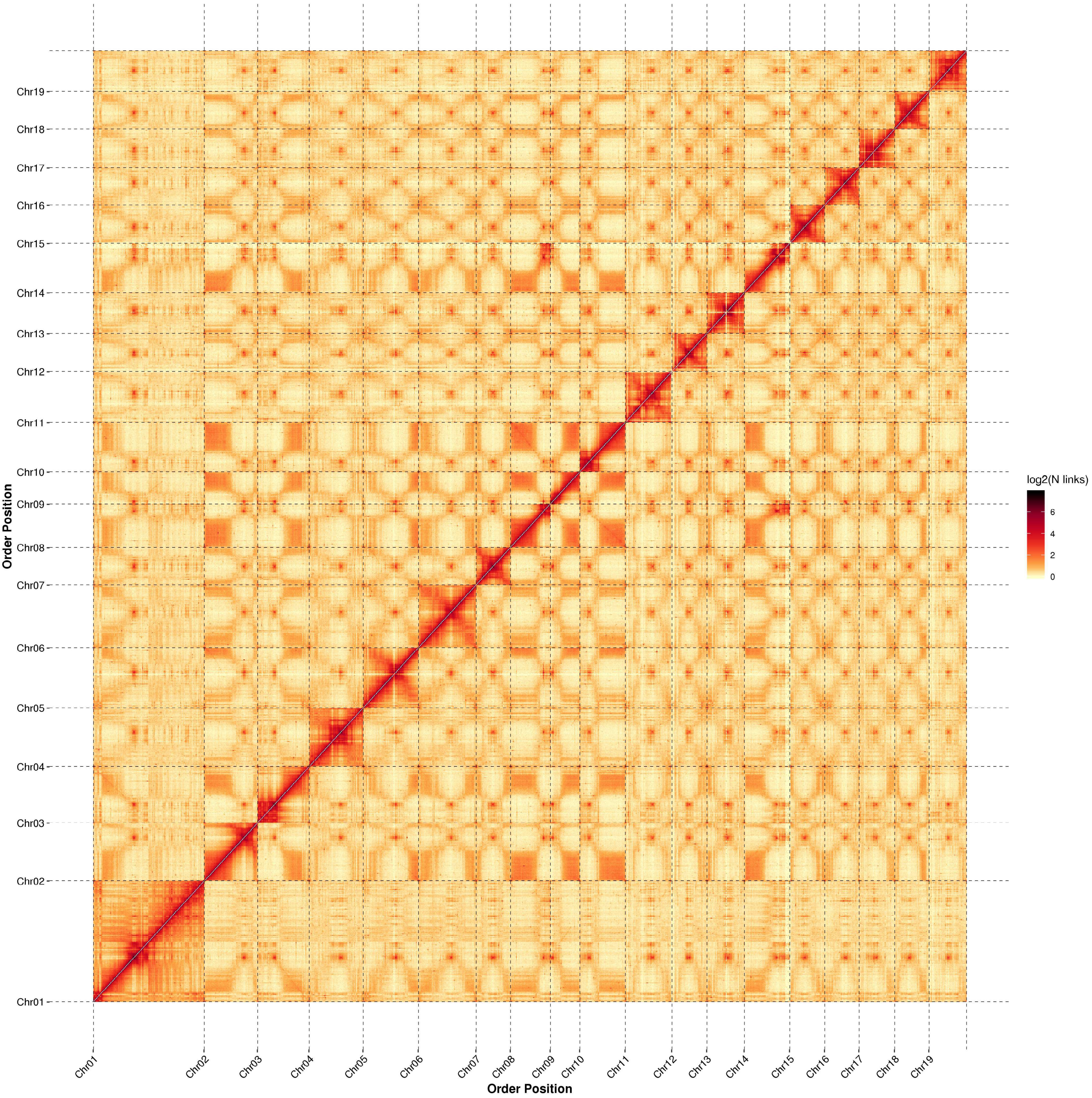

Chr01
Chr02
Chr03
Chr04
Chr05
Chr06
Chr07
Chr08
Chr09
Chr10
Chr11
Chr12
Chr13
Chr14
Chr15
Chr16
Chr17
Chr18
Chr19
Order Position
Order Position
log2(N links)
6
4
2
0

Supplementary Figure 3. The Hi-C chromatin interaction map for the 19 pseudomolecules of *Populus wilsonii* genome.

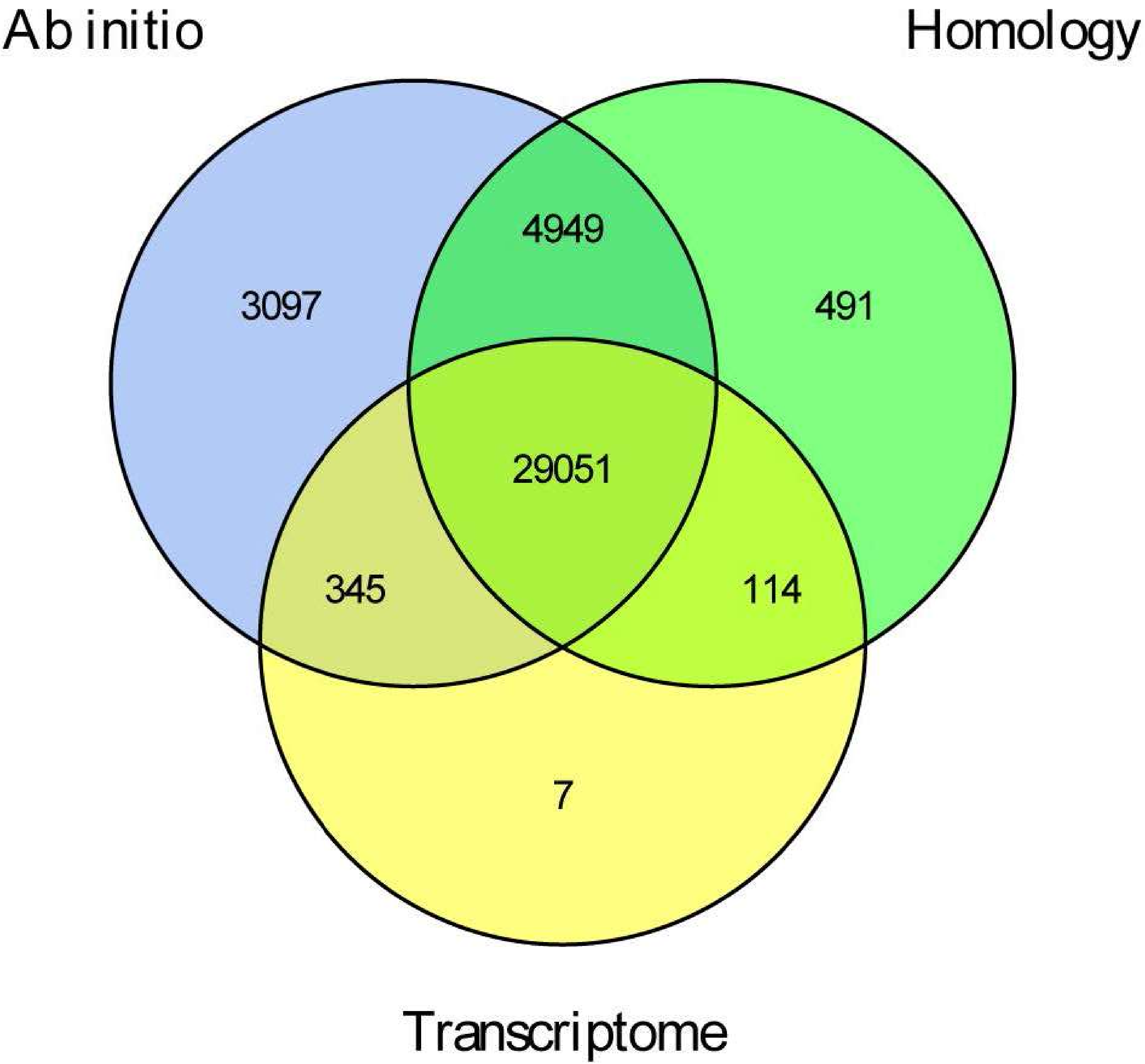
Ab initio
Homology
4949
3097
491
29051
345
114
7
Transcriptome

Supplementary Figure 4. The number of protein-coding gene models predicted by the homology-based, de novo and RNA-Seq based methods.

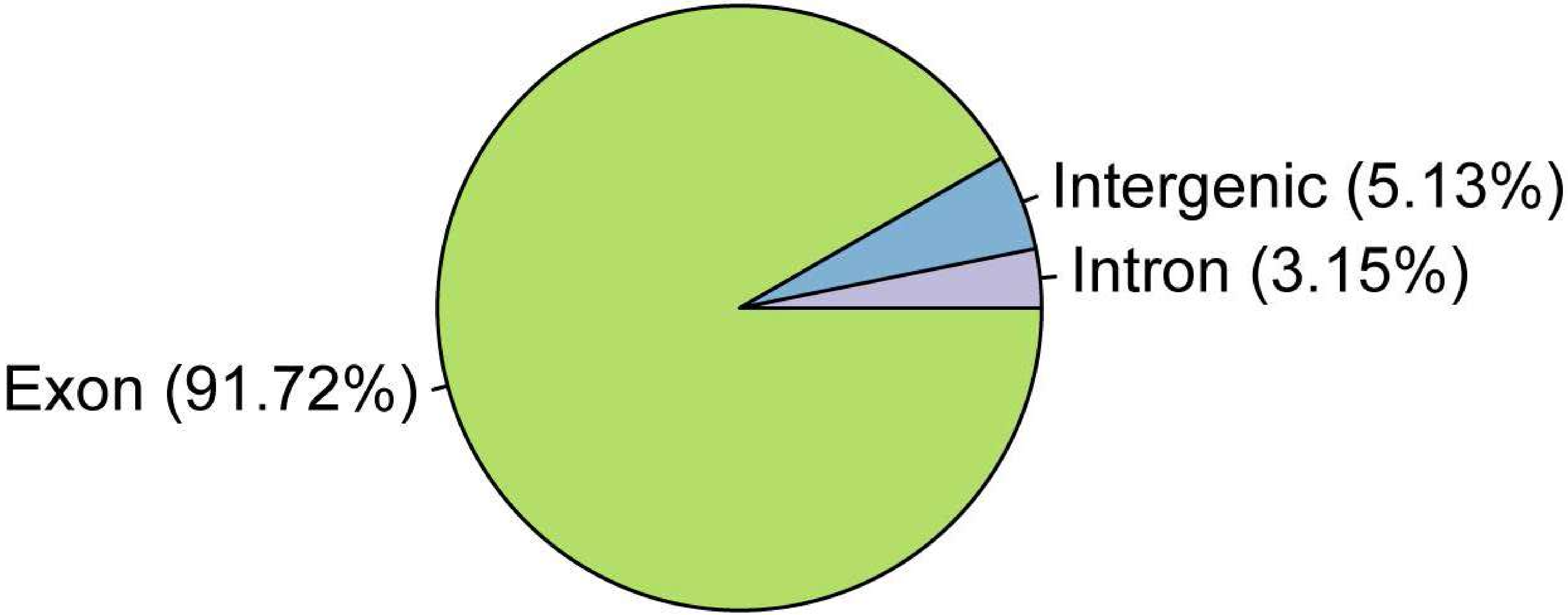
Intergenic (5.13%)
Intron (3.15%)
Exon (91.72%)

Supplementary Figure 5. Statistical results of RNA-seq clean data map to *Populus wilsonii* genome.

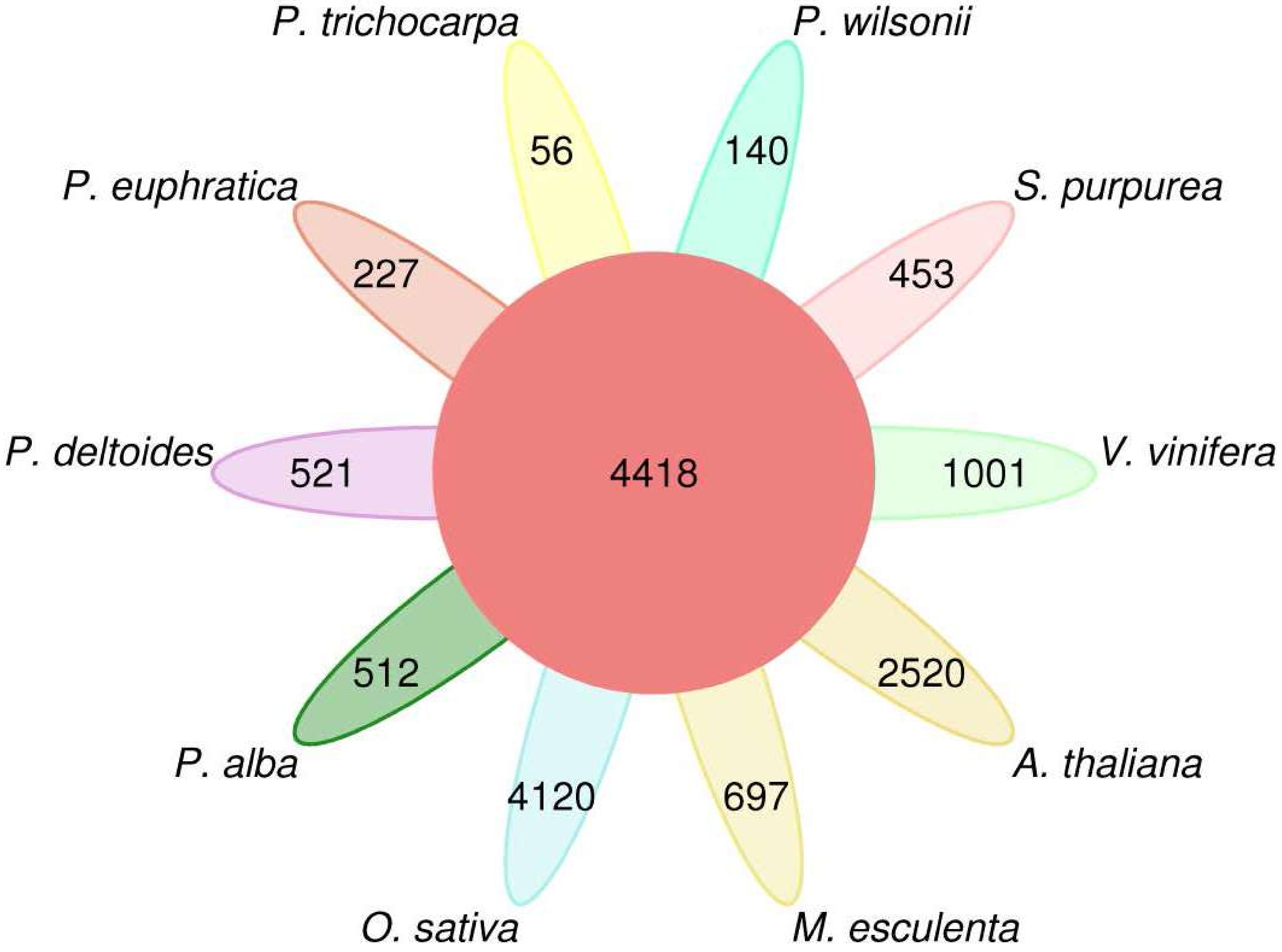

P. trichocarpa
56
P. wilsonii
140
P. euphratica
227
S. purpurea
453
P. deltoides
521
4418
V. vinifera
1001
512
2520
P. alba
A. thaliana
4120
697
O. sativa
M. esculenta

Supplementary Figure 6. Syntenic blocks between *Populus wilsonii* and *Salix purourea*.

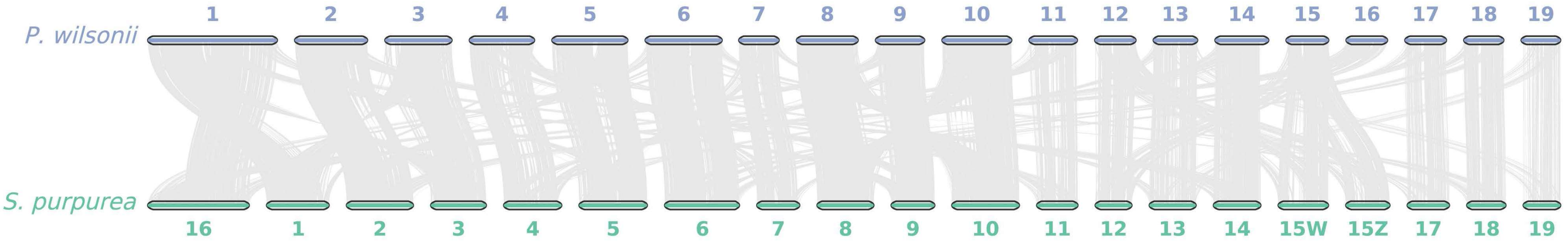
P. wilsonii
1
2
3
4
5
6
7
8
9
10
11
12
13
14
15
16
17
18
19
S. purpurea
16
1
2
3
4
5
6
7
8
9
10
11
12
13
14
15W
15Z
17
18
19

Supplementary Figure 7. The number of gene families shared among all species shown in Venn diagrams.

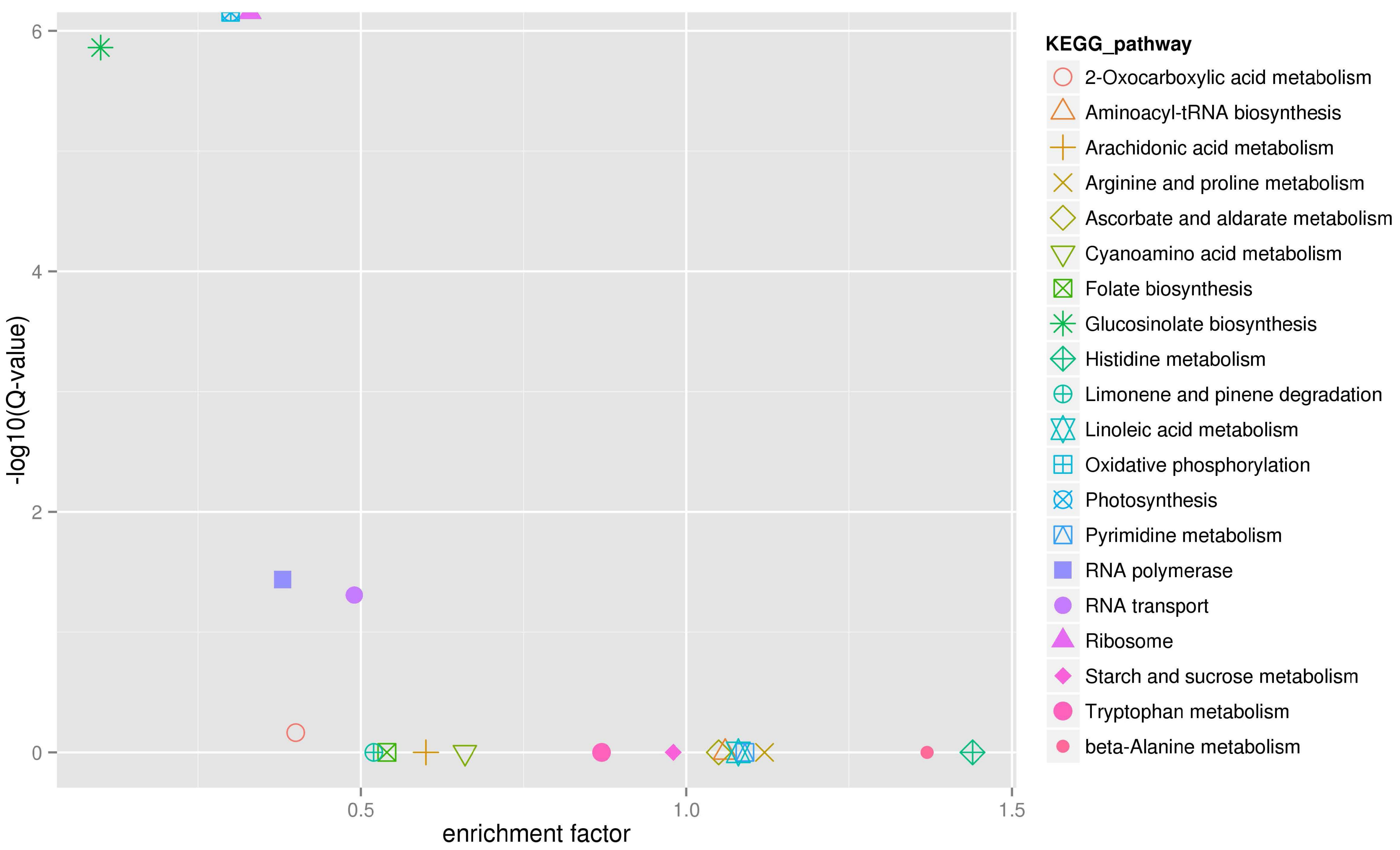
KEGG_pathway
2-Oxocarboxylic acid metabolism
Aminoacyl-tRNA biosynthesis
Arachidonic acid metabolism
Arginine and proline metabolism
Ascorbate and aldarate metabolism
Cyanoamino acid metabolism
Folate biosynthesis
Glucosinolate biosynthesis
Histidine metabolism
Limonene and pinene degradation
Linoleic acid metabolism
Oxidative phosphorylation
Photosynthesis
Pyrimidine metabolism
RNA polymerase
RNA transport
Ribosome
Starch and sucrose metabolism
Tryptophan metabolism
beta-Alanine metabolism
-log10(Q-value)
enrichment factor
0
2
4
6
0.5
1.0
1.5

Supplementary Figure 8. KEGG enrichment analysis of unique gene families in *Populus wilsonii*.

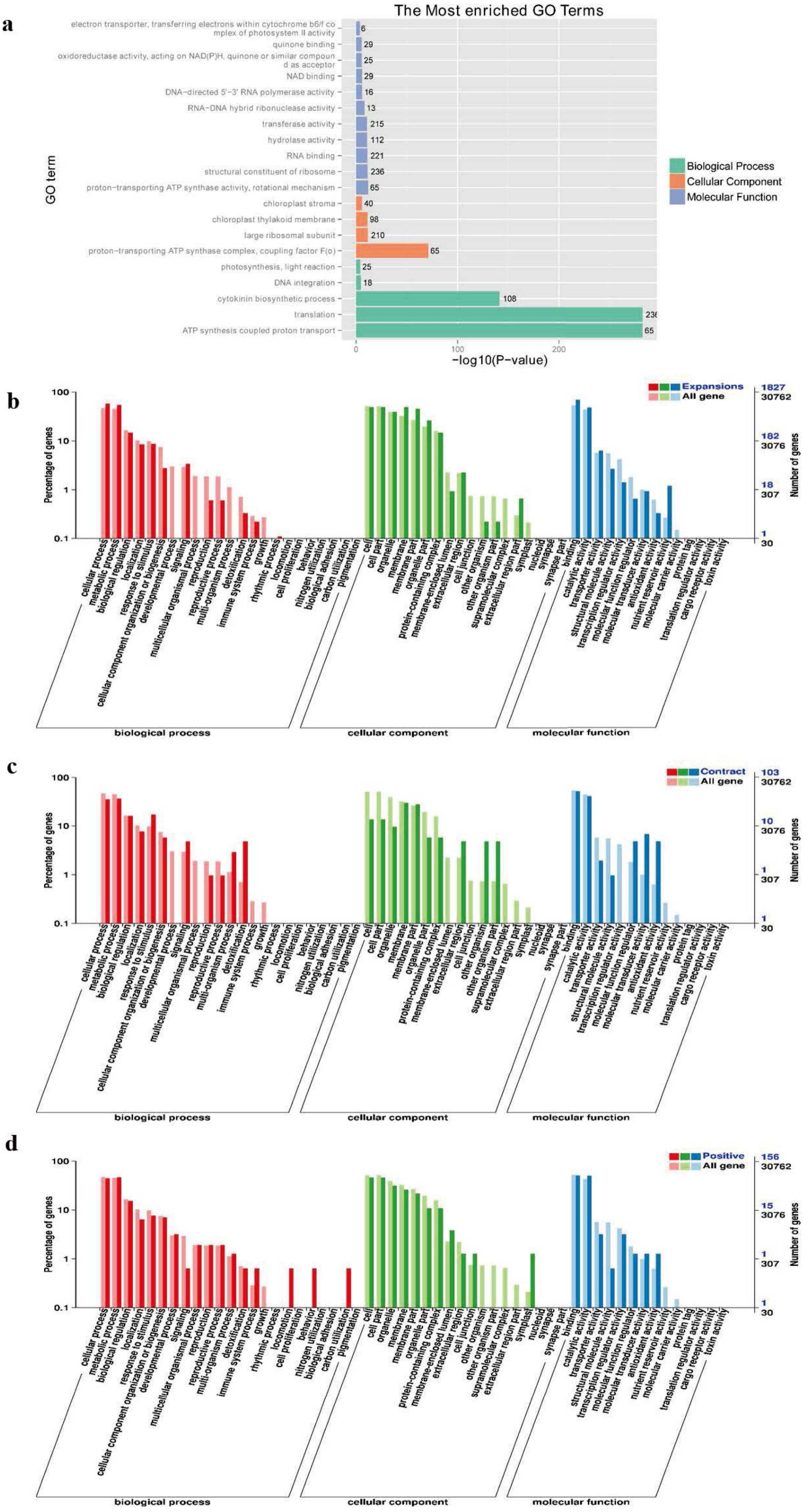

a
The Most enriched GO Terms
GO term
electron transporter, transferring electrons within cytochrome b6/f co mplex of photosystem II activity
quinone binding
oxidoreductase activity, acting on NAD(P)H, quinone or similar compoun d as acceptor
NAD binding
DNA-directed 5'-3' RNA polymerase activity
RNA-DNA hybrid ribonuclease activity
transferase activity
hydrolase activity
RNA binding
structural constituent of ribosome
proton-transporting ATP synthase activity, rotational mechanism
chloroplast stroma
chloroplast thylakoid membrane
large ribosomal subunit
proton-transporting ATP synthase complex, coupling factor F(o)
photosynthesis, light reaction
DNA integration
cytokinin biosynthetic process
translation
ATP synthesis coupled proton transport
6
29
25
29
16
13
215
112
221
236
65
40
98
210
65
25
18
108
236
65
0
100
200
-log10(P-value)
Biological Process
Cellular Component
Molecular Function
b
Expansions
All gene
Percentage of genes
Number of genes
100
10
1
0.1
1827
30762
182
3076
18
307
1
30
cellular process
metabolic process
biological regulation
localization
response to stimulus
cellular component organization or biogenesis
developmental process
multicellular organismal process
signaling
reproduction
reproductive process
multi-organism process
detoxification
immune system process
growth
rhythmic process
locomotion
cell proliferation
behavior
nitrogen utilization
biological adhesion
carbon utilization
pigmentation
cell
cell part
organelle
membrane
membrane part
organelle part
protein-containing complex
membrane-enclosed lumen
extracellular region
cell junction
other organism
other organism part
supramolecular complex
extracellular region part
symplast
nucleoid
synapse
synapse part
binding
catalytic activity
transporter activity
structural molecule activity
transcription regulator activity
molecular function regulator
molecular transducer activity
antioxidant activity
nutrient reservoir activity
molecular carrier activity
protein tag
translation regulator activity
cargo receptor activity
toxin activity
biological process
cellular component
molecular function
c
Contract
All gene
Percentage of genes
Number of genes
100
10
1
0.1
103
30762
10
3076
1
307
1
30
biological process
cellular component
molecular function
d
Positive
All gene
Percentage of genes
Number of genes
100
10
1
0.1
156
30762
15
3076
1
307
1
30
biological process
cellular component
molecular function

Supplementary Figure 9. GO enrichment analysis of unique gene families(a), expansion gene families(b), contraction gene families(c) and positive selected gene families(d) in *Populus wilsonii*.

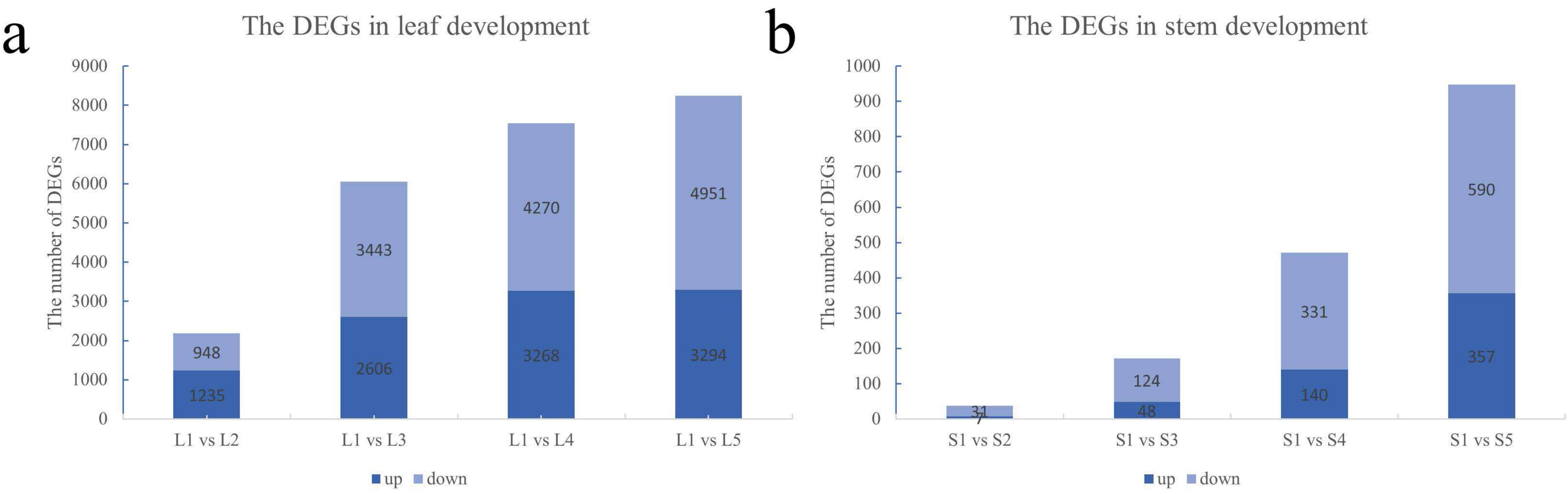
a
The DEGs in leaf development
The number of DEGs
9000
8000
7000
6000
5000
4000
3000
2000
1000
0
948
1235
3443
2606
4270
3268
4951
3294
L1 vs L2
L1 vs L3
L1 vs L4
L1 vs L5
up
down
b
The DEGs in stem development
The number of DEGs
1000
900
800
700
600
500
400
300
200
100
0
31
7
124
48
331
140
590
357
S1 vs S2
S1 vs S3
S1 vs S4
S1 vs S5
up
down

Supplementary Figure 10. The DEGs in leaf(a) and stem (b) development.

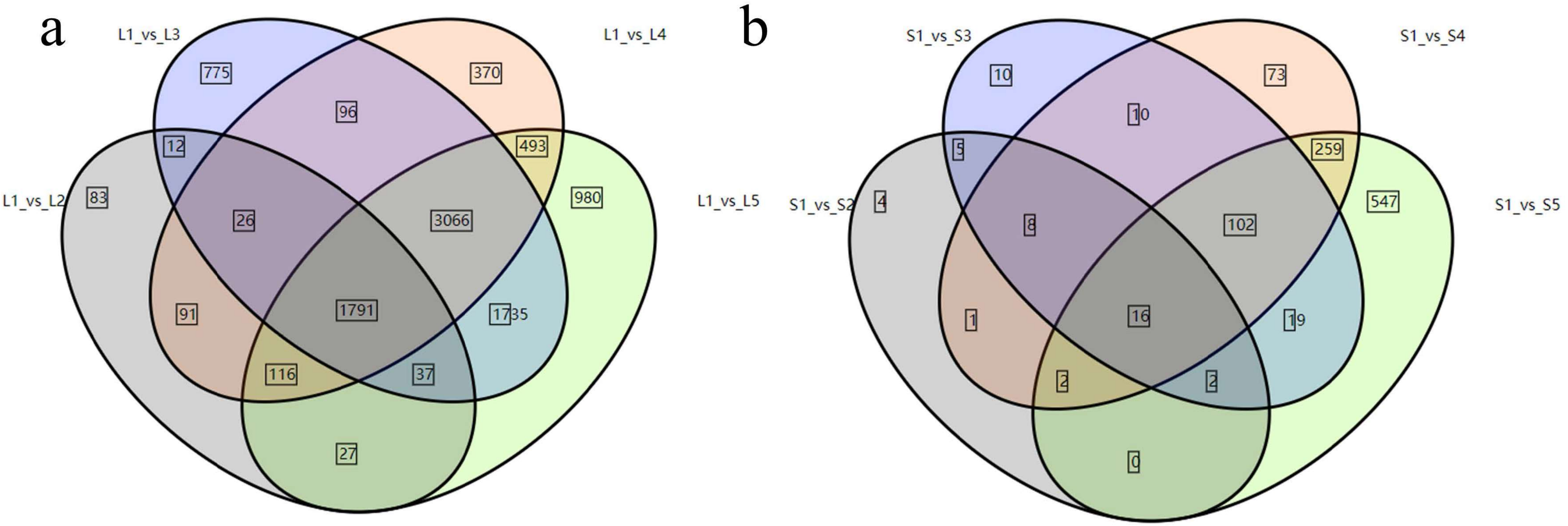
a
L1_vs_L3
L1_vs_L4
L1_vs_L2
L1_vs_L5
775
370
96
12
493
83
980
26
3066
91
1791
1735
116
37
27
b
S1_vs_S3
S1_vs_S4
S1_vs_S2
S1_vs_S5
10
73
10
5
259
4
547
8
102
1
16
19
2
2
0

Supplementary Figure 11. The shared and unique DEGs between the five adjacent stages in leaf(a) and stem (b) development.

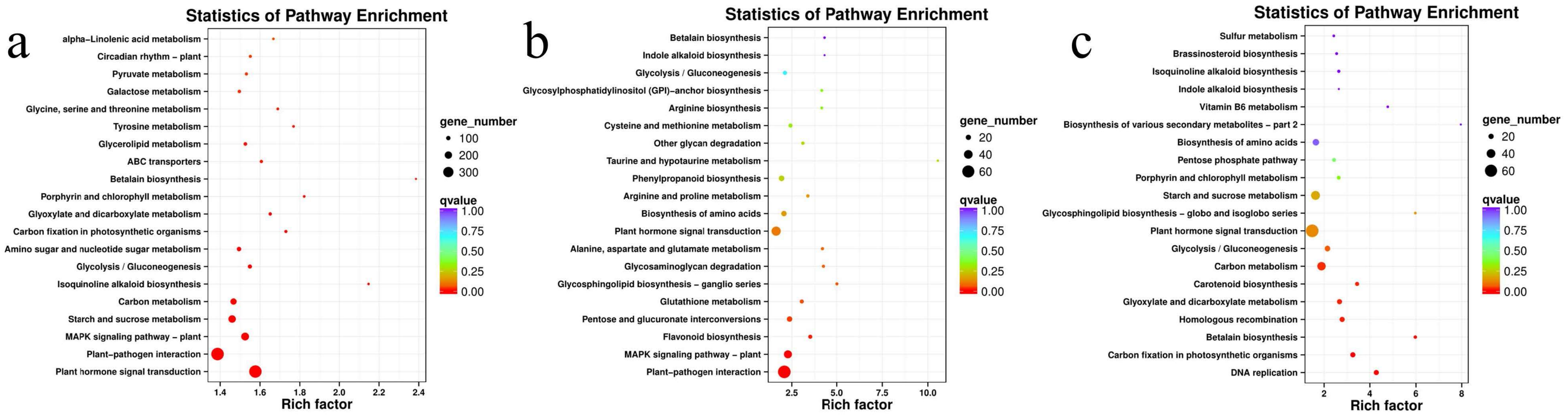

a
Statistics of Pathway Enrichment
alpha−Linolenic acid metabolism
Circadian rhythm − plant
Pyruvate metabolism
Galactose metabolism
Glycine, serine and threonine metabolism
Tyrosine metabolism
Glycerolipid metabolism
ABC transporters
Betalain biosynthesis
Porphyrin and chlorophyll metabolism
Glyoxylate and dicarboxylate metabolism
Carbon fixation in photosynthetic organisms
Amino sugar and nucleotide sugar metabolism
Glycolysis / Gluconeogenesis
Isoquinoline alkaloid biosynthesis
Carbon metabolism
Starch and sucrose metabolism
MAPK signaling pathway − plant
Plant−pathogen interaction
Plant hormone signal transduction
1.4 1.6 1.8 2.0 2.2 2.4
Rich factor
gene_number
100
200
300
qvalue
1.00
0.75
0.50
0.25
0.00
b
Statistics of Pathway Enrichment
Betalain biosynthesis
Indole alkaloid biosynthesis
Glycolysis / Gluconeogenesis
Glycosylphosphatidylinositol (GPI)−anchor biosynthesis
Arginine biosynthesis
Cysteine and methionine metabolism
Other glycan degradation
Taurine and hypotaurine metabolism
Phenylpropanoid biosynthesis
Arginine and proline metabolism
Biosynthesis of amino acids
Plant hormone signal transduction
Alanine, aspartate and glutamate metabolism
Glycosaminoglycan degradation
Glycosphingolipid biosynthesis − ganglio series
Glutathione metabolism
Pentose and glucuronate interconversions
Flavonoid biosynthesis
MAPK signaling pathway − plant
Plant−pathogen interaction
2.5 5.0 7.5 10.0
Rich factor
gene_number
20
40
60
qvalue
1.00
0.75
0.50
0.25
0.00
c
Statistics of Pathway Enrichment
Sulfur metabolism
Brassinosteroid biosynthesis
Isoquinoline alkaloid biosynthesis
Indole alkaloid biosynthesis
Vitamin B6 metabolism
Biosynthesis of various secondary metabolites − part 2
Biosynthesis of amino acids
Pentose phosphate pathway
Porphyrin and chlorophyll metabolism
Starch and sucrose metabolism
Glycosphingolipid biosynthesis − globo and isoglobo series
Plant hormone signal transduction
Glycolysis / Gluconeogenesis
Carbon metabolism
Carotenoid biosynthesis
Glyoxylate and dicarboxylate metabolism
Homologous recombination
Betalain biosynthesis
Carbon fixation in photosynthetic organisms
DNA replication
2 4 6 8
Rich factor
gene_number
20
40
60
qvalue
1.00
0.75
0.50
0.25
0.00

Supplementary Figure 12. KEGG enrichment analysis of all DEGs in leaf(a) and stem(b) development, and shared DEGs in leaf development(c).

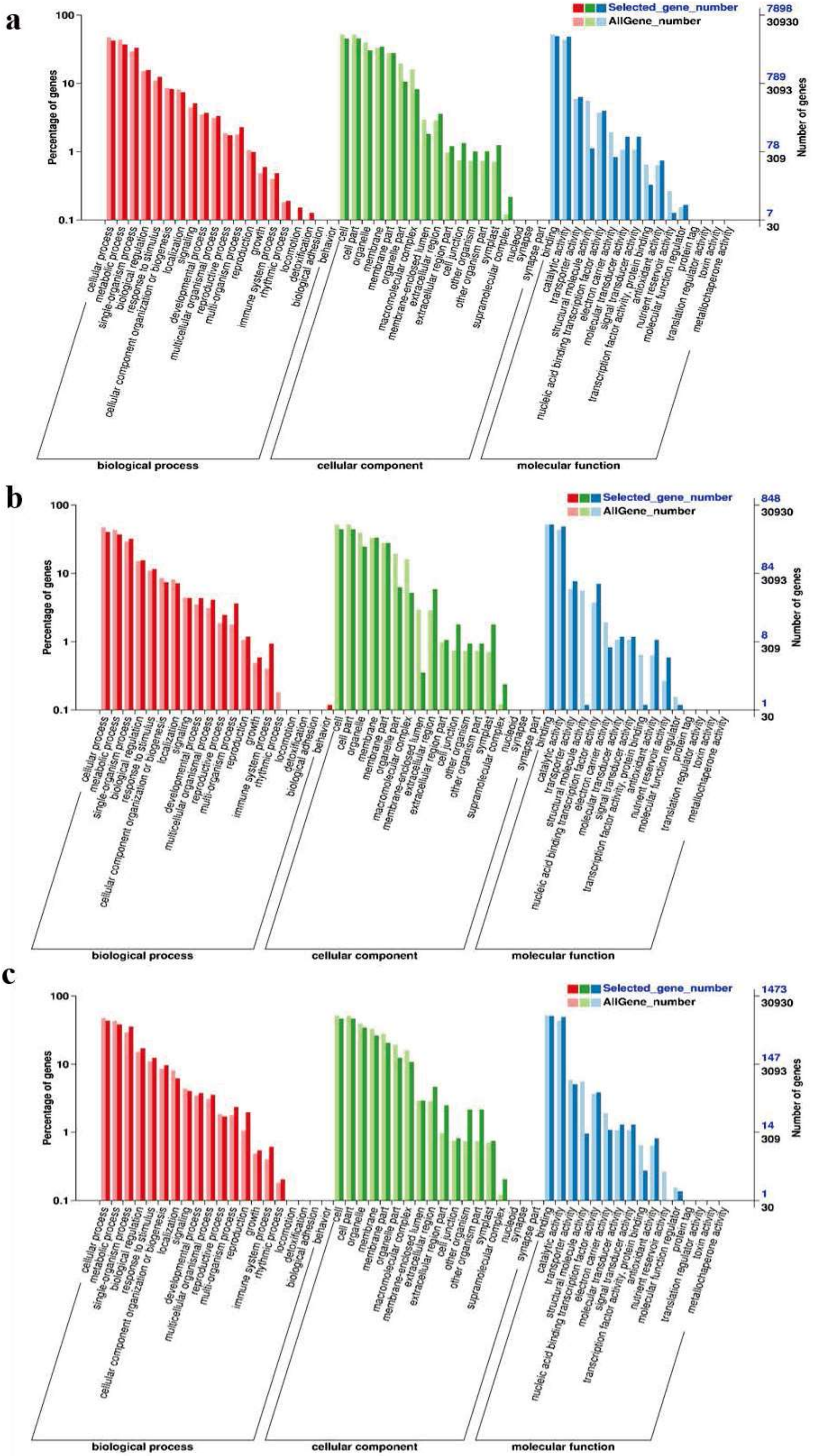
a
Selected_gene_number
AllGene_number
Percentage of genes
100
10
1
0.1
Number of genes
7898
30930
789
3093
78
309
7
30
cellular process
metabolic process
single-organism process
biological regulation
response to stimulus
cellular component organization or biogenesis
localization
signaling
developmental process
multicellular organismal process
reproductive process
multi-organism process
reproduction
growth
immune system process
rhythmic process
locomotion
detoxification
biological adhesion
behavior
cell
cell part
organelle
membrane
membrane part
organelle part
macromolecular complex
membrane-enclosed lumen
extracellular region
extracellular region part
cell junction
other organism
other organism part
symplast
supramolecular complex
nucleoid
synapse
synapse part
binding
catalytic activity
transporter activity
structural molecule activity
nucleic acid binding transcription factor activity
electron carrier activity
molecular transducer activity
signal transducer activity
transcription factor activity, protein binding
antioxidant activity
nutrient reservoir activity
molecular function regulator
protein tag
translation regulator activity
toxin activity
metallochaperone activity
biological process
cellular component
molecular function
b
Selected_gene_number
AllGene_number
Percentage of genes
100
10
1
0.1
Number of genes
848
30930
84
3093
8
309
1
30
cellular process
metabolic process
single-organism process
biological regulation
response to stimulus
cellular component organization or biogenesis
localization
signaling
developmental process
multicellular organismal process
reproductive process
multi-organism process
reproduction
growth
immune system process
rhythmic process
locomotion
detoxification
biological adhesion
behavior
cell
cell part
organelle
membrane
membrane part
organelle part
macromolecular complex
membrane-enclosed lumen
extracellular region
extracellular region part
cell junction
other organism
other organism part
symplast
supramolecular complex
nucleoid
synapse
synapse part
binding
catalytic activity
transporter activity
structural molecule activity
nucleic acid binding transcription factor activity
electron carrier activity
molecular transducer activity
signal transducer activity
transcription factor activity, protein binding
antioxidant activity
nutrient reservoir activity
molecular function regulator
protein tag
translation regulator activity
toxin activity
metallochaperone activity
biological process
cellular component
molecular function
c
Selected_gene_number
AllGene_number
Percentage of genes
100
10
1
0.1
Number of genes
1473
30930
147
3093
14
309
1
30
cellular process
metabolic process
single-organism process
biological regulation
response to stimulus
cellular component organization or biogenesis
localization
signaling
developmental process
multicellular organismal process
reproductive process
multi-organism process
reproduction
growth
immune system process
rhythmic process
locomotion
detoxification
biological adhesion
behavior
cell
cell part
organelle
membrane
membrane part
organelle part
macromolecular complex
membrane-enclosed lumen
extracellular region
extracellular region part
cell junction
other organism
other organism part
symplast
supramolecular complex
nucleoid
synapse
synapse part
binding
catalytic activity
transporter activity
structural molecule activity
nucleic acid binding transcription factor activity
electron carrier activity
molecular transducer activity
signal transducer activity
transcription factor activity, protein binding
antioxidant activity
nutrient reservoir activity
molecular function regulator
protein tag
translation regulator activity
toxin activity
metallochaperone activity
biological process
cellular component
molecular function

Supplementary Figure 13. GO enrichment analysis of all DEGs in leaf(a) and stem(b) development, and shared DEGs in leaf development(c).

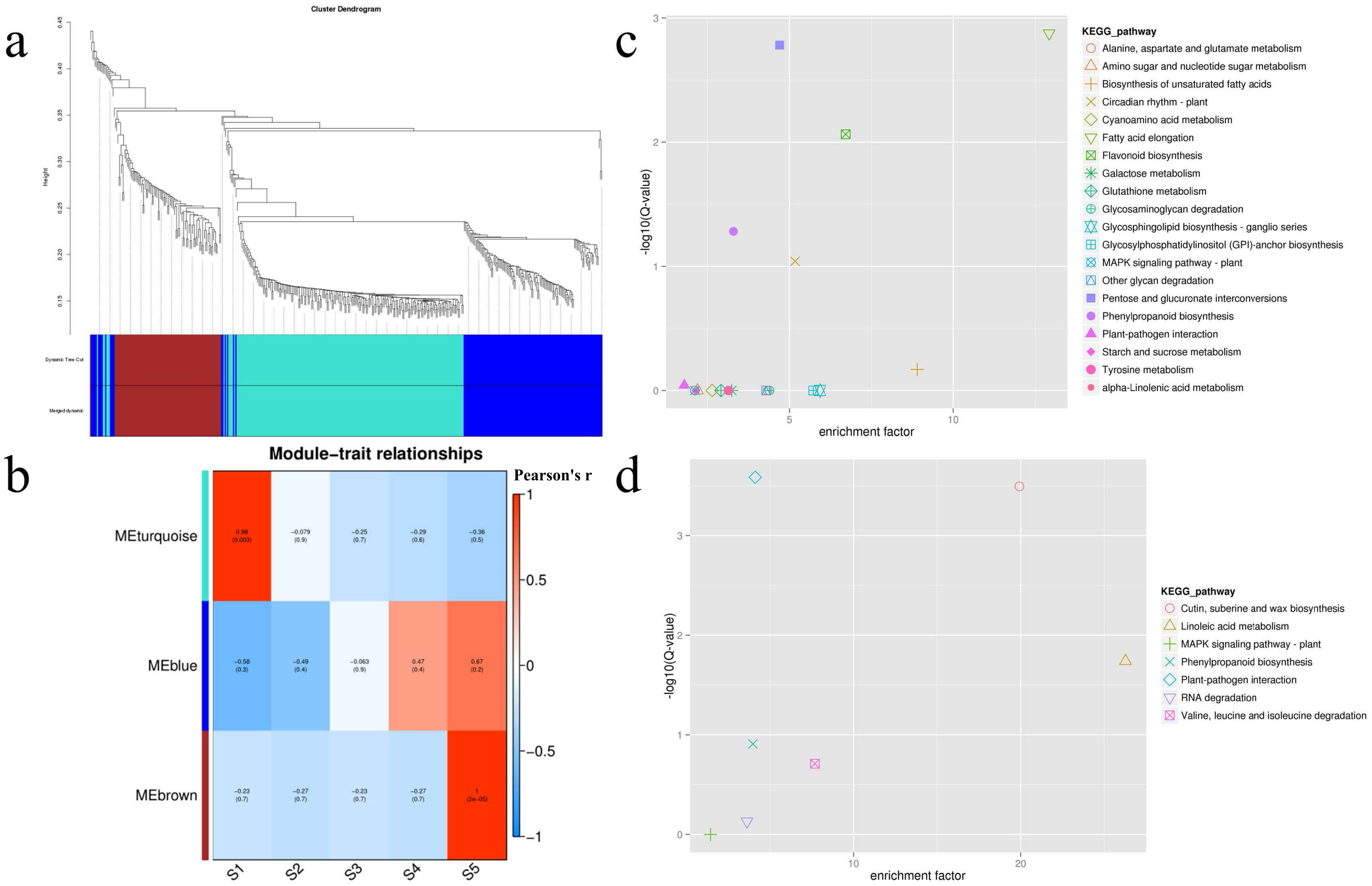
a
Cluster Dendrogram
Height
Dynamic Tree Cut
Merged dynamic
b
Module–trait relationships
Pearson's r
MEturquoise
MEblue
MEbrown
0.98 (0.003)
−0.079 (0.9)
−0.25 (0.7)
−0.29 (0.6)
−0.36 (0.5)
−0.58 (0.3)
−0.49 (0.4)
−0.063 (0.9)
0.47 (0.4)
0.67 (0.2)
−0.23 (0.7)
−0.27 (0.7)
−0.23 (0.7)
−0.27 (0.7)
1 (2e−05)
S1
S2
S3
S4
S5
c
-log10(Q-value)
enrichment factor
KEGG_pathway
Alanine, aspartate and glutamate metabolism
Amino sugar and nucleotide sugar metabolism
Biosynthesis of unsaturated fatty acids
Circadian rhythm - plant
Cyanoamino acid metabolism
Fatty acid elongation
Flavonoid biosynthesis
Galactose metabolism
Glutathione metabolism
Glycosaminoglycan degradation
Glycosphingolipid biosynthesis - ganglio series
Glycosylphosphatidylinositol (GPI)-anchor biosynthesis
MAPK signaling pathway - plant
Other glycan degradation
Pentose and glucuronate interconversions
Phenylpropanoid biosynthesis
Plant-pathogen interaction
Starch and sucrose metabolism
Tyrosine metabolism
alpha-Linolenic acid metabolism
d
-log10(Q-value)
enrichment factor
KEGG_pathway
Cutin, suberine and wax biosynthesis
Linoleic acid metabolism
MAPK signaling pathway - plant
Phenylpropanoid biosynthesis
Plant-pathogen interaction
RNA degradation
Valine, leucine and isoleucine degradation

Supplementary Figure 14. Weighted gene co-expression network analysis (WGCNA) of genes during the stem development. a, hierarchical cluster tree showing co-expression modules identified by WGCNA. b, module-stage association (each row corresponds to a module, and each column represents a specific stage. The color of each cell at the row column intersection indicates the Pearson correlation coefficient (Pearson's r) between a module and the stage), positive and negative correlations are shown in red and blue, respectively. c, KEGG enrichment analysis of genes in turquoise module. d, KEGG enrichment analysis of genes in brown module. Each sample for WGCNA had three biological replicates.

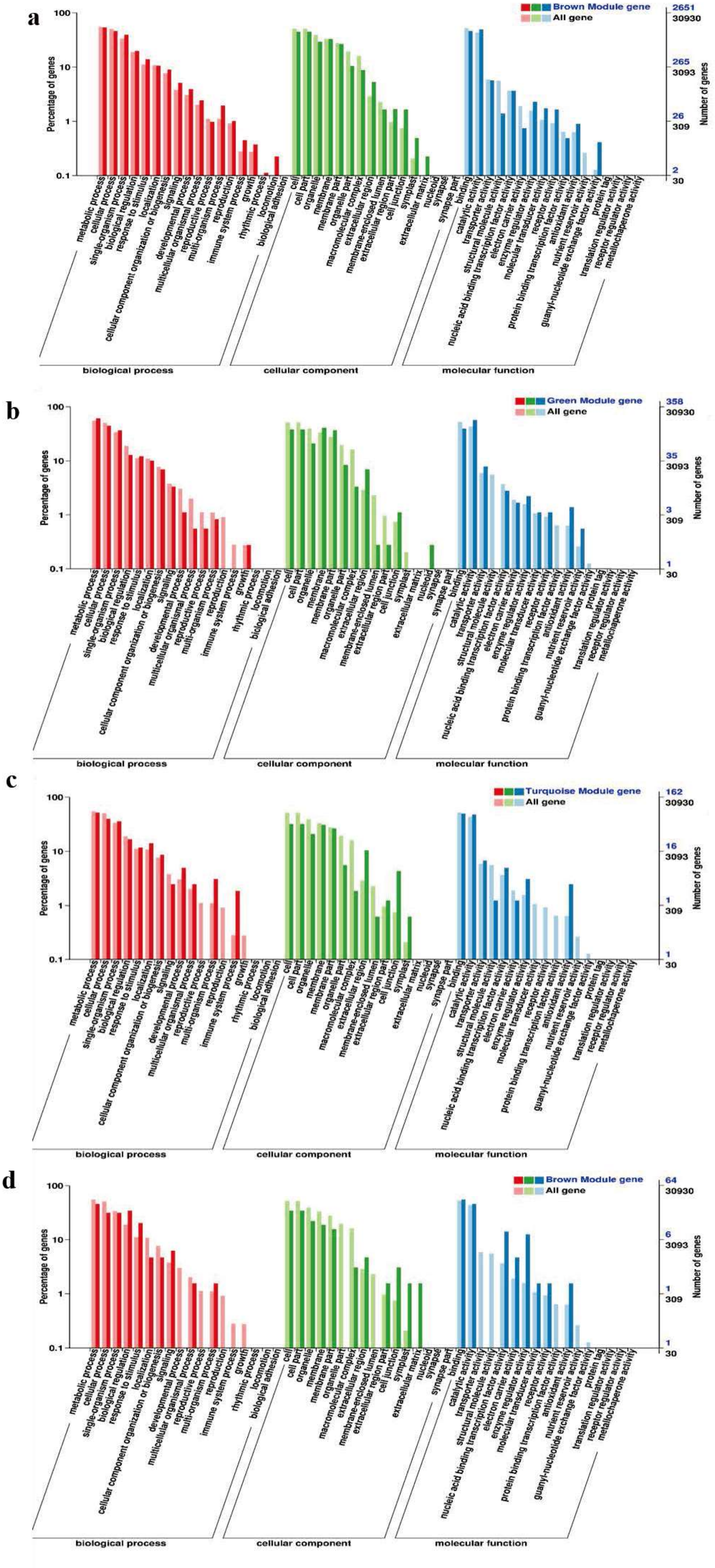
a
Brown Module gene
All gene
2651
30930
265
3093
26
309
2
30
Percentage of genes
Number of genes
100
10
1
0.1
metabolic process
cellular process
single-organism process
biological regulation
response to stimulus
localization
cellular component organization or biogenesis
signaling
developmental process
multicellular organismal process
reproductive process
multi-organism process
reproduction
immune system process
growth
rhythmic process
locomotion
biological adhesion
cell
cell part
organelle
membrane
membrane part
organelle part
macromolecular complex
extracellular region
membrane-enclosed lumen
extracellular region part
cell junction
symplast
extracellular matrix
nucleoid
synapse
synapse part
binding
catalytic activity
transporter activity
structural molecule activity
nucleic acid binding transcription factor activity
electron carrier activity
enzyme regulator activity
molecular transducer activity
receptor activity
protein binding transcription factor activity
antioxidant activity
nutrient reservoir activity
guanyl-nucleotide exchange factor activity
protein tag
translation regulator activity
receptor regulator activity
metallochaperone activity
biological process
cellular component
molecular function
b
Green Module gene
All gene
358
30930
35
3093
3
309
1
30
c
Turquoise Module gene
All gene
162
30930
16
3093
1
309
1
30
d
Brown Module gene
All gene
64
30930
6
3093
1
309
1
30

Supplementary Figure 15. GO enrichment analysis of the genes in brown(a) and green(b) module during the leaf development; and the genes in turquoise(c) and brown(d) module during the stem development.

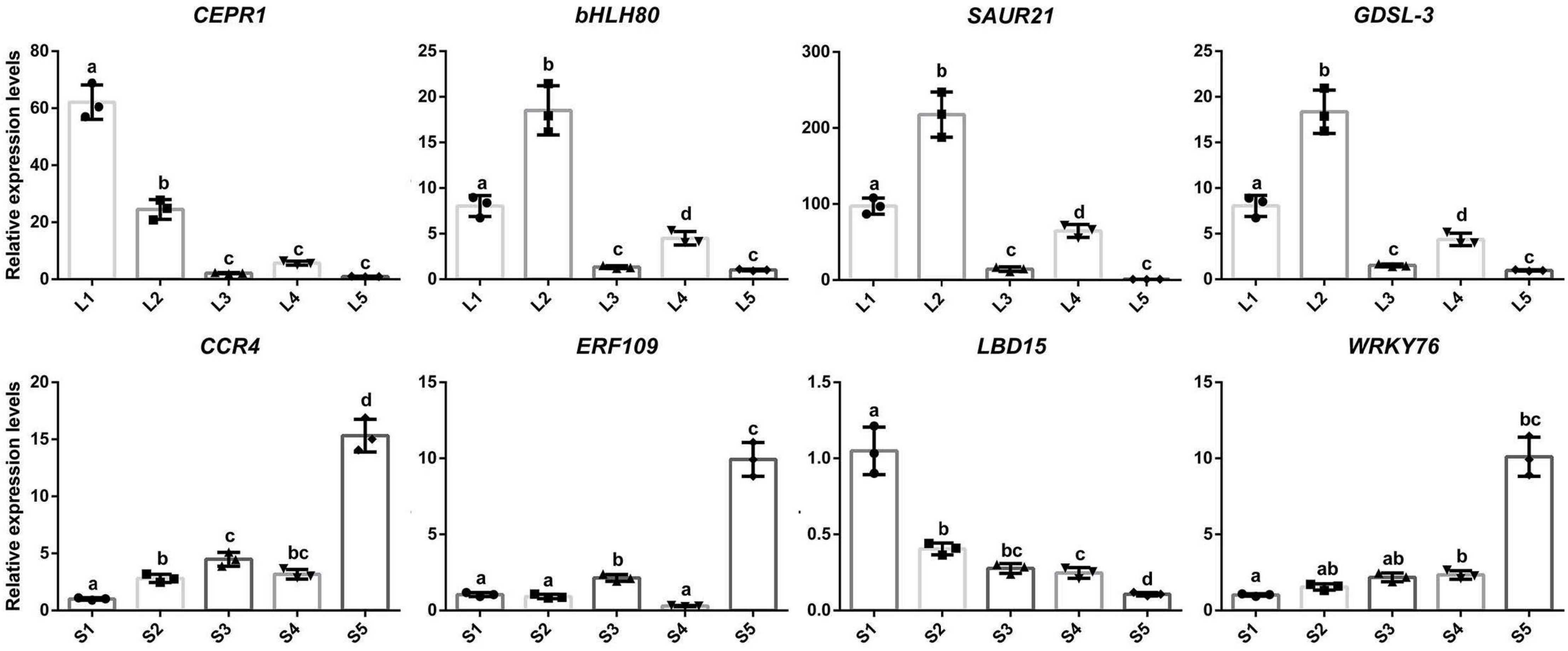

CEPR1
bHLH80
SAUR21
GDSL-3
CCR4
ERF109
LBD15
WRKY76
Relative expression levels
L1
L2
L3
L4
L5
S1
S2
S3
S4
S5

Supplementary Figure 16. Relative expression levels of some identified genes of WCGNA in different development stages of leaf (L1-L5) or stem (S1-S5). Expression profiles of these genes were analyzed using RT-qPCR analysis. The poplar *Ubiquitin* (*UBQ*) gene was used as an internal control and gene expression profiles were evaluated using the $2^{-\Delta\Delta Ct}$ method. Three biological replicates for each tissue were analyzed. Error bars represent the SD of the mean (n=3). Different letters above bars represent statistically significant differences between groups ($p < 0.05$) as determined by one-way ANOVA followed by Dunnett's test. In these groups, the same letter indicates that there is no significant difference between the two groups, different letters indicate that there is a significant difference between the two groups. For *CEPR1*, *bHLH80*, *SAUR1*, and *GDSL-3*, the relative expression level of L5 stage was set to be 1. For *CCR4*, *ERF109*, *LBD15*, and *WRKY76*, the relative expression level of S1 stage was set to be 1.

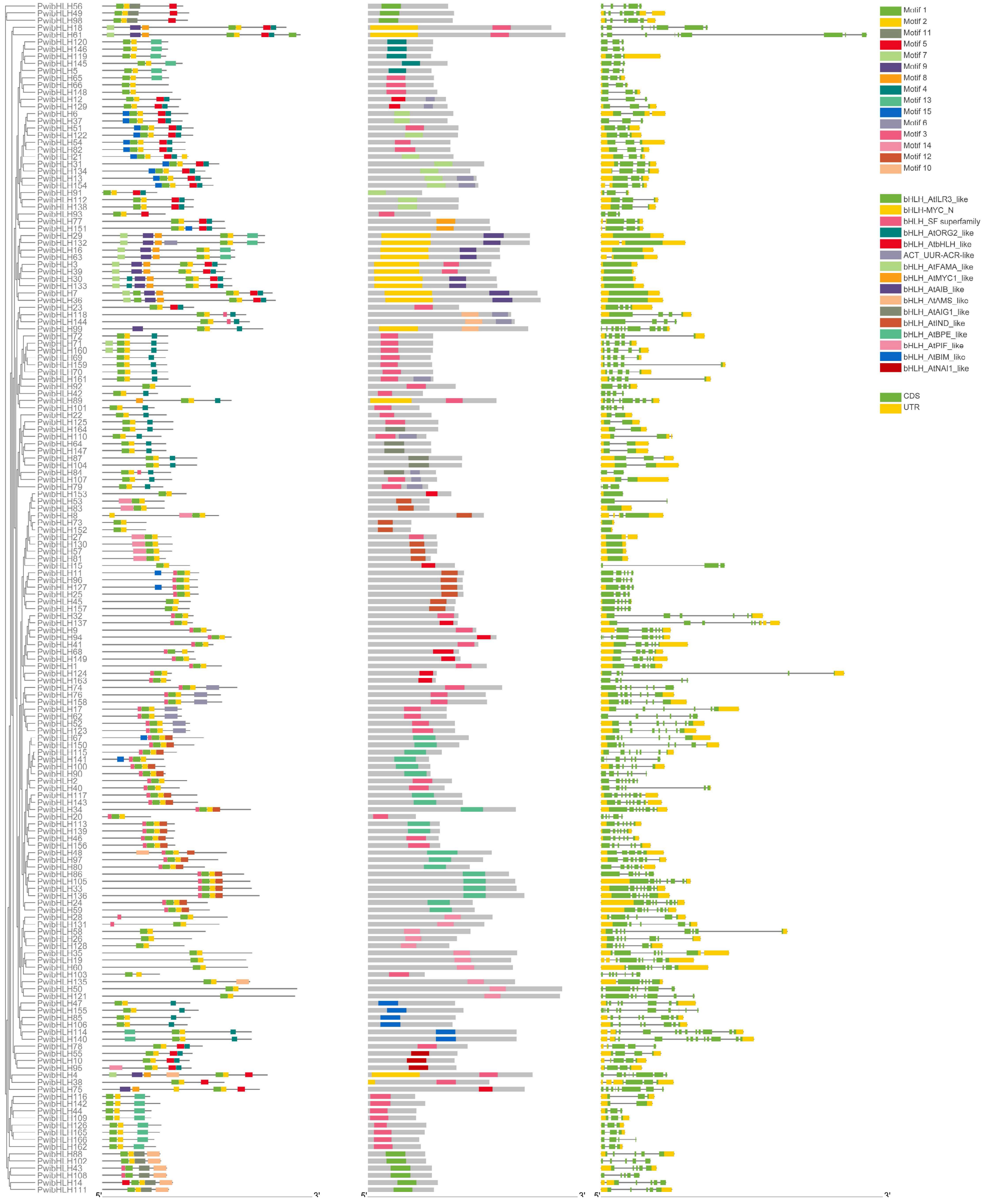

PwibHLH56
PwibHLH49
PwibHLH98
PwibHLH18
PwibHLH61
PwibHLH120
PwibHLH146
PwibHLH119
PwibHLH145
PwibHLH5
PwibHLH65
PwibHLH66
PwibHLH148
PwibHLH12
PwibHLH129
PwibHLH6
PwibHLH37
PwibHLH51
PwibHLH122
PwibHLH54
PwibHLH82
PwibHLH21
PwibHLH31
PwibHLH134
PwibHLH13
PwibHLH154
PwibHLH91
PwibHLH112
PwibHLH138
PwibHLH93
PwibHLH77
PwibHLH151
PwibHLH29
PwibHLH132
PwibHLH16
PwibHLH63
PwibHLH3
PwibHLH39
PwibHLH30
PwibHLH133
PwibHLH7
PwibHLH36
PwibHLH23
PwibHLH118
PwibHLH144
PwibHLH99
PwibHLH72
PwibHLH71
PwibHLH160
PwibHLH69
PwibHLH159
PwibHLH70
PwibHLH161
PwibHLH92
PwibHLH42
PwibHLH89
PwibHLH101
PwibHLH22
PwibHLH125
PwibHLH164
PwibHLH110
PwibHLH64
PwibHLH147
PwibHLH87
PwibHLH104
PwibHLH84
PwibHLH107
PwibHLH79
PwibHLH153
PwibHLH53
PwibHLH83
PwibHLH8
PwibHLH73
PwibHLH152
PwibHLH27
PwibHLH130
PwibHLH57
PwibHLH81
PwibHLH15
PwibHLH11
PwibHLH96
PwibHLH127
PwibHLH25
PwibHLH45
PwibHLH157
PwibHLH32
PwibHLH137
PwibHLH9
PwibHLH94
PwibHLH41
PwibHLH68
PwibHLH149
PwibHLH1
PwibHLH124
PwibHLH163
PwibHLH74
PwibHLH76
PwibHLH158
PwibHLH17
PwibHLH62
PwibHLH52
PwibHLH123
PwibHLH67
PwibHLH150
PwibHLH115
PwibHLH141
PwibHLH100
PwibHLH90
PwibHLH2
PwibHLH40
PwibHLH117
PwibHLH143
PwibHLH34
PwibHLH20
PwibHLH113
PwibHLH139
PwibHLH46
PwibHLH156
PwibHLH48
PwibHLH97
PwibHLH80
PwibHLH86
PwibHLH105
PwibHLH33
PwibHLH136
PwibHLH24
PwibHLH59
PwibHLH28
PwibHLH131
PwibHLH58
PwibHLH26
PwibHLH128
PwibHLH35
PwibHLH19
PwibHLH60
PwibHLH103
PwibHLH135
PwibHLH50
PwibHLH121
PwibHLH47
PwibHLH155
PwibHLH85
PwibHLH106
PwibHLH114
PwibHLH140
PwibHLH78
PwibHLH55
PwibHLH10
PwibHLH95
PwibHLH4
PwibHLH38
PwibHLH75
PwibHLH116
PwibHLH142
PwibHLH44
PwibHLH109
PwibHLH126
PwibHLH165
PwibHLH166
PwibHLH162
PwibHLH88
PwibHLH102
PwibHLH43
PwibHLH108
PwibHLH14
PwibHLH111
5'
3'
5'
3'
5'
3'
Motif 1
Motif 2
Motif 11
Motif 5
Motif 7
Motif 9
Motif 8
Motif 4
Motif 13
Motif 15
Motif 6
Motif 3
Motif 14
Motif 12
Motif 10
bHLH_AtILR3_like
bHLH-MYC_N
bHLH_SF superfamily
bHLH_AtORG2_like
bHLH_AtbHLH_like
ACT_UUR-ACR-like
bHLH_AtFAMA_like
bHLH_AtMYC1_like
bHLH_AtAIB_like
bHLH_AtAMS_like
bHLH_AtAIG1_like
bHLH_AtIND_like
bHLH_AtBPE_like
bHLH_AtPIF_like
bHLH_AtBIM_like
bHLH_AtNAI1_like
CDS
UTR

Supplementary Figure 17. The gene structures and conserved motifs of *Populus wilsonii* bHLH family members. From left to right of this panel are phylogenetic trees, conserved motifs, conserved domains, and gene structures of the PwibHLH members.

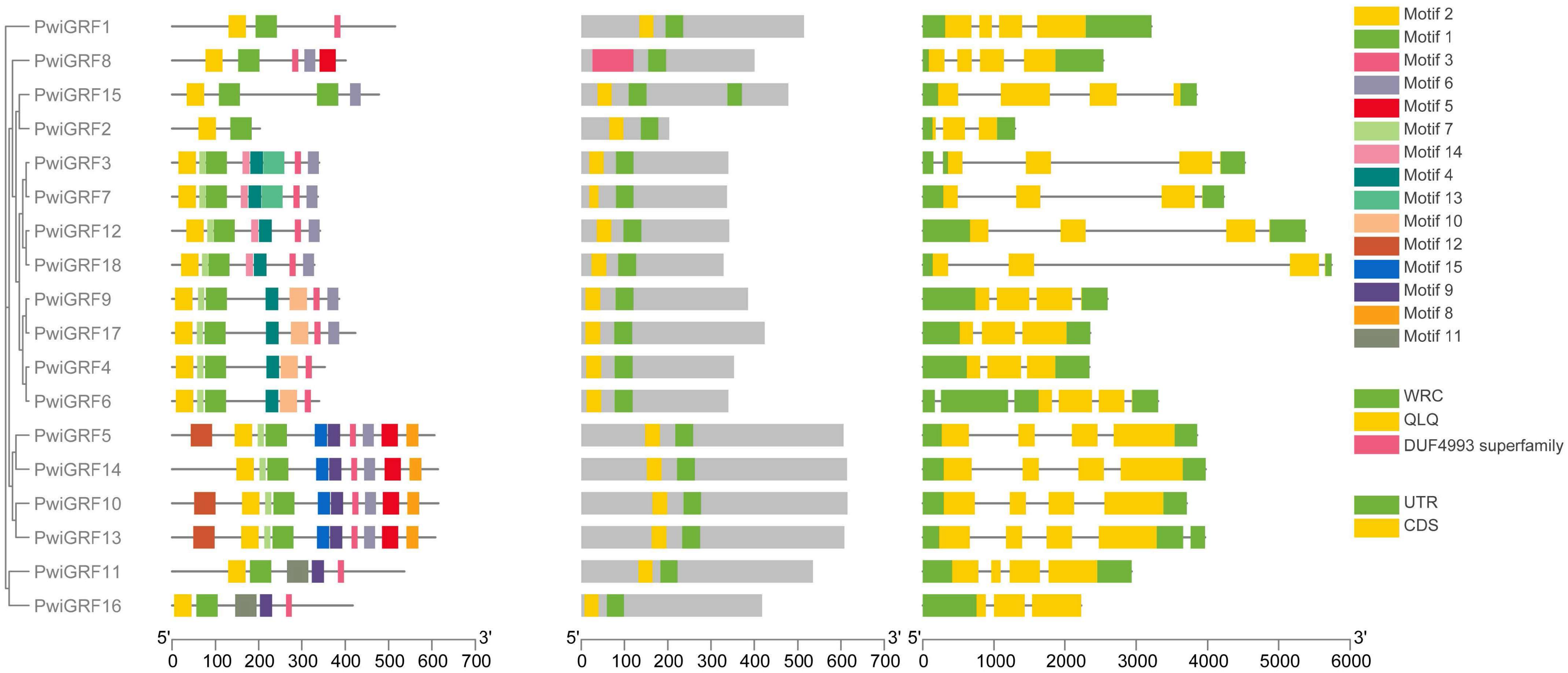

PwiGRF1
PwiGRF8
PwiGRF15
PwiGRF2
PwiGRF3
PwiGRF7
PwiGRF12
PwiGRF18
PwiGRF9
PwiGRF17
PwiGRF4
PwiGRF6
PwiGRF5
PwiGRF14
PwiGRF10
PwiGRF13
PwiGRF11
PwiGRF16
5' 3'
0 100 200 300 400 500 600 700
5' 3'
0 100 200 300 400 500 600 700
5' 3'
0 1000 2000 3000 4000 5000 6000
Motif 2
Motif 1
Motif 3
Motif 6
Motif 5
Motif 7
Motif 14
Motif 4
Motif 13
Motif 10
Motif 12
Motif 15
Motif 9
Motif 8
Motif 11
WRC
QLQ
DUF4993 superfamily
UTR
CDS

Supplementary Figure 18. The gene structures and conserved motifs of *Populus wilsonii* GRF family members. From left to right of this panel are phylogenetic trees, conserved motifs, conserved domains, and gene structures of the PwiGRF members.

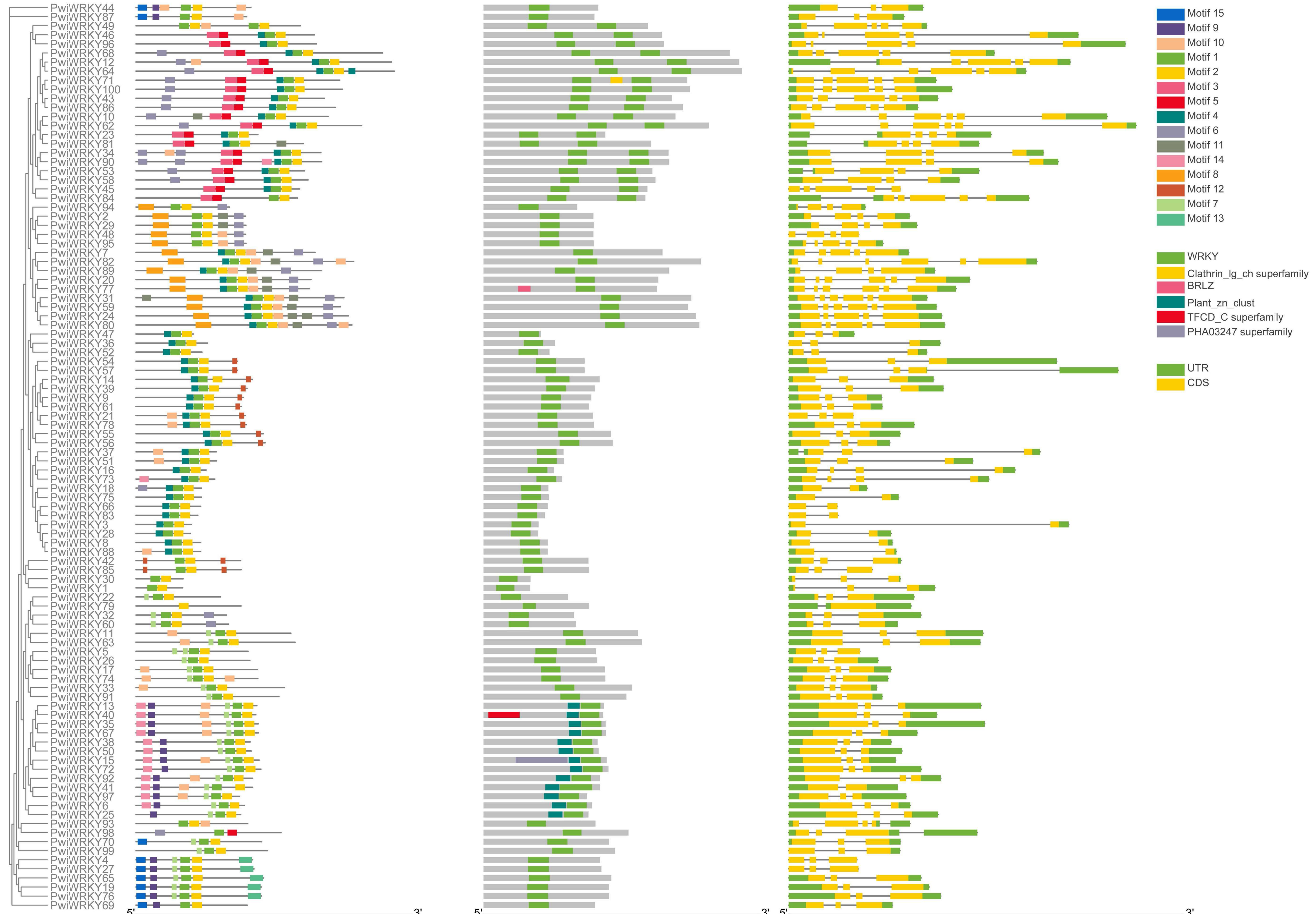

PwiWRKY44
PwiWRKY87
PwiWRKY49
PwiWRKY46
PwiWRKY96
PwiWRKY68
PwiWRKY12
PwiWRKY64
PwiWRKY71
PwiWRKY100
PwiWRKY43
PwiWRKY86
PwiWRKY10
PwiWRKY62
PwiWRKY23
PwiWRKY81
PwiWRKY34
PwiWRKY90
PwiWRKY53
PwiWRKY58
PwiWRKY45
PwiWRKY84
PwiWRKY94
PwiWRKY2
PwiWRKY29
PwiWRKY48
PwiWRKY95
PwiWRKY7
PwiWRKY82
PwiWRKY89
PwiWRKY20
PwiWRKY77
PwiWRKY31
PwiWRKY59
PwiWRKY24
PwiWRKY80
PwiWRKY47
PwiWRKY36
PwiWRKY52
PwiWRKY54
PwiWRKY57
PwiWRKY14
PwiWRKY39
PwiWRKY9
PwiWRKY61
PwiWRKY21
PwiWRKY78
PwiWRKY55
PwiWRKY56
PwiWRKY37
PwiWRKY51
PwiWRKY16
PwiWRKY73
PwiWRKY18
PwiWRKY75
PwiWRKY66
PwiWRKY83
PwiWRKY3
PwiWRKY28
PwiWRKY8
PwiWRKY88
PwiWRKY42
PwiWRKY85
PwiWRKY30
PwiWRKY1
PwiWRKY22
PwiWRKY79
PwiWRKY32
PwiWRKY60
PwiWRKY11
PwiWRKY63
PwiWRKY5
PwiWRKY26
PwiWRKY17
PwiWRKY74
PwiWRKY33
PwiWRKY91
PwiWRKY13
PwiWRKY40
PwiWRKY35
PwiWRKY67
PwiWRKY38
PwiWRKY50
PwiWRKY15
PwiWRKY72
PwiWRKY92
PwiWRKY41
PwiWRKY97
PwiWRKY6
PwiWRKY25
PwiWRKY93
PwiWRKY98
PwiWRKY70
PwiWRKY99
PwiWRKY4
PwiWRKY27
PwiWRKY65
PwiWRKY19
PwiWRKY76
PwiWRKY69
5'
3'
5'
3'
5'
3'
Motif 15
Motif 9
Motif 10
Motif 1
Motif 2
Motif 3
Motif 5
Motif 4
Motif 6
Motif 11
Motif 14
Motif 8
Motif 12
Motif 7
Motif 13
WRKY
Clathrin_lg_ch superfamily
BRLZ
Plant_zn_clust
TFCD_C superfamily
PHA03247 superfamily
UTR
CDS

Supplementary Figure 19. The gene structures and conserved motifs of *Populus wilsonii* WRKY family members. From left to right of this panel are phylogenetic trees, conserved motifs, conserved domains, and gene structures of the PwiGRF members.

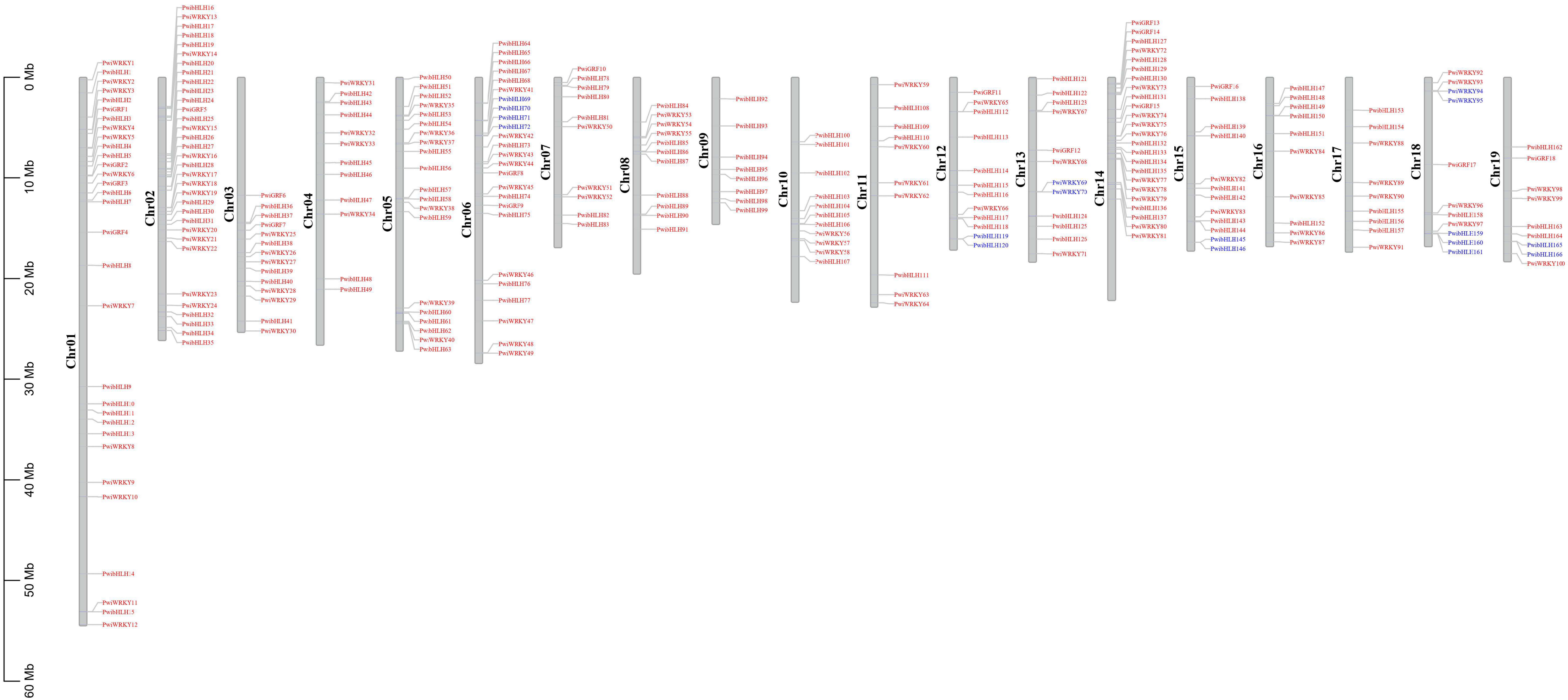

0 Mb
10 Mb
20 Mb
30 Mb
40 Mb
50 Mb
60 Mb
Chr01
PwiWRKY1
PwibHLH1
PwiWRKY2
PwiWRKY3
PwibHLH2
PwiGRF1
PwibHLH3
PwiWRKY4
PwiWRKY5
PwibHLH4
PwibHLH5
PwiGRF2
PwiWRKY6
PwiGRF3
PwibHLH6
PwibHLH7
PwiGRF4
PwibHLH8
PwiWRKY7
PwibHLH9
PwibHLH10
PwibHLH11
PwibHLH12
PwibHLH13
PwiWRKY8
PwiWRKY9
PwiWRKY10
PwibHLH14
PwiWRKY11
PwibHLH15
PwiWRKY12
Chr02
PwibHLH16
PwiWRKY13
PwibHLH17
PwibHLH18
PwibHLH19
PwiWRKY14
PwibHLH20
PwibHLH21
PwibHLH22
PwibHLH23
PwibHLH24
PwiGRF5
PwibHLH25
PwiWRKY15
PwibHLH26
PwibHLH27
PwiWRKY16
PwibHLH28
PwiWRKY17
PwiWRKY18
PwiWRKY19
PwibHLH29
PwibHLH30
PwibHLH31
PwiWRKY20
PwiWRKY21
PwiWRKY22
PwiWRKY23
PwiWRKY24
PwibHLH32
PwibHLH33
PwibHLH34
PwibHLH35
Chr03
PwiGRF6
PwibHLH36
PwibHLH37
PwiGRF7
PwiWRKY25
PwibHLH38
PwiWRKY26
PwiWRKY27
PwibHLH39
PwibHLH40
PwiWRKY28
PwiWRKY29
PwibHLH41
PwiWRKY30
Chr04
PwiWRKY31
PwibHLH42
PwibHLH43
PwibHLH44
PwiWRKY32
PwiWRKY33
PwibHLH45
PwibHLH46
PwibHLH47
PwiWRKY34
PwibHLH48
PwibHLH49
Chr05
PwibHLH50
PwibHLH51
PwibHLH52
PwiWRKY35
PwibHLH53
PwibHLH54
PwiWRKY36
PwiWRKY37
PwibHLH55
PwibHLH56
PwibHLH57
PwibHLH58
PwiWRKY38
PwibHLH59
PwiWRKY39
PwibHLH60
PwibHLH61
PwibHLH62
PwiWRKY40
PwibHLH63
Chr06
PwibHLH64
PwibHLH65
PwibHLH66
PwibHLH67
PwibHLH68
PwiWRKY41
PwibHLH69
PwibHLH70
PwibHLH71
PwibHLH72
PwiWRKY42
PwibHLH73
PwiWRKY43
PwiWRKY44
PwiGRF8
PwiWRKY45
PwibHLH74
PwiGRF9
PwibHLH75
PwiWRKY46
PwibHLH76
PwibHLH77
PwiWRKY47
PwiWRKY48
PwiWRKY49
Chr07
PwiGRF10
PwibHLH78
PwibHLH79
PwibHLH80
PwibHLH81
PwiWRKY50
PwiWRKY51
PwiWRKY52
PwibHLH82
PwibHLH83
Chr08
PwibHLH84
PwiWRKY53
PwiWRKY54
PwiWRKY55
PwibHLH85
PwibHLH86
PwibHLH87
PwibHLH88
PwibHLH89
PwibHLH90
PwibHLH91
Chr09
PwibHLH92
PwibHLH93
PwibHLH94
PwibHLH95
PwibHLH96
PwibHLH97
PwibHLH98
PwibHLH99
Chr10
PwibHLH100
PwibHLH101
PwibHLH102
PwibHLH103
PwibHLH104
PwibHLH105
PwibHLH106
PwiWRKY56
PwiWRKY57
PwiWRKY58
PwibHLH107
Chr11
PwiWRKY59
PwibHLH108
PwibHLH109
PwibHLH110
PwiWRKY60
PwiWRKY61
PwiWRKY62
PwibHLH111
PwiWRKY63
PwiWRKY64
Chr12
PwiGRF11
PwiWRKY65
PwibHLH112
PwibHLH113
PwibHLH114
PwibHLH115
PwibHLH116
PwiWRKY66
PwibHLH117
PwibHLH118
PwibHLH119
PwibHLH120
Chr13
PwibHLH121
PwibHLH122
PwibHLH123
PwiWRKY67
PwiGRF12
PwiWRKY68
PwiWRKY69
PwiWRKY70
PwibHLH124
PwibHLH125
PwibHLH126
PwiWRKY71
Chr14
PwiGRF13
PwiGRF14
PwibHLH127
PwiWRKY72
PwibHLH128
PwibHLH129
PwibHLH130
PwiWRKY73
PwibHLH131
PwiGRF15
PwiWRKY74
PwiWRKY75
PwiWRKY76
PwibHLH132
PwibHLH133
PwibHLH134
PwibHLH135
PwiWRKY77
PwiWRKY78
PwiWRKY79
PwibHLH136
PwibHLH137
PwiWRKY80
PwiWRKY81
Chr15
PwiGRF16
PwibHLH138
PwibHLH139
PwibHLH140
PwiWRKY82
PwibHLH141
PwibHLH142
PwiWRKY83
PwibHLH143
PwibHLH144
PwibHLH145
PwibHLH146
Chr16
PwibHLH147
PwibHLH148
PwibHLH149
PwibHLH150
PwibHLH151
PwiWRKY84
PwiWRKY85
PwibHLH152
PwiWRKY86
PwiWRKY87
Chr17
PwibHLH153
PwibHLH154
PwiWRKY88
PwiWRKY89
PwiWRKY90
PwibHLH155
PwibHLH156
PwibHLH157
PwiWRKY91
Chr18
PwiWRKY92
PwiWRKY93
PwiWRKY94
PwiWRKY95
PwiGRF17
PwiWRKY96
PwibHLH158
PwiWRKY97
PwibHLH159
PwibHLH160
PwibHLH161
Chr19
PwibHLH162
PwiGRF18
PwiWRKY98
PwiWRKY99
PwibHLH163
PwibHLH164
PwibHLH165
PwibHLH166
PwiWRKY100

Supplementary Figure 20. Chromosomal locations of *Populus wilsonii* bHLH (PwibHLH), GRF (PwiGRF) and WRKY (PwiWRKY) gene families. Tandem duplicates are represented by blue font.

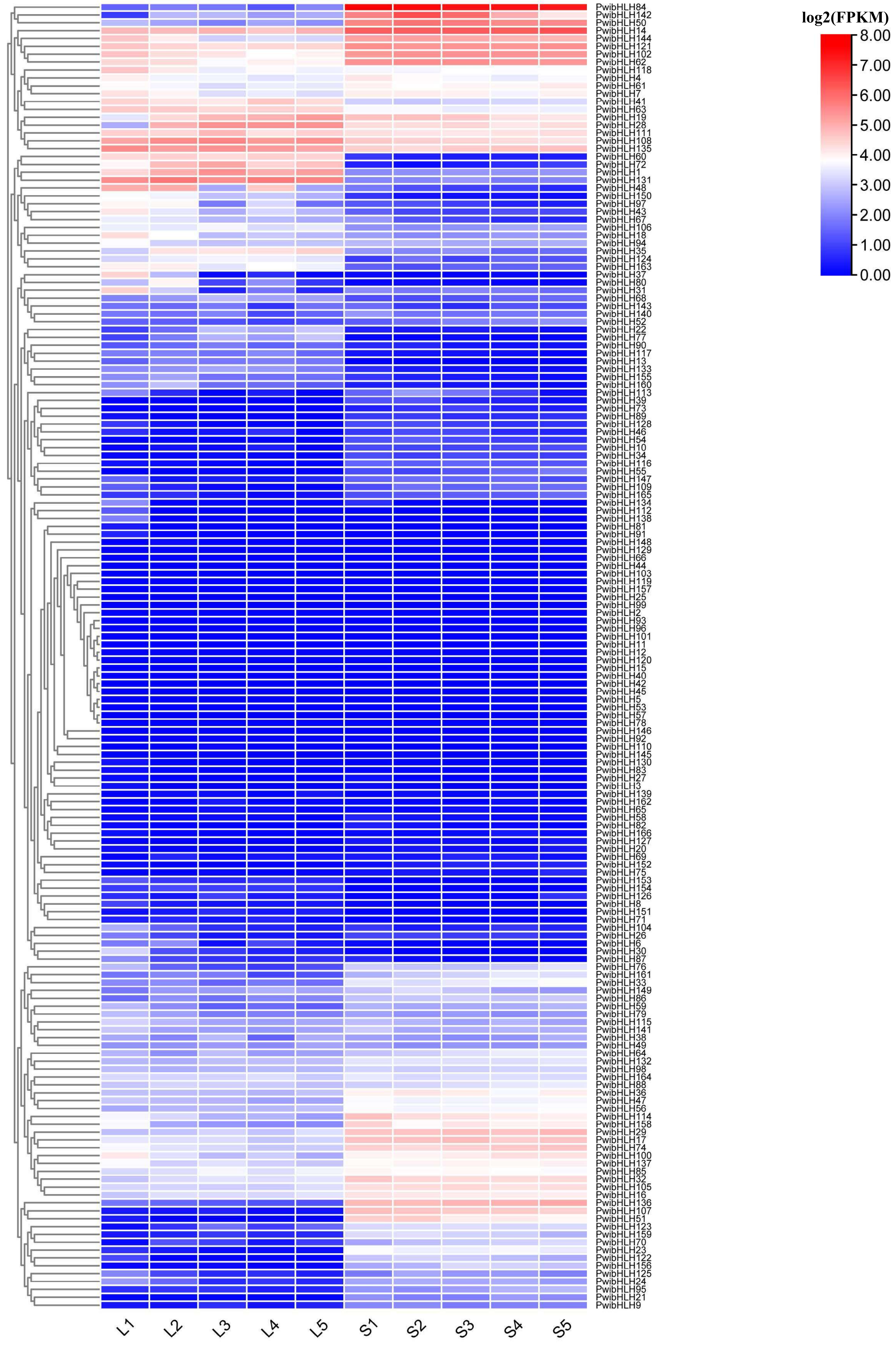
log2(FPKM)
8.00
7.00
6.00
5.00
4.00
3.00
2.00
1.00
0.00
PwibHLH84
PwibHLH142
PwibHLH50
PwibHLH14
PwibHLH144
PwibHLH121
PwibHLH102
PwibHLH62
PwibHLH118
PwibHLH4
PwibHLH61
PwibHLH7
PwibHLH41
PwibHLH63
PwibHLH19
PwibHLH28
PwibHLH111
PwibHLH108
PwibHLH135
PwibHLH60
PwibHLH72
PwibHLH1
PwibHLH131
PwibHLH48
PwibHLH150
PwibHLH97
PwibHLH43
PwibHLH67
PwibHLH106
PwibHLH18
PwibHLH94
PwibHLH35
PwibHLH124
PwibHLH163
PwibHLH37
PwibHLH80
PwibHLH31
PwibHLH68
PwibHLH143
PwibHLH140
PwibHLH52
PwibHLH22
PwibHLH77
PwibHLH90
PwibHLH117
PwibHLH13
PwibHLH133
PwibHLH155
PwibHLH160
PwibHLH113
PwibHLH39
PwibHLH73
PwibHLH89
PwibHLH128
PwibHLH46
PwibHLH54
PwibHLH10
PwibHLH34
PwibHLH116
PwibHLH55
PwibHLH147
PwibHLH109
PwibHLH165
PwibHLH134
PwibHLH112
PwibHLH138
PwibHLH81
PwibHLH91
PwibHLH148
PwibHLH129
PwibHLH66
PwibHLH44
PwibHLH103
PwibHLH119
PwibHLH157
PwibHLH25
PwibHLH99
PwibHLH2
PwibHLH93
PwibHLH96
PwibHLH101
PwibHLH11
PwibHLH12
PwibHLH120
PwibHLH15
PwibHLH40
PwibHLH42
PwibHLH45
PwibHLH5
PwibHLH53
PwibHLH57
PwibHLH78
PwibHLH146
PwibHLH92
PwibHLH110
PwibHLH145
PwibHLH130
PwibHLH83
PwibHLH27
PwibHLH3
PwibHLH139
PwibHLH162
PwibHLH65
PwibHLH58
PwibHLH82
PwibHLH166
PwibHLH127
PwibHLH20
PwibHLH69
PwibHLH152
PwibHLH75
PwibHLH153
PwibHLH154
PwibHLH126
PwibHLH8
PwibHLH151
PwibHLH71
PwibHLH104
PwibHLH26
PwibHLH6
PwibHLH30
PwibHLH87
PwibHLH76
PwibHLH161
PwibHLH33
PwibHLH149
PwibHLH86
PwibHLH59
PwibHLH79
PwibHLH115
PwibHLH141
PwibHLH38
PwibHLH49
PwibHLH64
PwibHLH132
PwibHLH98
PwibHLH164
PwibHLH88
PwibHLH36
PwibHLH47
PwibHLH56
PwibHLH114
PwibHLH158
PwibHLH29
PwibHLH17
PwibHLH74
PwibHLH100
PwibHLH137
PwibHLH85
PwibHLH32
PwibHLH105
PwibHLH16
PwibHLH136
PwibHLH107
PwibHLH51
PwibHLH123
PwibHLH159
PwibHLH70
PwibHLH23
PwibHLH122
PwibHLH156
PwibHLH125
PwibHLH24
PwibHLH95
PwibHLH21
PwibHLH9
L1
L2
L3
L4
L5
S1
S2
S3
S4
S5

Supplementary Figure 21. Heatmap showing the expression level of bHLH gene members in different development stages of leaf and stem in *Populus wilsonii*. The FPKM values are log2-based. Red and blue indicate high and low expression levels, respectively. Each sample for every development stage had three biological replicates.

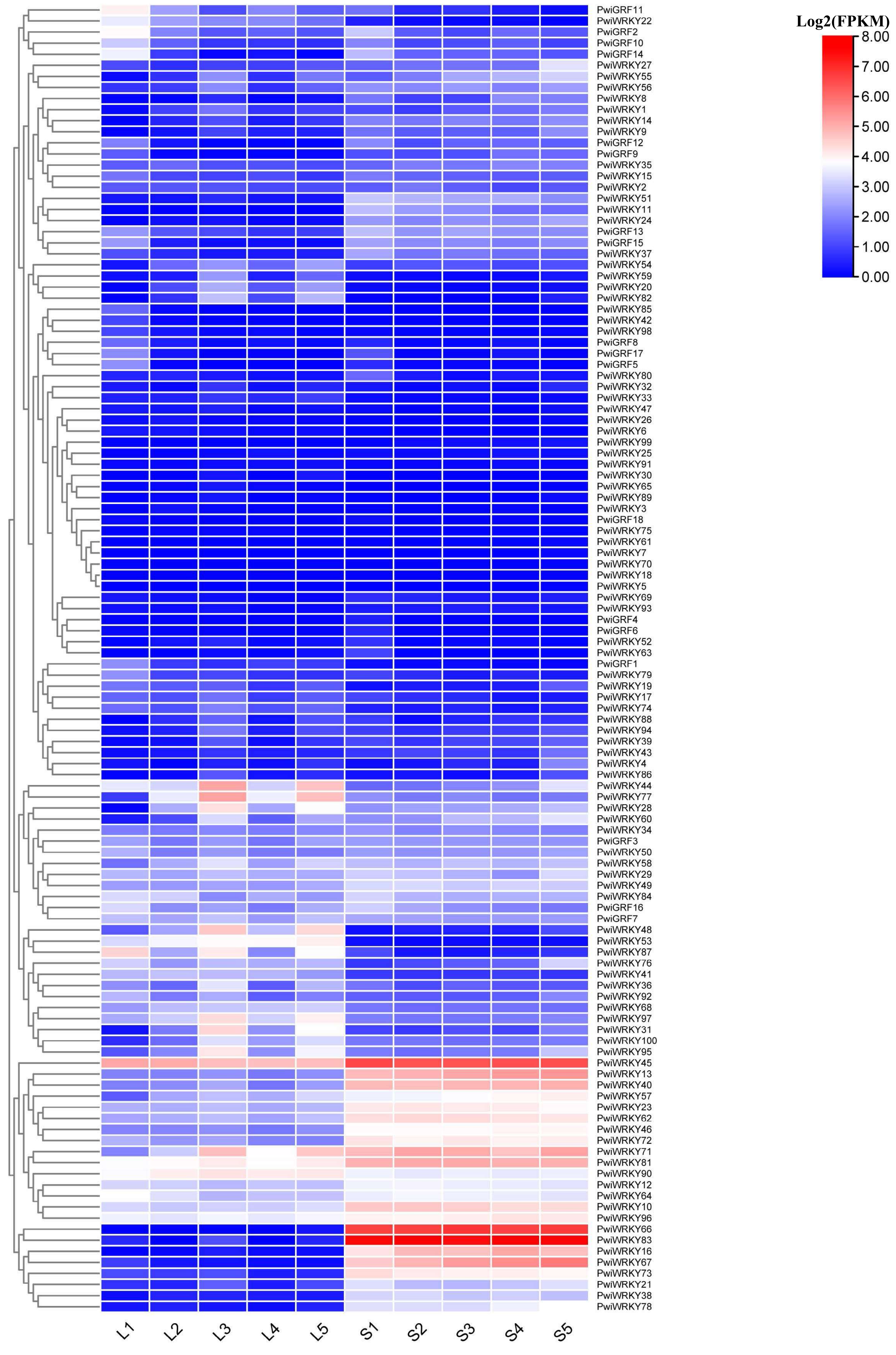
Log2(FPKM)
8.00
7.00
6.00
5.00
4.00
3.00
2.00
1.00
0.00
PwiGRF11
PwiWRKY22
PwiGRF2
PwiGRF10
PwiGRF14
PwiWRKY27
PwiWRKY55
PwiWRKY56
PwiWRKY8
PwiWRKY1
PwiWRKY14
PwiWRKY9
PwiGRF12
PwiGRF9
PwiWRKY35
PwiWRKY15
PwiWRKY2
PwiWRKY51
PwiWRKY11
PwiWRKY24
PwiGRF13
PwiGRF15
PwiWRKY37
PwiWRKY54
PwiWRKY59
PwiWRKY20
PwiWRKY82
PwiWRKY85
PwiWRKY42
PwiWRKY98
PwiGRF8
PwiGRF17
PwiGRF5
PwiWRKY80
PwiWRKY32
PwiWRKY33
PwiWRKY47
PwiWRKY26
PwiWRKY6
PwiWRKY99
PwiWRKY25
PwiWRKY91
PwiWRKY30
PwiWRKY65
PwiWRKY89
PwiWRKY3
PwiGRF18
PwiWRKY75
PwiWRKY61
PwiWRKY7
PwiWRKY70
PwiWRKY18
PwiWRKY5
PwiWRKY69
PwiWRKY93
PwiGRF4
PwiGRF6
PwiWRKY52
PwiWRKY63
PwiGRF1
PwiWRKY79
PwiWRKY19
PwiWRKY17
PwiWRKY74
PwiWRKY88
PwiWRKY94
PwiWRKY39
PwiWRKY43
PwiWRKY4
PwiWRKY86
PwiWRKY44
PwiWRKY77
PwiWRKY28
PwiWRKY60
PwiWRKY34
PwiGRF3
PwiWRKY50
PwiWRKY58
PwiWRKY29
PwiWRKY49
PwiWRKY84
PwiGRF16
PwiGRF7
PwiWRKY48
PwiWRKY53
PwiWRKY87
PwiWRKY76
PwiWRKY41
PwiWRKY36
PwiWRKY92
PwiWRKY68
PwiWRKY97
PwiWRKY31
PwiWRKY100
PwiWRKY95
PwiWRKY45
PwiWRKY13
PwiWRKY40
PwiWRKY57
PwiWRKY23
PwiWRKY62
PwiWRKY46
PwiWRKY72
PwiWRKY71
PwiWRKY81
PwiWRKY90
PwiWRKY12
PwiWRKY64
PwiWRKY10
PwiWRKY96
PwiWRKY66
PwiWRKY83
PwiWRKY16
PwiWRKY67
PwiWRKY73
PwiWRKY21
PwiWRKY38
PwiWRKY78
L1
L2
L3
L4
L5
S1
S2
S3
S4
S5

Supplementary Figure 22. Heatmap showing the expression level of GRF and WRKY gene members in different development stages of leaf and stem in *Populus wilsonii*. The FPKM values are log2-based. Red and blue indicate high and low expression levels, respectively. Each sample for every development stage had three biological replicates.

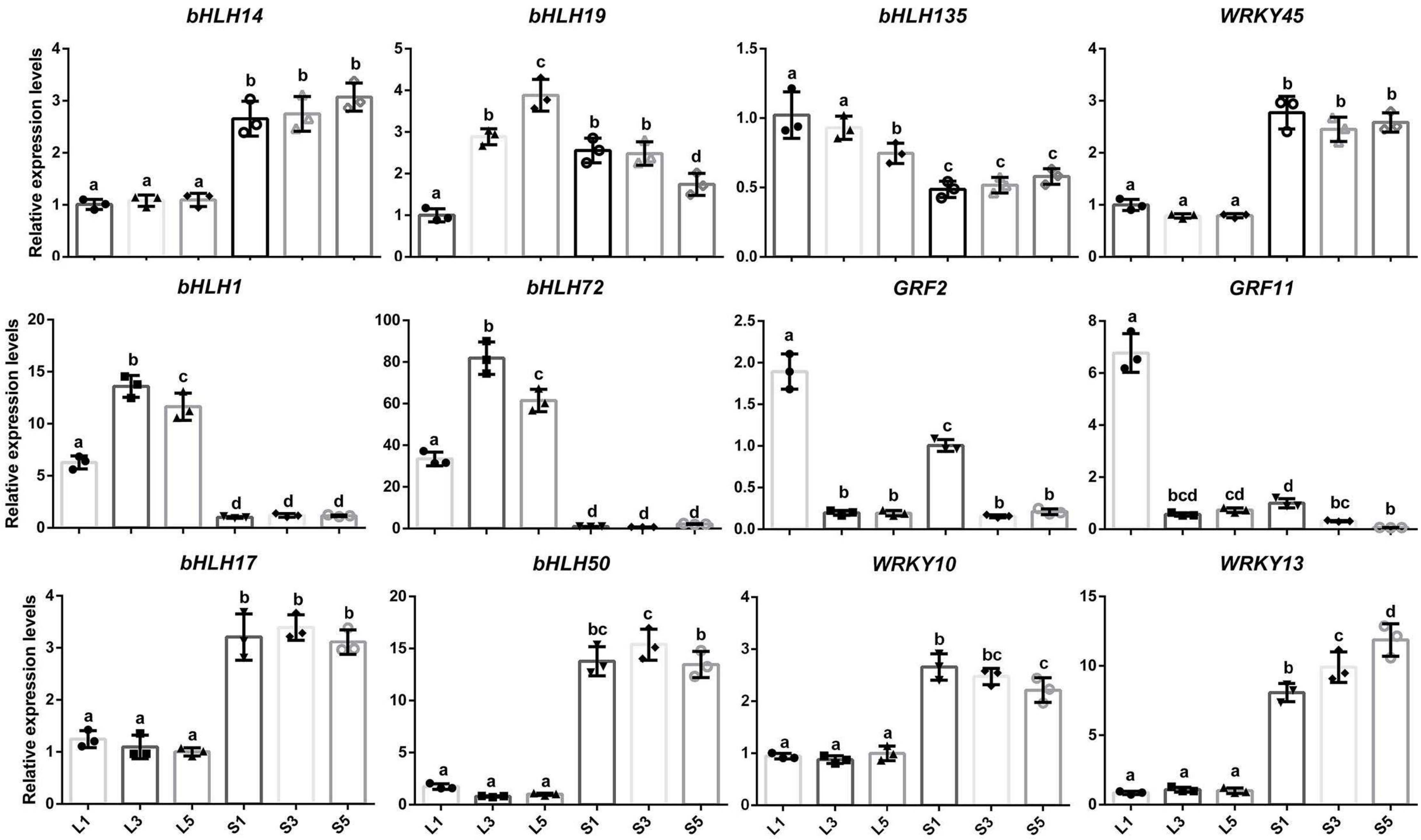
bHLH14
bHLH19
bHLH135
WRKY45
bHLH1
bHLH72
GRF2
GRF11
bHLH17
bHLH50
WRKY10
WRKY13
Relative expression levels
L1
L3
L5
S1
S3
S5

Supplementary Figure 23. Relative expression levels of some bHLH, WRKY and GRF transcription factors in different development stage of leaf (L1, L3 and L5) or stem (S1, S3 and S5). Expression profiles of these genes were analyzed using RT-qPCR analysis. The poplar *Ubiquitin* (*UBQ*) gene was used as an internal control and gene expression profiles were evaluated using the $2^{-\Delta\Delta Ct}$ method. Three biological replicates for each tissue were analyzed. Error bars represent the SD of the mean (n=3). Different letters above bars represent statistically significant differences between groups ($p < 0.05$) as determined by one-way ANOVA followed by Dunnett's test. In these groups, the same letter indicates that there is no significant difference between the two groups, different letters indicate that there is a significant difference between the two groups. For *bHLH14*, *bHLH19*, *bHLH135*, and *WRKY45*, the relative expression level of L1 stage was set to be 1. For *bHLH1*, *bHLH72*, *GRF2*, and *GRF11*, the relative expression level of S1 stage was set to be 1. For *bHLH17*, *bHLH50*, *WRKY10*, and *WRKY13*, the relative expression level of L5 stage was set to be 1.

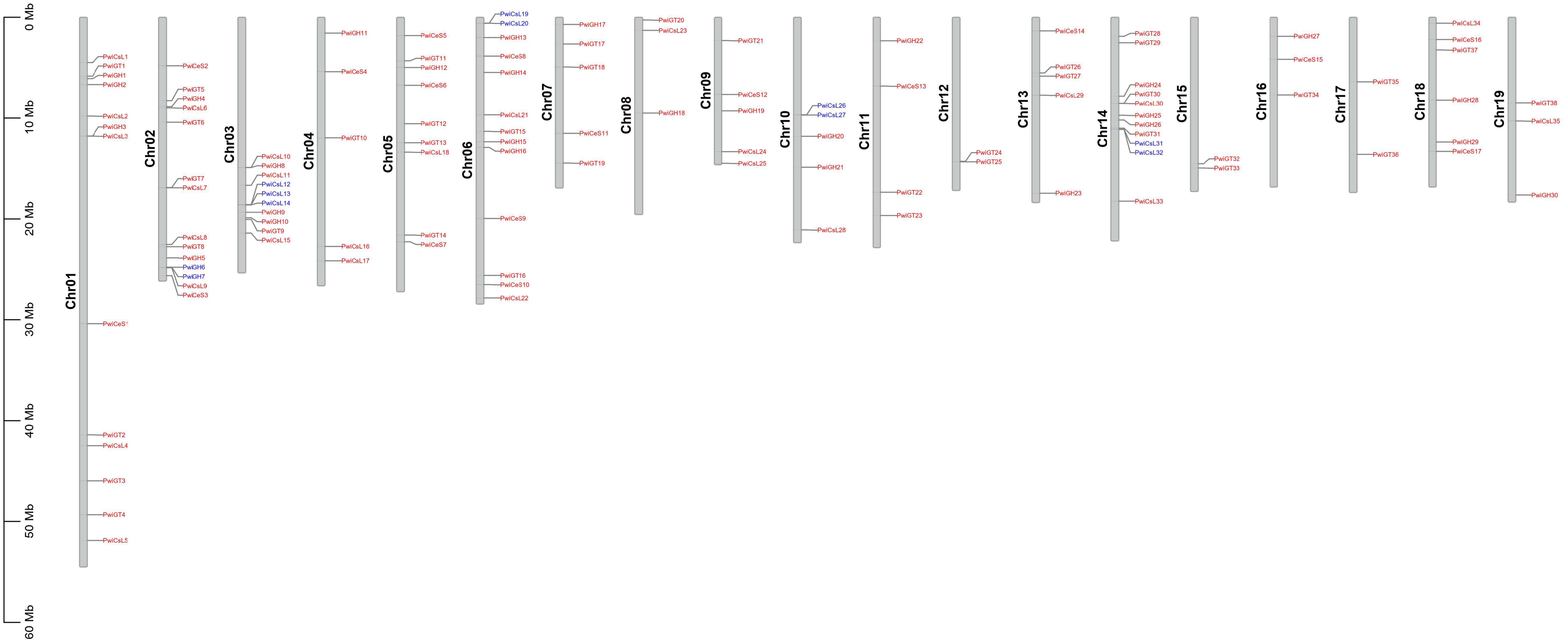

0 Mb
10 Mb
20 Mb
30 Mb
40 Mb
50 Mb
60 Mb
Chr01
PwiCsL1
PwiGT1
PwiGH1
PwiGH2
PwiCsL2
PwiGH3
PwiCsL3
PwiCeS1
PwiGT2
PwiCsL4
PwiGT3
PwiGT4
PwiCsL5
Chr02
PwiCeS2
PwiGT5
PwiGH4
PwiCsL6
PwiGT6
PwiGT7
PwiCsL7
PwiCsL8
PwiGT8
PwiGH5
PwiGH6
PwiGH7
PwiCsL9
PwiCeS3
Chr03
PwiCsL10
PwiGH8
PwiCsL11
PwiCsL12
PwiCsL13
PwiCsL14
PwiGH9
PwiGH10
PwiGT9
PwiCsL15
Chr04
PwiGH11
PwiCeS4
PwiGT10
PwiCsL16
PwiCsL17
Chr05
PwiCeS5
PwiGT11
PwiGH12
PwiCeS6
PwiGT12
PwiGT13
PwiCsL18
PwiGT14
PwiCeS7
Chr06
PwiCsL19
PwiCsL20
PwiGH13
PwiCeS8
PwiGH14
PwiCsL21
PwiGT15
PwiGH15
PwiGH16
PwiCeS9
PwiGT16
PwiCeS10
PwiCsL22
Chr07
PwiGH17
PwiGT17
PwiGT18
PwiCeS11
PwiGT19
Chr08
PwiGT20
PwiCsL23
PwiGH18
Chr09
PwiGT21
PwiCeS12
PwiGH19
PwiCsL24
PwiCsL25
Chr10
PwiCsL26
PwiCsL27
PwiGH20
PwiGH21
PwiCsL28
Chr11
PwiGH22
PwiCeS13
PwiGT22
PwiGT23
Chr12
PwiGT24
PwiGT25
Chr13
PwiCeS14
PwiGT26
PwiGT27
PwiCsL29
PwiGH23
Chr14
PwiGT28
PwiGT29
PwiGH24
PwiGT30
PwiCsL30
PwiGH25
PwiGH26
PwiGT31
PwiCsL31
PwiCsL32
PwiCsL33
Chr15
PwiGT32
PwiGT33
Chr16
PwiGH27
PwiCeS15
PwiGT34
Chr17
PwiGT35
PwiGT36
Chr18
PwiCsL34
PwiCeS16
PwiGT37
PwiGH28
PwiGH29
PwiCeS17
Chr19
PwiGT38
PwiCsL35
PwiGH30

Supplementary Figure 24. Chromosomal locations of *Populus wilsonii* Ces (Pwi Ces), Csl (PwiCsl), GH (PwiGH) and GT (PwiGT) gene families. Tandem duplicates are represented by blue font.

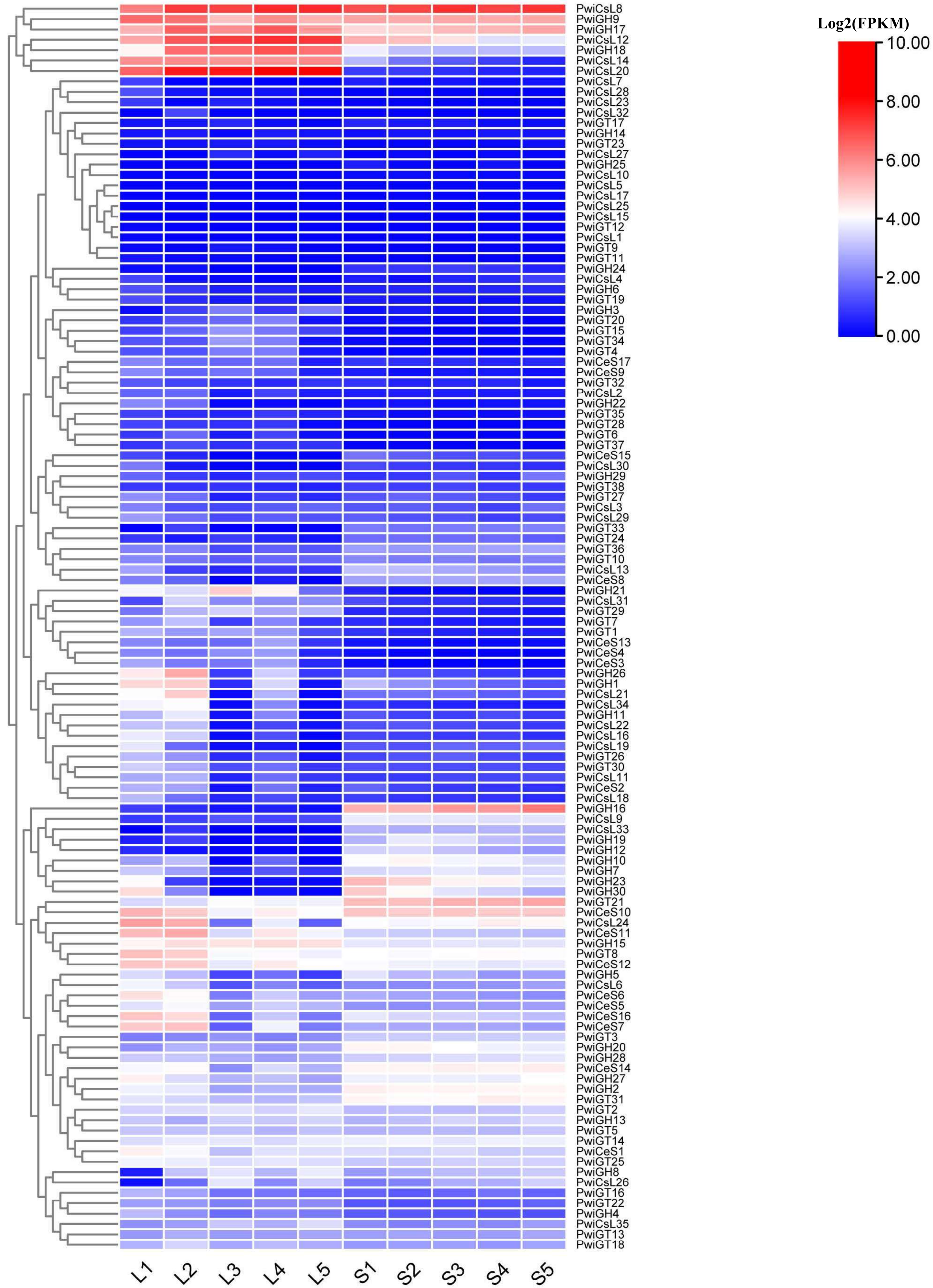
Log2(FPKM)
10.00
8.00
6.00
4.00
2.00
0.00
PwiCsL8
PwiGH9
PwiGH17
PwiCsL12
PwiGH18
PwiCsL14
PwiCsL20
PwiCsL7
PwiCsL28
PwiCsL23
PwiCsL32
PwiGT17
PwiGH14
PwiGT23
PwiCsL27
PwiGH25
PwiCsL10
PwiCsL5
PwiCsL17
PwiCsL25
PwiCsL15
PwiGT12
PwiCsL1
PwiGT9
PwiGT11
PwiGH24
PwiCsL4
PwiGH6
PwiGT19
PwiGH3
PwiGT20
PwiGT15
PwiGT34
PwiGT4
PwiCeS17
PwiCeS9
PwiGT32
PwiCsL2
PwiGH22
PwiGT35
PwiGT28
PwiGT6
PwiGT37
PwiCeS15
PwiCsL30
PwiGH29
PwiGT38
PwiGT27
PwiCsL3
PwiCsL29
PwiGT33
PwiGT24
PwiGT36
PwiGT10
PwiCsL13
PwiCeS8
PwiGH21
PwiCsL31
PwiGT29
PwiGT7
PwiGT1
PwiCeS13
PwiCeS4
PwiCeS3
PwiGH26
PwiGH1
PwiCsL21
PwiCsL34
PwiGH11
PwiCsL22
PwiCsL16
PwiCsL19
PwiGT26
PwiGT30
PwiCsL11
PwiCeS2
PwiCsL18
PwiGH16
PwiCsL9
PwiCsL33
PwiGH19
PwiGH12
PwiGH10
PwiGH7
PwiGH23
PwiGH30
PwiGT21
PwiCeS10
PwiCsL24
PwiCeS11
PwiGH15
PwiGT8
PwiCeS12
PwiGH5
PwiCsL6
PwiCeS6
PwiCeS5
PwiCeS16
PwiCeS7
PwiGT3
PwiGH20
PwiGH28
PwiCeS14
PwiGH27
PwiGH2
PwiGT31
PwiGT2
PwiGH13
PwiGT5
PwiGT14
PwiCeS1
PwiGT25
PwiGH8
PwiCsL26
PwiGT16
PwiGT22
PwiGH4
PwiCsL35
PwiGT13
PwiGT18
L1
L2
L3
L4
L5
S1
S2
S3
S4
S5

Supplementary Figure 25. Heatmap showing the expression level of four Cellulose synthesis gene in different development stages of leaf and stem in *Populus wilsonii*. The FPKM values are log2-based. Red and blue indicate high and low expression levels, respectively. Each sample for every development stage had three biological replicates.

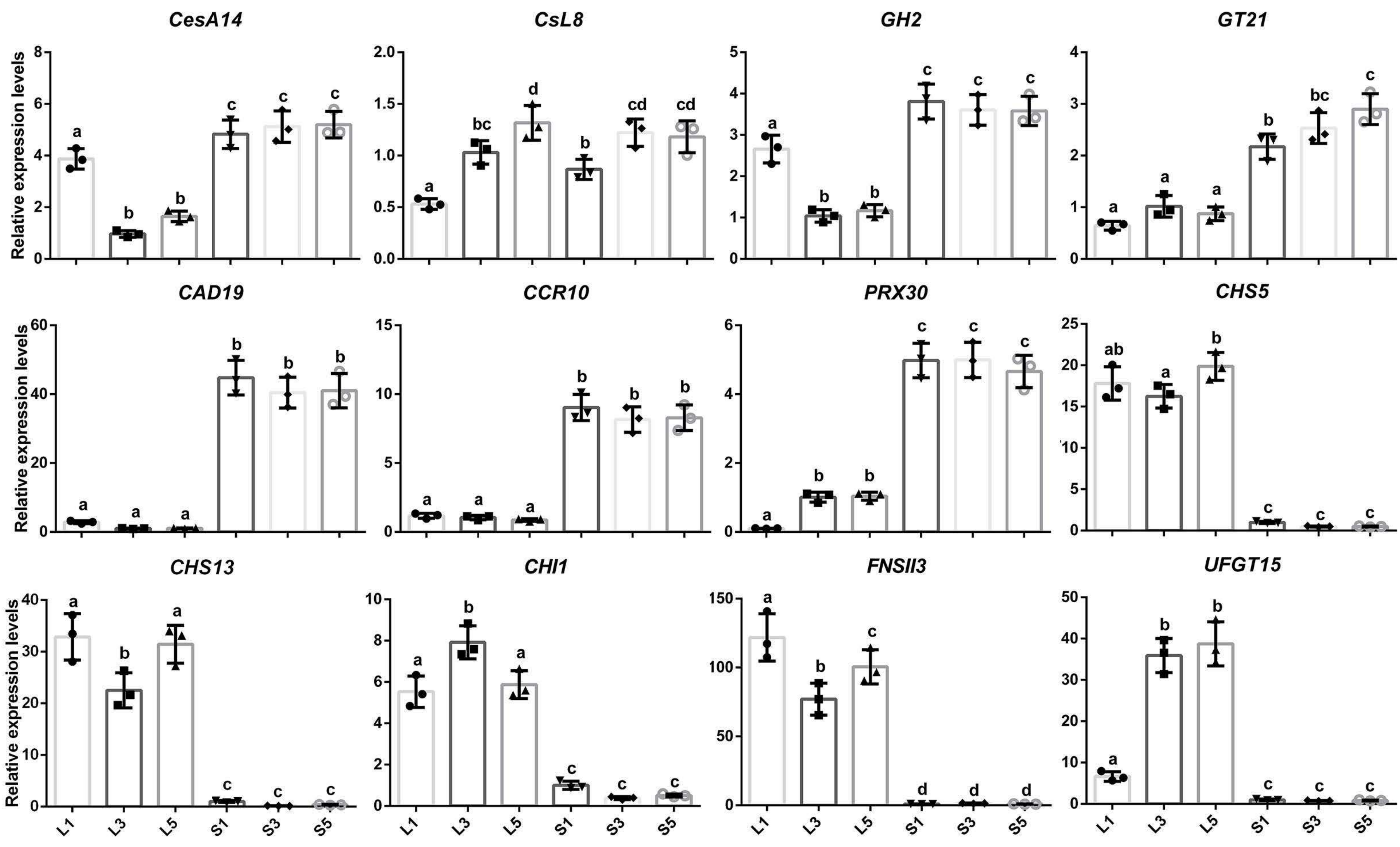

CesA14
CsL8
GH2
GT21
CAD19
CCR10
PRX30
CHS5
CHS13
CHI1
FNSII3
UFGT15
Relative expression levels
L1
L3
L5
S1
S3
S5

Supplementary Figure 26. Relative expression levels of some key enzyme genes involved in cellulose, hemicellulose, lignin and flavonoid biosynthesis in different development stage of leaf (L1, L3 and L5) or stem (S1, S3 and S5). Expression profiles of these genes were analyzed using RT-qPCR analysis. The poplar *Ubiquitin* (*UBQ*) gene was used as an internal control and gene expression profiles were evaluated using the $2^{-\Delta\Delta Ct}$ method. Three biological replicates for each tissue were analyzed. Error bars represent the SD of the mean (n=3). Different letters above bars represent statistically significant differences between groups ($p < 0.05$) as determined by one-way ANOVA followed by Dunnett's test. In these groups, the same letter indicates that there is no significant difference between the two groups, different letters indicate that there is a significant difference between the two groups. For *CesA14*, *CsL8*, *GH2*, and *GT21*, the relative expression level of L3 stage was set to be 1. For *CAD19*, *CCR10*, and *PRX30*, the relative expression level of L3 stage was set to be 1. For *CHS5*, *CHS13*, *CHI1*, *FNSII3*, and *UFGT15*, the relative expression level of S1 stage was set to be 1.

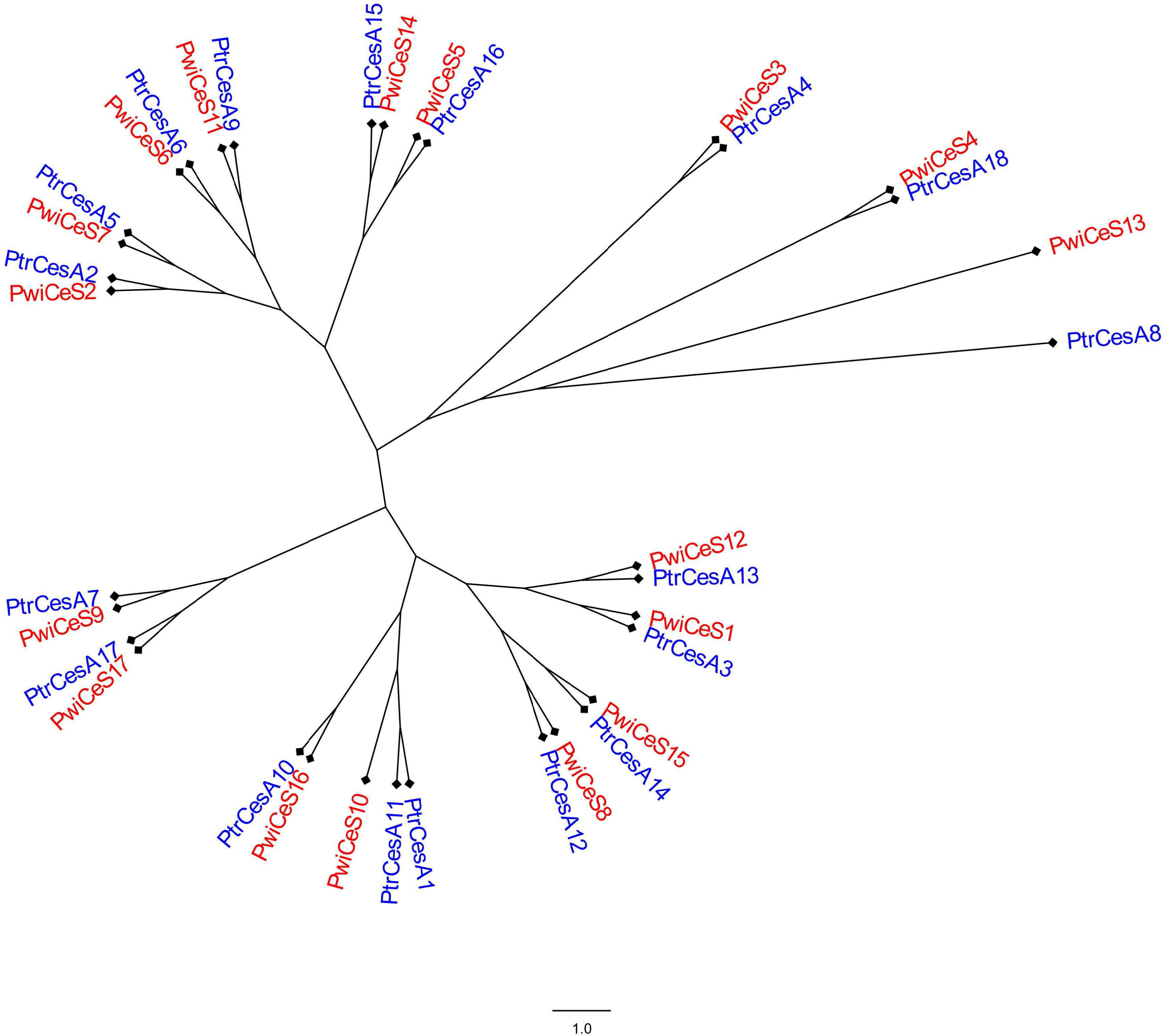

PtrCesA15
PwiCeS14
PwiCeS5
PtrCesA16
PtrCesA9
PwiCeS11
PtrCesA6
PwiCeS6
PtrCesA5
PwiCeS7
PtrCesA2
PwiCeS2
PwiCeS3
PtrCesA4
PwiCeS4
PtrCesA18
PwiCeS13
PtrCesA8
PwiCeS12
PtrCesA13
PwiCeS1
PtrCesA3
PwiCeS15
PtrCesA14
PwiCeS8
PtrCesA12
PtrCesA1
PtrCesA11
PwiCeS10
PtrCesA10
PwiCeS16
PtrCesA7
PwiCeS9
PtrCesA17
PwiCeS17
1.0

Supplementary Figure 27. Phylogenetic analysis of Cellulose genes in *Populus wilsonii* (red) and *Populus trichocarpa* (blue).

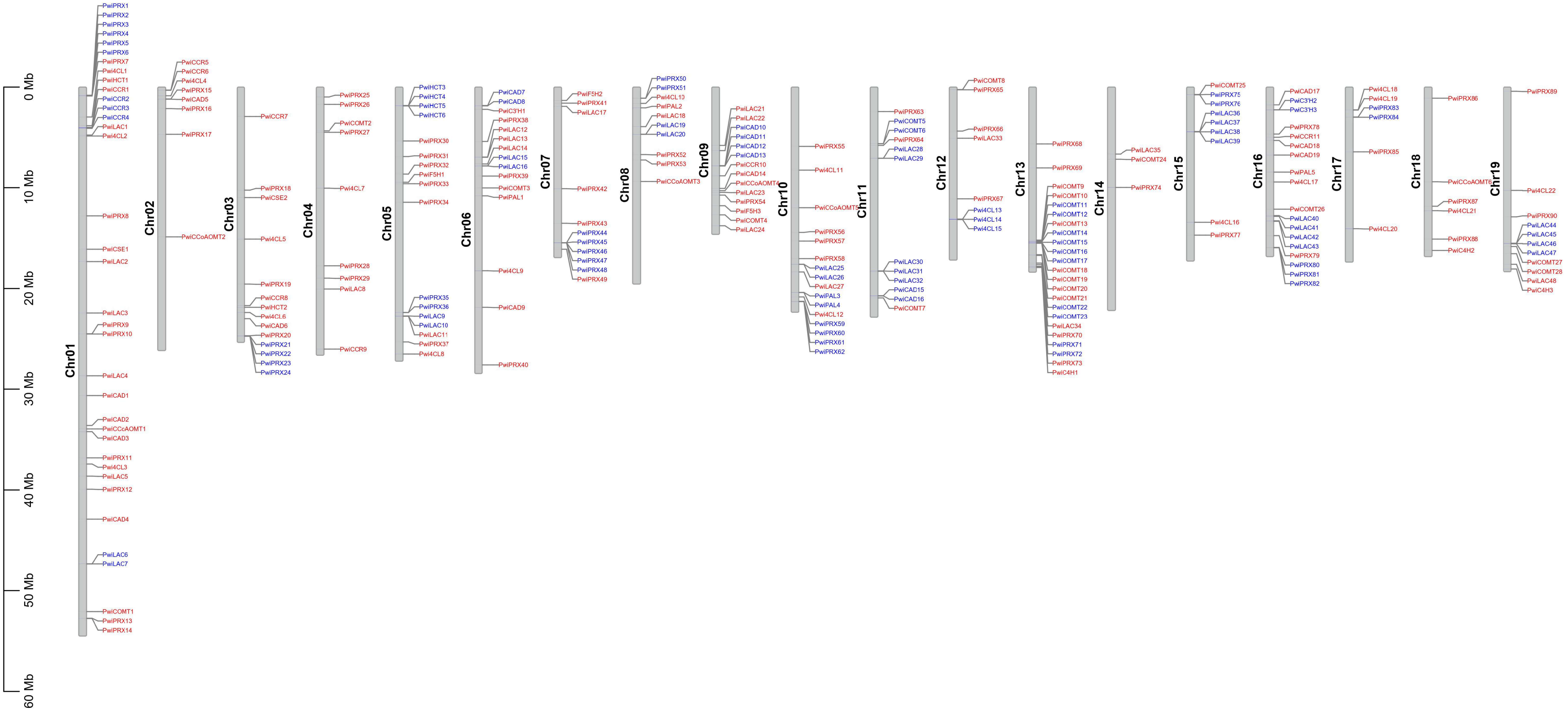
0 Mb
10 Mb
20 Mb
30 Mb
40 Mb
50 Mb
60 Mb
Chr01
PwiPRX1
PwiPRX2
PwiPRX3
PwiPRX4
PwiPRX5
PwiPRX6
PwiPRX7
Pwi4CL1
PwiHCT1
PwiCCR1
PwiCCR2
PwiCCR3
PwiCCR4
PwiLAC1
Pwi4CL2
PwiPRX8
PwiCSE1
PwiLAC2
PwiLAC3
PwiPRX9
PwiPRX10
PwiLAC4
PwiCAD1
PwiCAD2
PwiCCoAOMT1
PwiCAD3
PwiPRX11
Pwi4CL3
PwiLAC5
PwiPRX12
PwiCAD4
PwiLAC6
PwiLAC7
PwiCOMT1
PwiPRX13
PwiPRX14
Chr02
PwiCCR5
PwiCCR6
Pwi4CL4
PwiPRX15
PwiCAD5
PwiPRX16
PwiPRX17
PwiCCoAOMT2
Chr03
PwiCCR7
PwiPRX18
PwiCSE2
Pwi4CL5
PwiPRX19
PwiCCR8
PwiHCT2
Pwi4CL6
PwiCAD6
PwiPRX20
PwiPRX21
PwiPRX22
PwiPRX23
PwiPRX24
Chr04
PwiPRX25
PwiPRX26
PwiCOMT2
PwiPRX27
Pwi4CL7
PwiPRX28
PwiPRX29
PwiLAC8
PwiCCR9
Chr05
PwiHCT3
PwiHCT4
PwiHCT5
PwiHCT6
PwiPRX30
PwiPRX31
PwiPRX32
PwiF5H1
PwiPRX33
PwiPRX34
PwiPRX35
PwiPRX36
PwiLAC9
PwiLAC10
PwiLAC11
PwiPRX37
Pwi4CL8
Chr06
PwiCAD7
PwiCAD8
PwiC3'H1
PwiPRX38
PwiLAC12
PwiLAC13
PwiLAC14
PwiLAC15
PwiLAC16
PwiPRX39
PwiCOMT3
PwiPAL1
Pwi4CL9
PwiCAD9
PwiPRX40
Chr07
PwiF5H2
PwiPRX41
PwiLAC17
PwiPRX42
PwiPRX43
PwiPRX44
PwiPRX45
PwiPRX46
PwiPRX47
PwiPRX48
PwiPRX49
Chr08
PwiPRX50
PwiPRX51
Pwi4CL10
PwiPAL2
PwiLAC18
PwiLAC19
PwiLAC20
PwiPRX52
PwiPRX53
PwiCCoAOMT3
Chr09
PwiLAC21
PwiLAC22
PwiCAD10
PwiCAD11
PwiCAD12
PwiCAD13
PwiCCR10
PwiCAD14
PwiCCoAOMT4
PwiLAC23
PwiPRX54
PwiF5H3
PwiCOMT4
PwiLAC24
Chr10
PwiPRX55
Pwi4CL11
PwiCCoAOMT5
PwiPRX56
PwiPRX57
PwiPRX58
PwiLAC25
PwiLAC26
PwiLAC27
PwiPAL3
PwiPAL4
Pwi4CL12
PwiPRX59
PwiPRX60
PwiPRX61
PwiPRX62
Chr11
PwiPRX63
PwiCOMT5
PwiCOMT6
PwiPRX64
PwiLAC28
PwiLAC29
PwiLAC30
PwiLAC31
PwiLAC32
PwiCAD15
PwiCAD16
PwiCOMT7
Chr12
PwiCOMT8
PwiPRX65
PwiPRX66
PwiLAC33
PwiPRX67
Pwi4CL13
Pwi4CL14
Pwi4CL15
Chr13
PwiPRX68
PwiPRX69
PwiCOMT9
PwiCOMT10
PwiCOMT11
PwiCOMT12
PwiCOMT13
PwiCOMT14
PwiCOMT15
PwiCOMT16
PwiCOMT17
PwiCOMT18
PwiCOMT19
PwiCOMT20
PwiCOMT21
PwiCOMT22
PwiCOMT23
PwiLAC34
PwiPRX70
PwiPRX71
PwiPRX72
PwiPRX73
PwiC4H1
Chr14
PwiLAC35
PwiCOMT24
PwiPRX74
Chr15
PwiCOMT25
PwiPRX75
PwiPRX76
PwiLAC36
PwiLAC37
PwiLAC38
PwiLAC39
Pwi4CL16
PwiPRX77
Chr16
PwiCAD17
PwiC3'H2
PwiC3'H3
PwiPRX78
PwiCCR11
PwiCAD18
PwiCAD19
PwiPAL5
Pwi4CL17
PwiCOMT26
PwiLAC40
PwiLAC41
PwiLAC42
PwiLAC43
PwiPRX79
PwiPRX80
PwiPRX81
PwiPRX82
Chr17
Pwi4CL18
Pwi4CL19
PwiPRX83
PwiPRX84
PwiPRX85
Pwi4CL20
Chr18
PwiPRX86
PwiCCoAOMT6
PwiPRX87
Pwi4CL21
PwiPRX88
PwiC4H2
Chr19
PwiPRX89
Pwi4CL22
PwiPRX90
PwiLAC44
PwiLAC45
PwiLAC46
PwiLAC47
PwiCOMT27
PwiCOMT28
PwiLAC48
PwiC4H3

Supplementary Figure 28. Chromosomal locations of Lignin synthesis gene families in *Populus wilsonii*. Tandem duplicates are represented by blue font.

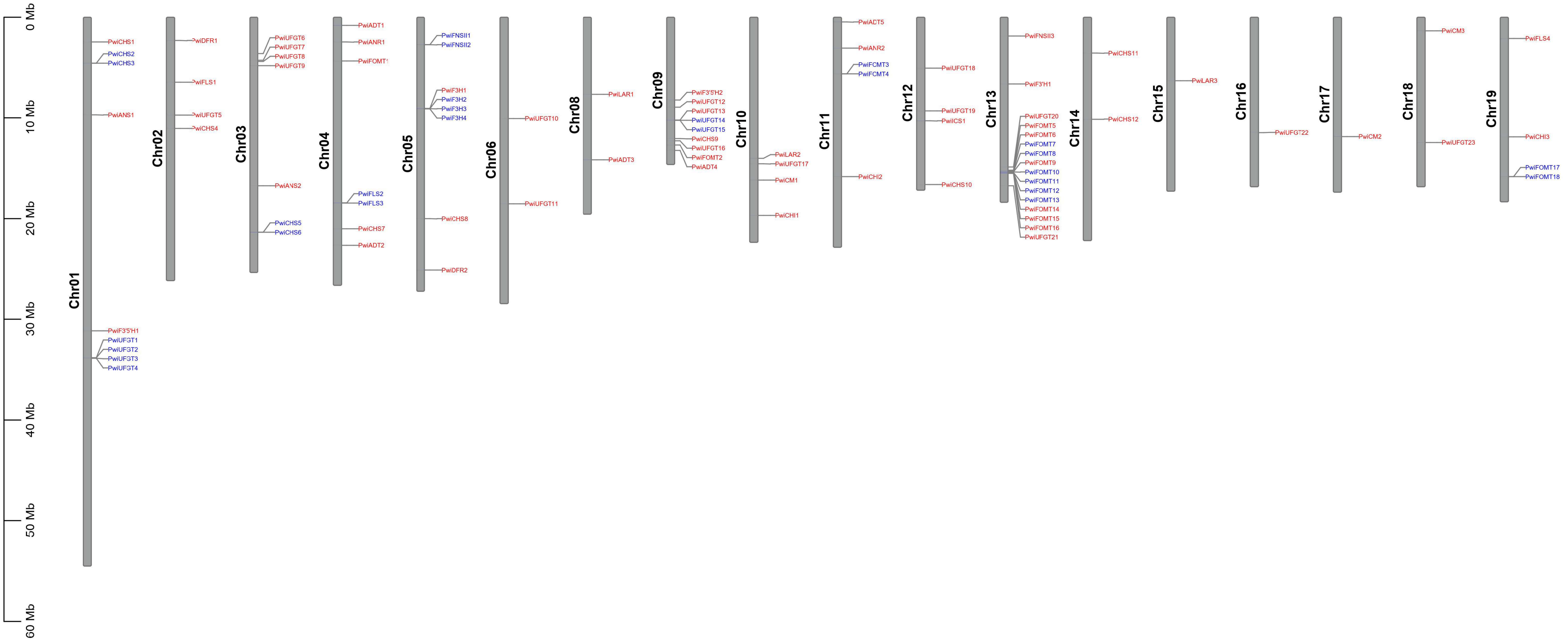
0 Mb
10 Mb
20 Mb
30 Mb
40 Mb
50 Mb
60 Mb
Chr01
PwiCHS1
PwiCHS2
PwiCHS3
PwiANS1
PwiF3'5'H1
PwiUFGT1
PwiUFGT2
PwiUFGT3
PwiUFGT4
Chr02
PwiDFR1
PwiFLS1
PwiUFGT5
PwiCHS4
Chr03
PwiUFGT6
PwiUFGT7
PwiUFGT8
PwiUFGT9
PwiANS2
PwiCHS5
PwiCHS6
Chr04
PwiADT1
PwiANR1
PwiFOMT1
PwiFLS2
PwiFLS3
PwiCHS7
PwiADT2
Chr05
PwiFNSII1
PwiFNSII2
PwiF3H1
PwiF3H2
PwiF3H3
PwiF3H4
PwiCHS8
PwiDFR2
Chr06
PwiUFGT10
PwiUFGT11
Chr08
PwiLAR1
PwiADT3
Chr09
PwiF3'5'H2
PwiUFGT12
PwiUFGT13
PwiUFGT14
PwiUFGT15
PwiCHS9
PwiUFGT16
PwiFOMT2
PwiADT4
Chr10
PwiLAR2
PwiUFGT17
PwiCM1
PwiCHI1
Chr11
PwiADT5
PwiANR2
PwiFOMT3
PwiFOMT4
PwiCHI2
Chr12
PwiUFGT18
PwiUFGT19
PwiICS1
PwiCHS10
Chr13
PwiFNSII3
PwiF3'H1
PwiUFGT20
PwiFOMT5
PwiFOMT6
PwiFOMT7
PwiFOMT8
PwiFOMT9
PwiFOMT10
PwiFOMT11
PwiFOMT12
PwiFOMT13
PwiFOMT14
PwiFOMT15
PwiFOMT16
PwiUFGT21
Chr14
PwiCHS11
PwiCHS12
Chr15
PwiLAR3
Chr16
PwiUFGT22
Chr17
PwiCM2
Chr18
PwiCM3
PwiUFGT23
Chr19
PwiFLS4
PwiCHI3
PwiFOMT17
PwiFOMT18

Supplementary Figure 29. Chromosomal locations of flavonoids synthesis gene families in *Populus wilsonii*. Tandem duplicates are represented by blue font.

Supplementary Tables

Supplementary Table 1. Overview of sequencing data in *Populus wilsonii*.

Reads Type	Total bases (Gb)	Total reads	Reads N50 (Bp)	Mean Length (bp)	Longest Read (bp)	Function
Subreads	475.88	33,348,682	15,645	14,270	387,426	Assembly
CCS read	30.4	2,019,886	15,075	15,049	36,256	
**Read length (bp)**	**Clean data (Gb)**	**Total Read Pairs**	**Mapped Reads**	**Unique Mapped Read Pairs**	**Q30(%)**	**Function**
150_150	54.30	181,253,818	320,850,736.00	148,575,876	93.24	Assembly
**Read length (bp)**	**Library**	**Data (Gb)**	**Filtered data (Gb)**	**Depth (×)**	**Q30 (%)**	**Function**
150_150	350 bp	32.10	27.37	62.28	93.08	Survey

Note: Assuming the genome size of *Populus wilsonii* is 440 Mb.

Supplementary Table 2. Estimation of *Populus wilsonii* genome size based on 19 K-mer statistics.

Clean data	Q30 (%)	Sequencing depth（X）	GC content (%)
27.37Gb	93.08	62.28	35.14
**k-mer**	**Main peak depth of k-mer**	**Estimated genome Size**	**Heterozygote frequencies**
19	26	439.39 Mb	0.42%

Supplementary Table 3. Overview of the type of Hi-C date.

Type	Number	Ratio (%)
Total Read Pairs	181,253,818	100
Mapped Reads	160,425,368	88.5
Unique Paired Alignments	148,575,876	100
Valid Interaction Pairs	83,433,287	56
Dangling End Pairs	50456221	34
Re-ligation Pairs	3,318,277	2
Self-cycle Pairs	992,030	1
Dumped Pairs	10,376,061	6.98

Supplementary Table 4. Evaluation of Benchmarking Universal Single-Copy Orthologs (BUSCO) and Gene Space Coverage Using Core Eukaryotic Gene Mapping Approach (CEGMA) in *Populus wilsonii* genome.

Type	Number	Percent (%)
Complete BUSCOs (C)	1,591	98.57
Complete and single-copy BUSCOs (S)	1338	82.9
Complete and duplicated BUSCOs (D)	253	15.68
Fragmented BUSCOs (F)	4	0.25
Missing BUSCOs (M)	19	1.18
Total BUSCO groups searched	1,614	-
Number of 458 CEG present in assembly	452	98.69
Number of 248 highly conserved CEGs present	233	93.95

Supplementary Table 5. The clean data of NovaSeq6000 and PacBio mapped to reference genome.

Data Type	Total Reads	Map Reads	Map Rate (%)	Coverage at least 10X (%)	Coverage at least 20X (%)	Coverage at least 30X (%)
NovaSeq 6000	214,444,004	213,643,498	99.63	99.32	99.02	98.63
PacBio CCS	2,019,886	2,018,736	99.94	99.76	99.64	99.43

Supplementary Table 6. Statistics of samples used for RNA-Seq sequencing.

Samples	Organ	Clean reads pair	Clean bases(bp)	Mapped Reads	Rate	Q30(%)
bud	Leaf bud	25782466	7702341298	48,095,328	93.27%	0.9471
flowers	Flowers	23045560	6891330014	43,569,328	94.53%	0.9409
flower_bud	Flower bud	25916157	7748961270	49,085,581	94.70%	0.936
leaf	Leaf	24515553	7312556460	46,805,278	95.46%	0.9416
phloem	Phloem	36645427	10965845302	56,998,075	77.77%	0.9428
root	Root	24735027	7387490240	44,133,181	89.21%	0.942
xylem	Xylem	34560191	10286616988	61,182,923	88.52%	0.9347
L1-1	Leaf	21575061	6441369768	40,721,905	94.37%	0.9411
L1-2	Leaf	24247276	7237877412	45,436,030	93.69%	0.9395
L1-3	Leaf	22712343	6778821166	41,167,377	90.63%	0.9459
L2-1	Leaf	27146929	8097157696	51,268,633	94.43%	0.9427
L2-2	Leaf	23155374	6915249116	43,694,345	94.35%	0.9384
L2-3	Leaf	19902165	5947708884	37,704,181	94.72%	0.9423
L3-1	Leaf	24424927	7296339670	45,941,555	94.05%	0.9418
L3-2	Leaf	19758603	5910762376	37,453,786	94.78%	0.9404
L3-3	Leaf	19639126	5862503642	37,025,030	94.26%	0.9348
L4-1	Leaf	24120701	7194014772	45,692,780	94.72%	0.9429
L4-2	Leaf	25240674	7538611890	47,583,169	94.26%	0.9408
L4-3	Leaf	25578929	7634602964	48,485,165	94.78%	0.944
L5-1	Leaf	23520561	7019809848	44,475,965	94.55%	0.9414
L5-2	Leaf	25735037	7672017466	48,427,848	94.09%	0.942
L5-3	Leaf	20405526	6095447932	38,349,309	93.97%	0.9448
S1-1	Stem	22800715	6812742042	42,980,243	94.25%	0.9106
S1-2	Stem	22836218	6828492916	42,832,487	93.78%	0.9087
S1-3	Stem	22735787	6802845532	43,103,569	94.79%	0.921
S2-1	Stem	19265879	5762734554	35,745,131	92.77%	0.9195
S2-2	Stem	20989232	6280314128	39,433,787	93.94%	0.9196
S2-3	Stem	22355168	6687921290	41,716,552	93.30%	0.9119
S3-1	Stem	22815587	6826112342	43,028,897	94.30%	0.9139
S3-2	Stem	22348949	6673546576	41,673,496	93.23%	0.9125
S3-3	Stem	22312298	6672517446	41,339,946	92.64%	0.911
S4-1	Stem	22410173	6701281464	41,481,410	92.55%	0.9154
S4-2	Stem	23031748	6888293788	43,192,485	93.77%	0.9225
S4-3	Stem	22370476	6686367792	41,527,114	92.82%	0.9142
S5-1	Stem	19240953	5757499444	34,723,306	90.23%	0.9194
S5-2	Stem	19540603	5844492582	36,650,703	93.78%	0.9177
S5-3	Stem	22383929	6696077498	41,118,786	91.85%	0.9144

Supplementary Table 7. Statistics of repeat contents in *Populus wilsonii* genome.

Type	Number	Length(bp)	Rate (%)
Retroelement	213,466	122,441,381	25.65
LTR/Gypsy	91,583	61,579,392	12.9
LTR/Copia	46,293	36,611,814	7.67
LTR/others	50624	18,528,103	3.89
LINE	23,221	5,536,069	1.16
SINE	1,745	186,003	0.04
DNA transposon	176,705	59,967,949	12.56
tandem repeat	318,075	56,056,995	11.74
Total	708,246	238,466,325	49.96

Supplementary Table 8. Statistics on gene information of different species.

	Species	*P. trichocarpa*	*P. wilsonii*	*A. thaliana*	*P. deltoides*	*P. euphratica*
Gene	Number	34,699	38,054	27,628	44,853	36,426
	Length(bp)	126,544,386	133,688,470	65,552,768	136,044,577	126,214,227
	Average Length(bp)	3646.92	3513.13	2372.69	3033.12	3464.95
Exon	Number	177,938	198,300	141,285	212,920	201,601
	Average Number	5.13	5.21	5.11	4.75	5.53
	Length(bp)	43,737,174	68,941,684	33,622,504	48,528,846	54,848,341
	Average Length(bp)	1260.47	1811.68	1216.97	1081.95	1505.75
CDS	Number	177,938	190,693	141,285	212,920	193,128
	Average Number	5.13	5.01	5.11	4.75	5.3
	Length(bp)	43,737,174	50,284,380	33,622,504	48,528,846	41,373,495
	Average Length(bp)	1260.47	1321.4	1216.97	1081.95	1135.82
Intron	Number	143,239	160,246	113,657	168,067	165,175
	Average Number	4.13	4.21	4.11	3.75	4.53
	Length(bp)	82,807,212	64,746,786	31,930,264	87,515,731	71,365,886
	Average Length(bp)	2386.44	1701.44	1155.72	1951.17	1959.2

Supplementary Table 9. Evaluation the Gene Completeness by Benchmarking Universal Single-Copy Orthologs (BUSCO) in *Populus wilsonii* genome.

Type	Number	Percent
Complete BUSCOs(C)	1,558	96.53%
Complete and single-copy BUSCOs(S)	1,317	81.60%
Complete and duplicated BUSCOs(D)	241	14.93%
Fragmented BUSCOs(F)	37	2.29%
Missing BUSCOs(M)	19	1.18%
Total Lineage BUSCOs	1,614	

Supplementary Table 10. Statistical information of gene function annotation

Annotation Database	Annotated Number	Annotated Ratio (%)
GO Annotation	30,762	80.84
KEGG Annotation	27,948	73.44
KOG Annotation	20,082	52.77
Pfam Annotation	31,963	83.99
Swissprot Annotation	30,446	80.01
TrEMBL Annotation	37,391	98.26
eggNOG Annotation	31,448	82.64
nr Annotation	37,435	98.37
All Annotated	37,547	98.67

Supplementary Table 11. Statistical information of non-coding RNA in *P. wilsonii* genome

Type	Number	Length(bp)
rRNA	8,186	11,428,311
tRNA	5,066	381,283
miRNA	126	14,650
snRNA	101	14,963
snoRNA	474	51,055
Total	13,953	11,890,262

Supplementary Table 12. Species and gene sets used in this study

Species	Abbreviations	Database	Version
Populus euphratica	*P. euphratica*	BIG Submission	V3.1
Populus alba	*P. alba*	BIG Submission	V3.1.1
Populus deltoides	*P. deltoides*	phytozome	v2.1
Populus trichocarpa	*P. trichocarpa*	phytozome	v4.1
Arabidopsis thaliana	*A. thaliana*	phytozome	Araport11
Oryza sativa	*O. sativa*	phytozome	V7_JGI
Manihot esculenta	*M. esculenta*	phytozome	v7.1
Vitis vinifera	*V. vinifera*	phytozome	V2.1
Salix purpurea	*S. purpurea*	phytozome	v5_1

Supplementary Table 13. Orthologous Groups in *P. wilso*nii and other plant species

Item	*A. thaliana*	*M. esculenta*	*O. sativa*	*P. alba*	*P. deltoides*	*P. euphratica*	*P. trichocarpa*	*P. wilsonii*	*S. purpurea*	*V. vinifera*
Number of genes	27,628	33,849	39,049	40,213	44,853	36,426	34,699	38,054	35,125	31,845
Number of genes in orthogroups	18,385	30,635	21,136	32,537	39,386	30,433	32,862	35,618	33,268	23,665
Number of unassigned genes	9,243	3,214	17,913	7,676	5,467	5,993	1,837	2,436	1,857	8,180
Percentage of genes in orthogroups	66.5	90.5	54.1	80.9	87.8	83.5	94.7	93.6	94.7	74.3
Percentage of unassigned genes	33.5	9.5	45.9	19.1	12.2	16.5	5.3	6.4	5.3	25.7
Number of orthogroups containing species	12,663	18,195	12,589	20,107	22,715	20,499	20,630	21,338	19,011	16,934
Percentage of orthogroups containing species	33.6	48.2	33.4	53.3	60.2	54.3	54.7	56.6	50.4	44.9
Number of species-specific orthogroups	2,520	697	4,120	512	521	227	56	140	453	1,001
Number of genes in species-specific orthogroups	6,823	2,669	11,486	1,211	1,306	504	156	1,072	2,531	3,448
Percentage of genes in species-specific orthogroups	24.7	7.9	29.4	3	2.9	1.4	0.4	2.8	7.2	10.8

Supplementary Table 14. Gene copy number of key genes involved in cellulose synthesis in *Populus wilsonii* and 4 other *Salicaceae* species.

Gene	*S. purpurea*	*P. alba*	*P. deltoides*	*P. euphratica*	*P. trichocarpa*	*P. wilsonii*
CES	16	21	17	23	18	17
CsL	27	41	36	37	36	35
GH	34	36	35	33	33	30
GT	38	42	47	35	40	38

Supplementary Table 15. Gene copy number of key genes involved in lignin and flavonoids synthesis in *Populus wilsonii* and 4 other *Salicaceae* species.

Type	Gene	*S. purpurea*	*P. alba*	*P. deltoides*	*P. euphratica*	*P. trichocarpa*	*P. wilsonii*
Commom	CM	4	5	3	3	3	3
	ADT	8	6	8	5	5	5
	PAL	5	6	6	6	5	5
	C4H	4	4	4	3	4	3
	4CL	16	16	23	21	19	22
Lignin	HCT	3	5	9	4	5	6
	LAC	44	43	45	40	49	48
	PRX	95	98	113	81	97	90
	CCoCAMT	4	6	6	5	6	6
	CCR	15	8	22	12	13	11
	COMT	15	33	42	30	33	28
	CSE	2	2	2	2	2	2
	F5H	4	4	3	2	3	3
	C3H	4	3	3	1	3	3
	CAD	16	18	18	15	18	19
Flavonoids	ANR	1	1	2	2	2	2
	ANS	2	3	2	2	2	2
	CHI	3	5	3	4	4	3
	CHS	10	15	17	10	13	12
	DFR	2	2	4	2	2	2
	FLS	4	5	3	4	4	4
	F3'H	1	1	1	1	1	1
	F3'5'H	1	3	2	1	2	2
	FNS II	2	1	1	0	3	3
	FOMT	12	20	19	16	18	18
	ICS	1	1	1	2	1	1
	LAR	2	3	3	4	3	3
	F3H	3	2	4	1	2	4
	UFGT	22	28	16	25	26	23

Supplementary Table 16. Primers used in this study for qRT-PCR analysis.

Gene Name	Gene ID	Forward Primer sequence (5´-3´)	Reverse primer sequence (5´-3´)
bHLH1	Pwi01G001590	ACGAATTTGAACCAAAACCCACT	TATGCATGCACACCTTGCAC
GH2	Pwi01G006950	ACAGGCATGATGGTTTCCGT	CCTGACAAAGCCACAAGTGC
GRF2	Pwi01G010250	TGGAACGGAACATGAGCCAG	GCACTACCGATTCCAGGGAG
GDSL-3	Pwi01G017170	CGGGGAGCAGTGACTTTCTT	GGCATCAGCGTTGATCCTTG
WRKY10	Pwi01G032650	CATGCAACCCCAATTACGGC	GCCTGAGATGGATGAACGCT
bHLH14	Pwi01G037250	TCGGTGCGGAAATTGATGGA	AGGCTCCAAGATAGAGCCCA
WRKY13	Pwi02G004060	AGCTGCACACCTACAATGTCA	GAACTCTGATCCGAGGAGCG
bHLH17	Pwi02G004200	AAATGTTGGTGCTGCCATGC	GACGGATCTTCCAAGCACCT
bHLH19	Pwi02G005150	GTGGCAAGATGGGTAAGCCT	ATTGTGCTGGAATCCTCGGG
CsL8	Pwi02G020210	GGTTCATGTCCCCGTGATGTAA	CAACCCACCTCTTGACCCC
CHS5	Pwi03G016840	AGCCTAACCGAGGCATTCAA	TTCTCTGGTTTCAGCGCCAA
CEPR1	Pwi04G000150	TATCGGGTGGAGTCCCATCA	GCCTCCATACACGCTCTTCA
SAUR21	Pwi04G014630	TGGCCATCCTTCTTAAGGGTA	AAGGAAGTGTAAGACCGCCC
bHLH50	Pwi05G000190	GCTACGGCCTGTAGTTCACA	TTGCAAATGCTCACCAGCCT
PRX30	Pwi05G006230	GTTTGTCCAGGCACGGTTTC	GTGACCCTGAAAGGGCTACC
bHLH72	Pwi06G006550	CGTGGTCCCCAACATTACCA	TCAGCTTGAATTCTTCTCTCTTGC
WRKY45	Pwi06G011910	GAACCAGGAGGAGGATCCAA	ACCTTCTTTTTCACTGGACAACT
bHLH80	Pwi07G002170	TGGCAGGTGAAGATGTGACC	TGCCCGCACATGAATGTAGT
GT21	Pwi09G001110	AGGAACTCTACTCGACCAGG	TTGGAAGCCTCACGGTTCTG
CCR10	Pwi09G007320	TACTAACGGCGGCAAAGGAG	CTGTACCCGGATTCACCACC
UFGT15	Pwi09G009350	AGCTTTATTGGGCGGGGA	CCCCCTTCTTCAACTGCCTTC
ERF109	Pwi09G009600	TTAACACTGCAGAGGAGGCG	AGAACGCATTGTCTTTCCCCA
LBD15	Pwi10G015830	GAGGCAAGGCTCAGAGATCC	GGAGGAGAATTGGCAGCGTA
CHI1	Pwi10G018290	TGGCGCAGGGGCGAG	CCGAATATTGGAGGCCCGT
GRF11	Pwi12G001090	AACACCAGCAGCACCCCAG	TGGCAAGAGTTGGACTGCTATT
CesA14	Pwi13G001780	CTTTGCCCCAAGTCCCTCTC	CCACTGCCACCAATAGGAGC
FNSII3	Pwi13G002490	ACGAAGAAAGGAACTGCGCC	GTTATCAGTCCCTGAACACCTGA
WRKY76	Pwi14G007110	GGACTTGGGCTCTAAGGACG	TGGGGTCTGCACATTTTGGT
bHLH135	Pwi14G008540	TGCAACAAGACCAACAAGCG	AGGAACTATCTGACTTGCCGA
CHS13	Pwi14G011620	TCAGCTGCACAAACCATCCT	ACCAGGGTGTGCAATCCAAA
CAD19	Pwi16G007310	AAGCCTCTTGAGCTTCCTGC	CCAAACGTTCCATTGCCGTA
CCR4	Pwi19G008730	ATGTCACACCTCTCACTCCAC	TCAACGACATTCCTCGGCAC
UBQ	Pwi01G037400	GTTGATTTTTGCTGGGAAGC	GATCTTGGCCTTCACGTTGT
