# Supplementary material for: Chromosome-scale genome assembly provides insights into the molecular mechanisms of tissue development of Populus wilsonii
Source: Commun Biol. 2022 Oct 25;5:1125. doi: 10.1038/s42003-022-04106-0 (PMC9596445; doi:10.1038/s42003-022-04106-0)
Supplement: Supplementary file 2 — Description of Additional Supplementary Files [file 42003_2022_4106_MOESM2_ESM.docx]

File name: Supplementary Data 1

Description: Transcription factors identified in DEGs during leaf development and stem development.

File name: Supplementary Data 2

Description: Annotated of top 50 key genes in the turquoise module and green module in leaf.

File name: Supplementary Data 3

Description: Annotated of top 50 key genes in the brown module and turquoise module in stem.

File name: Supplementary Data 4

Description: Transcription factors identified in *P. wilsonii* and other plant species.

File name: Supplementary Data 5

Description: Gene categories used from all the species.
