# Supplementary material for: Chromosome-scale genome assembly provides insights into the molecular mechanisms of tissue development of Populus wilsonii
Source: Commun Biol. 2022 Oct 25;5:1125. doi: 10.1038/s42003-022-04106-0 (PMC9596445; doi:10.1038/s42003-022-04106-0)
Supplement: Supplementary file 4 — reporting summary [file 42003_2022_4106_MOESM4_ESM.pdf]

nature portfolio

Corresponding author(s): Shengkui Zhang

Last updated by author(s): Oct 12, 2022

Reporting Summary

Nature Portfolio wishes to improve the reproducibility of the work that we publish. This form provides structure for consistency and transparency in reporting. For further information on Nature Portfolio policies, see our Editorial Policies and the Editorial Policy Checklist.

Statistics

For all statistical analyses, confirm that the following items are present in the figure legend, table legend, main text, or Methods section.

n/a	Confirmed	
☐	☒	The exact sample size (*n*) for each experimental group/condition, given as a discrete number and unit of measurement
☐	☒	A statement on whether measurements were taken from distinct samples or whether the same sample was measured repeatedly
☒	☐	The statistical test(s) used AND whether they are one- or two-sided<br>*Only common tests should be described solely by name; describe more complex techniques in the Methods section.*
☒	☐	A description of all covariates tested
☒	☐	A description of any assumptions or corrections, such as tests of normality and adjustment for multiple comparisons
☒	☐	A full description of the statistical parameters including central tendency (e.g. means) or other basic estimates (e.g. regression coefficient) AND variation (e.g. standard deviation) or associated estimates of uncertainty (e.g. confidence intervals)
☒	☐	For null hypothesis testing, the test statistic (e.g. *F*, *t*, *r*) with confidence intervals, effect sizes, degrees of freedom and *P* value noted<br>*Give P values as exact values whenever suitable.*
☐	☒	For Bayesian analysis, information on the choice of priors and Markov chain Monte Carlo settings
☒	☐	For hierarchical and complex designs, identification of the appropriate level for tests and full reporting of outcomes
☐	☒	Estimates of effect sizes (e.g. Cohen's *d*, Pearson's *r*), indicating how they were calculated

Our web collection on statistics for biologists contains articles on many of the points above.

Software and code

Policy information about availability of computer code

Data collection	Total genomic DNA was isolated from fresh leaves of P. wilsonii, and part of it was used to construct circular consensus sequencing (CCS) libraries and sequence them using a PacBio Sequal Ⅱ platform, and other portions were used for library preparation with insert sizes of 350 bp and sequenced them using the Illumina NovaSeq 6000 platform. For RNA sequencing, the RNA Samples from five development stages of leaf (2nd, 4th ,6th, 8th, and 10th internode leaf corresponding to L1—L5) and stem (the annual, biennial, triennial, four-year-old, and five-year-old stems corresponding to S1, S2, S3, S4, and S5) in P. wilsonii, and the used for library preparation and sequenced them using the Illumina NovaSeq 6000 platform
Data analysis	The P. wilsonii genome was assembled by using Hifiasm (V 0.12) with default parameters, and LACHESIS were used to divided into subgroups and sorted and oriented into pseudomolecules. HISAT2 was used to map the RNA-seq clean reads to the genome with the default parameters. Transcripts were assembled using StringTie. Gene expression was measured as fragments per kilobase of transcript per million fragments mapped by using StringTie.

For manuscripts utilizing custom algorithms or software that are central to the research but not yet described in published literature, software must be made available to editors and reviewers. We strongly encourage code deposition in a community repository (e.g. GitHub). See the Nature Portfolio guidelines for submitting code & software for further information.

Data

Policy information about availability of data

All manuscripts must include a data availability statement. This statement should provide the following information, where applicable:

- Accession codes, unique identifiers, or web links for publicly available datasets
- A description of any restrictions on data availability
- For clinical datasets or third party data, please ensure that the statement adheres to our policy

The whole genome sequence data (including Illumina reads, Pacbio reads and Hi-C interaction reads), transcriptomes data of different tissues used to assist genome annotation and transcriptomes data of different tissues at different developmental stages in this study have been deposited in the Genome Sequence Archive in the BIG Data Center, Beijing Institute of Genomics (BIG), Chinese Academy of Sciences, under project number PRJCA008004. The final assembly genome and genome annotation information and other data have been deposited in figshare (https://figshare.com/articles/dataset/Populus_wilsonii_genome_data/19143284) which is publicly accessible at this website.

Human research participants

Policy information about studies involving human research participants and Sex and Gender in Research.

Reporting on sex and gender	No reporting on sex and gender in this manuscript
Population characteristics	No population characteristics in this manuscript
Recruitment	No recruitment in this manuscript
Ethics oversight	No ethics oversight in this manuscirpt

Note that full information on the approval of the study protocol must also be provided in the manuscript.

Field-specific reporting

Please select the one below that is the best fit for your research. If you are not sure, read the appropriate sections before making your selection.

☒ Life sciences ☐ Behavioural & social sciences ☐ Ecological, evolutionary & environmental sciences

For a reference copy of the document with all sections, see nature.com/documents/nr-reporting-summary-flat.pdf

Life sciences study design

All studies must disclose on these points even when the disclosure is negative.

Sample size	We produced genome data of P. wilsonii.
Data exclusions	No data exclusions in this manuscript.
Replication	No replication in this manuscript.
Randomization	No randomization in this manuscript as samples were not allocated into experimental groups.
Blinding	No blinding in this manuscript as the data were not allocated into groups.

Reporting for specific materials, systems and methods

We require information from authors about some types of materials, experimental systems and methods used in many studies. Here, indicate whether each material, system or method listed is relevant to your study. If you are not sure if a list item applies to your research, read the appropriate section before selecting a response.

Materials & experimental systems

n/a	Involved in the study
☒	☐ Antibodies
☒	☐ Eukaryotic cell lines
☒	☐ Palaeontology and archaeology
☒	☐ Animals and other organisms
☒	☐ Clinical data
☒	☐ Dual use research of concern

Methods

n/a	Involved in the study
☒	☐ ChIP-seq
☒	☐ Flow cytometry
☒	☐ MRI-based neuroimaging
